# Diversity Hotspots and Vulnerability of Pine Species in the Sierra Madre Occidental, Western Mexico

**DOI:** 10.1002/ece3.71743

**Published:** 2025-07-09

**Authors:** Lizeth Ruacho‐González, José Javier Corral‐Rivas, Jesús Guadalupe González‐Gallegos, M. Socorro González‐Elizondo, Pablito Marcelo López‐Serrano, Jaime Briseño‐Reyes

**Affiliations:** ^1^ Programa Institucional de Doctorado en Ciencias Agropecuarias y Forestales Universidad Juárez del Estado de Durango Durango Mexico; ^2^ Centro Interdisciplinario de Investigación Para el Desarrollo Integral Regional Unidad Durango Instituto Politécnico Nacional Durango Mexico; ^3^ Facultad de Ciencias Forestales y Ambientales Universidad Juárez del Estado de Durango Durango Mexico; ^4^ Instituto de Silvicultura e Industria de la Madera Universidad Juárez del Estado de Durango Durango Mexico

**Keywords:** climate change, diversity, environmental variables, *Pinus*, risk, species distribution modeling

## Abstract

Mexico is a global hotspot for pine diversity, with approximately 60 taxa mainly found in temperate mountainous areas. For this reason, they face increasing threats from climate change, particularly within the Sierra Madre Occidental (SMO). This study aimed to identify areas with the highest potential pine richness in SMO and determine which species are at risk, using current and future potential distribution models. The distribution of 19 pine species was modeled based on 7020 records. Environmental variables were carefully selected from WorldClim 2.1, by multicollinearity elimination and then selecting those with a strong correlation to species presence (Spearman's coefficient *ρ* > 0.70). Models were developed in MaxEnt using the GISS E2‐1‐G model to predict future distributions in the “245” Shared Socioeconomic Pathway in a “middle of the road” scenario for 2040, 2060, 2080, and 2100. The resulting species‐specific models were overlayed to identify areas most suitable to host the greatest number of species across all projections. Results indicate that the areas with environmental characteristics to host the highest number of pine species are located on the upper portions of the SMO's western slopes. Models indicate a general trend of decreasing pine distribution area in the region; with estimated reductions ranging from 22% to 29% for *Pinus durangensis*, *P. devoniana*, and *P. engelmannii* . The worst situation is for *P. brachyptera* which may practically disappear by 2060. Despite habitat reductions, species such as *P. cembroides* , *P. devoniana*, and *P. oocarpa* show a tendency to migrate to higher altitudes. The principal conclusions are: pine species hotspot areas are situated on the SMO's western slopes in Durango state, just above the Tropic of Cancer. Approximately 95% of the studied pine species in SMO will show reductions by the end of the century, and *P. brachyptera* is at risk of extirpation in Mexico.

## Graphical abstract

The present study employs a modeling approach to examine the current and future distribution of 19 pine species in the Sierra Madre Occidental, Mexico. This was done in order to identify areas with high potential for pine species richness and to determine which species are at risk due to climate change under different projections.

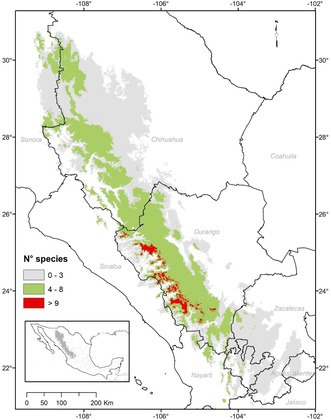

## 1 Introduction

Mexico is the country with the largest number of pine species in the world, a reflection of the evolutionary and biogeographic complexity of this group of plants. Pines are distributed mainly in the mountainous regions of the national territory, with exceptions as *Pinus* *caribaea* var. *hondurensis* (Sénécl.) W.H. Barrett & Golfari in lowlands of the tropical region in southeastern Mexico (Sánchez‐González 2008). *Pinus* is a common element, often physiognomically dominant, in different vegetation types such as pine forests or scrub, mixed forests (pine‐oak or other conifers), cloud forests, and woodlands (Rzedowski 1978; Gernandt and Pérez‐de la Rosa 2014). As an important part of these communities, pines perform vital ecosystem functions such as climate regulation, nutrient circulation, carbon storage, air and water filtration, soil retention, and provide habitat for a wide variety of flora and fauna (Sánchez‐González 2008; Galicia and Zarco‐Arista 2014). In this way, pine trees are fundamental pillars of biodiversity and play a crucial role in maintaining the ecosystem services that sustain life in Mexico.

In addition to being an invaluable ecological resource, pine forests are an important source of economic wealth. Pine wood is widely used in construction, furniture manufacturing, paper production, and other industrial sectors (Galicia et al. 2018; Monárrez‐González et al. 2018). In 2021, pine wood production was $8080 million Mexican pesos, with Durango and Chihuahua as the states with the highest production, collectively yielding 3.3 million m^3^r (round timber), which represents more than 50% of the national pine production, establishing the Sierra Madre Occidental (SMO) as the most important pine forest reservoir in the country (SEMARNAT 2021).

However, the SMO is also among the most threatened ecoregions in North America (Olson and Dinerstein 2002). The high demand for timber has generated intense pressure on pine forests, resulting in deforestation in some areas. This deforestation may contribute to climate change and risk the integrity of this valuable natural heritage (Bonilla‐Moheno and Aide 2020). For instance, extreme heat events have led to bark beetle (*Dendroctonus*) infestations that have killed large areas of pine forest, which were already stressed by drought (González‐Elizondo, González‐Elizondo, Ruacho‐González, et al. 2013). Therefore, the SMO, which has 46% of the pine species reported for the country (González‐Elizondo et al. 2012), is presented as an ideal study model to evaluate the future behavior of pine species and to propose effective conservation strategies.

The SMO is home to approximately 24 pine tree species, with some, such as *P. durangensis* Martínez and *P. leiophylla* Schiede ex Schltdl. & Cham., being widely distributed (Aceves‐Rangel et al. 2018). Others, such as *P. devoniana* Lindl., reach their northernmost distribution boundary in southern SMO, whereas *P. brachyptera* reaches its southern distribution at northern Chihuahua and Sonora (Willyard et al. 2021). Additionally, there are taxa that occur in very specific areas such as *P. maximartinezii* Rzed. (González‐Elizondo et al. 2011, 2012) or *P. yecorensis* var. *sinaloensis* Debreczy & I. Rácz, due to the orographic and climatic heterogeneity of the region and the environmental requirements that characterize each species (González‐Elizondo et al. 2012; Macías‐Rodríguez et al. 2017).

One method for identifying the environmental variables associated with the distribution of tree species is the development of species distribution models (SDM) (Mod et al. 2016; Guisan et al. 2017). The application of these conservation‐oriented methods allows for the prediction of the potential distribution of the suitable climatic habitat of the tree species under different environmental scenarios. Furthermore, it has enabled the identification of areas with a high diversity of species, the proposal of the creation of new reserves, and the identification of the most vulnerable species under climate change scenarios (Pearson 2010; Guisan et al. 2017).

The information obtained from the SDM for pine species represents a significant instrument in forest monitoring, as it facilitates the formulation of management plans over the medium and long‐term and the protection of pine forests for future generations (Montoya‐Jiménez et al. 2022). The main objective of this study was to model the current and future potential distribution of 19 pine tree species in the SMO, to detect (a) areas with high richness, and (b) species at risk due to climate change. The models are expected to reveal changes in the distribution of species, with possible implications for their long‐term survival, as well as for the floristic composition of the communities. The results will facilitate the formulation of practical recommendations to improve forest management and biodiversity conservation in the SMO.

## 2 Methods

### 2.1 Study Area

The SMO is the largest mountain range in Mexico. Located in the northwest of the country, it runs along 1200 km from northern Sonora and Chihuahua (30°35′ N) to northern Jalisco (21°00′ N). In its temperate zones there are communities of pine‐oak, pine, oak, mixed conifers, and woodlands, which range from 350 m asl on the western slopes and 1450 m asl on the east, up to 3340 m asl on the highest peaks (González‐Elizondo et al. 2012). The SMO sustains temperate elements derived of the Madro‐Tertiary geoflora (Axelrod 1958) such as *Pinus*, *Quercus*, and *Arbutus*. Four ecoregions are recognized, based on physiographic, climatic, and floristic criteria: (a) Madrean (M) *sensu stricto*, which occurs on the higher, cold‐temperate areas of the sierra, (b) Tropical Madrean (TM) on the upper western slopes where the climate is moister and warmer than inland, (c) Xerophylous Madrean (XM) on the eastern foothills and branches of the cordillera, having a continental, dry and extreme climate, and (d) Madrean Archipelago (MA) in the northern zone of the sierra (González‐Elizondo, González‐Elizondo, Tena‐Flores, et al. 2013). The latter is formed by a group of isolated mountains (Sky islands) that are a region of connection between the SMO and the Rocky Mountains, so they were excluded from the main mountain range and from the analysis in this work (Figure S1.1).

### 2.2 Species Data

The species that are being considered in this study are those proposed by Gernandt and Pérez‐de la Rosa (2014), and also we have contemplated some species pines identified in Willyard et al. (2021), which include *P. brachyptera* Engelm and *P. cooperi* C.E. Blanco for the study area.

The species records were obtained from electronic databases of MONAFOR (National System of Forest and Soil Monitoring Sites, http://fcfposgrado.ujed.mx/monafor), CIIDIR Herbarium, SEINet, and the SNIB (Mexico's National Biodiversity Information System), which includes iNaturalistMX observations and national and international scientific collections. A prior selection of the data were made, which excluded reports with incomplete or null coordinates, insufficient curatorial degree, and proximity of other data less than a 1 km radius. Finally, a total of 7020 records were selected for 19 of the 24 *Pinus* taxa that are distributed in SMO (Table S2.1). Although there are studies with small size records, in this case it was decided to follow the recommendations of Guisan et al. (2017) and exclude those pines species with less than 15 records: *Pinus* cf. *hartwegii* Lindl., *P. maximartinezii* , *P. praetermissa* Styles and McVaugh, *P*. × *reflexa* (Engelm.) Engelm., and *P. yecorensis* var. *sinaloensis*. Other excluded species that have been reported for the study area and the reasons why they are not considered here are presented in Table S2.2. Thus, all selected pine species for modeling fitting showed occurrences only inside the SMO.

### 2.3 Environmental Variables

A total of 32 environmental variables for 1970–2000 were obtained from WorldClim 2.1 (Fick and Hijmans 2017) at a 30 arc‐second resolution (Table S2.3). Solar radiation and water vapor pressure are only available as monthly rasters, so new rasters were generated using the “cell statistic” tool in Arcmap 10.8.

Values of each variable were extracted by points of species datasets. To address collinearity among environmental variables, those exhibiting a high correlation (i.e., those with variance inflation factor values above 5) were detected and eliminated in RStudio with the “vifcor” function of the “usdm” package (Naimi 2017). Once collinearity between variables was eliminated, a Spearman correlation analysis was applied to identify the variables with a strong relationship to the species' presence. Thus, only the variables showing a correlation coefficient (*ρ*) or greater than 0.7 with the species' presence were chosen for model development because they are considered to have a strong relationship with the species' occurrence.

In a preliminary exploration of predictive variables, we also considered vegetation types of the SMO (González‐Elizondo et al. 2012), soil type data (INEGI 2005), as well as soil properties (Cruz‐Cárdenas et al. 2014); however, none of these variables exhibited a significant relationship with our species distribution data, likely due to their elaboration at larger geographic scales.

### 2.4 Species Distributions Models

Species distribution models were developed in Maxent v.3.4.4 software (Phillips et al. 2006) using 10 cross validation replicates (Elith et al. 2011), 500 iterations, and 10,000 background points as default parameters. The functions *Extrapolate* and *Do clamping* were deactivated to reduce improbable predictions (Elith et al. 2011; Owens et al. 2013). Otherwise, *Maximum training sensitivity plus specificity* was used as threshold setting, and output results were logistic type (Liu et al. 2016; Wan et al. 2016). The contributions of the main variables for each model were estimated with a Jackknife test. Models were evaluated using the area under the curve (AUC) values from Maxent and the Receiver operating characteristic (ROC) test (*p* ≤ 0.5); the latter was executed at CONABIO's NicheToolBox site with 1000 replicates and *E* = 0.05 (Osorio‐Olvera et al. 2020). Details of the modeling process are given in Table S2.4.

Model GISS E2‐1‐G from CMIP6 was selected to create future scenarios; this model has the lowest mean square error for mean annual temperature and the third lowest for mean precipitation in Mexico (Pérez‐Figueroa 2021). The 245 scenario was used for prediction of 2040, 2060, 2080, and 2100, which represents the current trend with a moderate development; it uses a temperature increase estimate of 2.0°C–2.9°C for the next 100 years, based on the past century trends. We have chosen the SSP245 scenario because it allows for the assessment of the vulnerability of Mexican pine species in a context of a moderate scenario that still considers significant climate warming (Riahi et al. 2017; Dyderski et al. 2025). Model projections are crucial to implement conservation and management strategies that offer long‐term survival possibilities for these species.

### 2.5 Model Overlapping

The ENMTools software (Warren et al. 2021) was then used to compare the current distribution area between pine species using the niche overlap tool. *D* index was the statistics used to measure the similarity of the distribution of suitable habitat for a pair of models. Values of 0 represent little overlap and values closer to 1 represent the highest degree of overlap. In RStudio, the ‘pheatmap’ function (Kolde 2019) was used to visualize the similarity matrix and produce a clustering dendrogram.

In order to detect the areas with conditions to harbor the greatest number of species, an overlap of the models was performed for each period, using Map Algebra Tool from ArcGis 10.8. New rasters were classified by categories according to the number of species per pixel. The top areas, as well as the poorest, were identified for each time period and continuously from the present to the end of the century.

## 3 Results

### 3.1 Selection of Environmental Variables

The elimination of highly correlated predictive variables from the species‐specific models reduced them from 32 to 17, with the largest number for *P. lumholtzii*, and down to 9 and 8 for *P. devoniana* and *P. durangensis* , respectively (Table S2.5). The most frequently used variables were Bio9 (mean temperature of driest quarter), Bio14 (precipitation of the driest month), Bio15 (precipitation seasonality) and Bio18 (precipitation of warmest quarter), as well as mean solar radiation (Smean) and minimal solar radiation (Smin). In contrast, neither solar radiation range (Srang) nor minimal water vapor pressure (Vmin), nor slope and aspect were correlated with the presence of any studied pine species, so they were not used in the models.

### 3.2 Species Current Distribution Models

A total of 19 models were developed, from which 15 were significant (*p* < 0.05). The non‐significance for four out of 19 models means that they are not better than a random prediction (Phillips et al. 2006). Even so, predictions are considered accurate since the AUC values are above 0.7, ROC partial is ranged from 1.5 to 2.0, and training omission rate is below 17% (Table 1).

**TABLE 1:** Maxent results for pine species distribution models in Sierra Madre Occidental.

Species	*N*	Test AUC	Training AUC	Partial ROC	Logistic threshold	Fractional predicted area	Training omission	*p*
*Pinus arizonica*	593	0.871	0.879	1.64	0.2538	0.2803	0.1046	< 0.0001
*P. brachyptera*	21	0.956	0.974	1.88	0.331	0.0643	0.0476	0.0588
*P. cembroides*	261	0.823	0.855	1.54	0.3716	0.3093	0.1235	< 0.0001
*P. chihuahuana*	326	0.789	0.812	1.49	0.4006	0.2689	0.2410	< 0.0001
*P. cooperi*	361	0.932	0.938	1.77	0.1768	0.01723	0.0649	< 0.0001
*P. devoniana*	51	0.935	0.954	1.83	0.2266	0.1391	0.0261	0.0111
*P. discolor*	35	0.824	0.897	1.68	0.2661	0.1673	0.1528	0.1200
*P. douglasiana*	67	0.926	0.957	1.80	0.2547	0.1055	0.0747	0.0008
*P. durangensis*	1127	0.854	0.858	1.66	0.2747	0.3226	0.0710	< 0.0001
*P. engelmannii*	682	0.815	0.827	1.53	0.3538	0.3361	0.1562	< 0.0001
*P. herrerae*	294	0.92	0.93	1.74	0.2146	0.1849	0.0729	< 0.0001
*P. leiophylla*	758	0.843	0.853	1.61	0.2985	0.3232	0.0998	< 0.0001
*P. lumholtzii*	598	0.834	0.851	1.61	0.3762	0.2928	0.1141	< 0.0001
*P. luzmariae*	35	0.913	0.955	1.82	0.305	0.122	0.0412	0.0512
*P. maximinoi*	44	0.942	0.968	1.87	0.4116	0.0884	0.0353	0.1007
*P. oocarpa*	60	0.919	0.944	1.77	0.1821	0.1793	0.0296	0.0084
*P. strobiformis*	1058	0.863	0.866	1.65	0.3123	0.285	0.1134	< 0.0001
*P. teocote*	605	0.905	0.9137	1.75	0.2668	0.1799	0.0915	< 0.0001
*P. yecorensis*	44	0.976	0.981	1.89	0.3774	0.0388	0.0630	0.0009

#### 3.2.1 Important Variable Groups

Species distribution models can be divided into four main groups by the degree of influence of the predictive variables: temperature (Bio4–Bio11), precipitation (Bio12–Bio19), solar radiation (Smean‐Sstd) and a mix of these without evident dominance (Table 2).

**TABLE 2:** Predictive environmental variables with the highest percentage of contribution in the estimation of pine species distribution models.

Species	Predictive variables (percent of contribution in brackets)	% Acum.
*Pinus arizonica*	Bio6 (27.7), Bio8 (26.5), Bio17 (13.9), and Bio15 (7.7)	75.8
*P. brachyptera*	Bio11 (67.3) and Bio6 (10.4)	77.7
*P. cembroides*	Bio19 (44.6), Smin (17.9), and Bio1 (15.7)	78.2
*P. chihuahuana*	Bio15 (25.2), Smin (24.4), Bio19 (14.4), and Bio14 (12.6)	76.6
*P. cooperi*	Bio8 (55.1), Sstd (8.7). Bio18 (7.9), and Smin (5.2)	76.9
*P. devoniana*	Bio4 (50.9), Bio13 (22.2), and Bio9 (13.5)	86.6
*P. discolor*	Smean (47.9), Bio15 (14.5), and Bio11 (12.8)	75.2
*P. douglasiana*	Bio19 (37.2), Bio3 (23.8), and Bio7 (14.8)	75.8
*P. durangensis*	Bio19 (23.6), Elev (23.5), Bio8 (22.9), and Bio17 (19.1)	89.1
*P. engelmannii*	Bio19 (25.9), Elev (22.2), Bio15 (17.5), and Smin (12)	77.4
*P. herrerae*	Bio19 (53.6), Bio18 (12.6), Elev (7.8), and Bio3 (5.4)	79.4
*P. leiophylla*	Bio8 (28.8), Bio19 (21.8), Bio1 (14.1), Elev (7), and Smin (4.2)	75.9
*P. lumholtzii*	Bio16 (29.9), Bio19 (14.9), Elev (12.3), Bio3 (8.9), Bio17 (7.8)	73.8
*P. luzmariae*	Sstd (57.6), Bio6 (12.6), and Smean (7.5)	77.7
*P. maximinoi*	Bio18 (36.8), Bio19 (32.6), and Bio9 (6.9)	76.3
*P. oocarpa*	Bio16 (46.5), Vmean (18), and Bio3 (14.5)	79.0
*P. strobiformis*	Bio8 (30.8), Bio17 (18.4), Bio19 (14.5), and Elev (12.2)	75.9
*P. teocote*	Bio8 (50.4), Bio15 (13.7), Bio18 (10.6), and Sstd (6.6)	81.3
*P. yecorensis*	Bio17 (50.3), Smean (19.9), and Bio4 (10.7)	80.9

Among the species mostly influenced by temperature, *P. devoniana* occurs in sites with dominant seasonal temperature (Bio4) with good precipitation. *Pinus arizonica* is influenced mostly by the minimum temperature of the coldest month (Bio6) and the mean temperature of the wettest quarter (Bio8). For *P. brachyptera* , the mean temperature of the coldest quarter (Bio11) explained 67.3% of its distribution. In *P. cooperi* and *P. teocote*, the dominant variable was the mean temperature of the wettest quarter (Bio8).

The group of distributions influenced by precipitation includes *P. lumholtzii* and *P. oocarpa* , in which the precipitation of the wettest quarter (Bio16) contributed the highest percentage. Both species are also associated with another group of variables related to humidity, such as precipitation of the coldest quarter (Bio19) and water vapor pressure mean (Vmean).

For *P. cembroides* , the most important variable was the precipitation of the coldest quarter (Bio19), whereas for *P. herrerae* and *P. maximinoi* the two most important variables were rainfall in the warmest quarter (Bio18) and the coldest quarter (Bio19). Winter rains contributed mostly to define the distribution of *P. douglasiana* in the SMO, followed by temperature isothermality (Bio3). For *P. yecorensis*, the variable with the greatest contribution was precipitation of the driest quarter (Bio17), followed by mean solar radiation (Smean).

The group of species associated with solar radiation is formed by *P. discolor* and *P. luzmariae*, in which mean (smean) and standard solar radiation (sstd) contributed 47.9% and 57.6%, respectively.

The fourth group includes species that could be called generalists, corresponding to *P. chihuahuana* , *P. durangensis* , *P. engelmannii* , *P. leiophylla* , and *P. strobiformis* . In these species, the contribution of two or more variables to the models is proportional to each other. The distribution of *P. chihuahuana* was determined almost equally by both precipitation seasonality (Bio15) and minimum solar radiation (Smin). For *P. durangensis* and *P. engelmannii* , the main predictive variables were the precipitation of the coldest quarter (Bio19) and elevation (Elev), then mean temperature of the wettest quarter (Bio8) for the first species, and precipitation seasonality (Bio15) for the second. Finally, *P. leiophylla* and *P. strobiformis* were influenced by the mean temperature of the wettest quarter (Bio8), the precipitation of the coldest quarter (Bio19) and, to a lesser extent, by the elevation (Table 2).

#### 3.2.2 Distribution and Geographic Similarities

Maps were prepared to show the distribution of species over time (Figures S1.2–S1.20) and by individual period (Supporting Information 3). The species with the largest area of potential distribution are *P. cembroides* , *P. chihuahuana* , and *P. engelmannii* , covering more than 73,000 km^2^ each (Figures S3.3, S3.4, S3.10). In contrast, the species with the most reduced distribution areas were *P. brachyptera* (15,762 km^2^) and *P. yecorensis* (7738 km^2^) (Figures S3.2 and S3.19).

**FIGURE 1:**
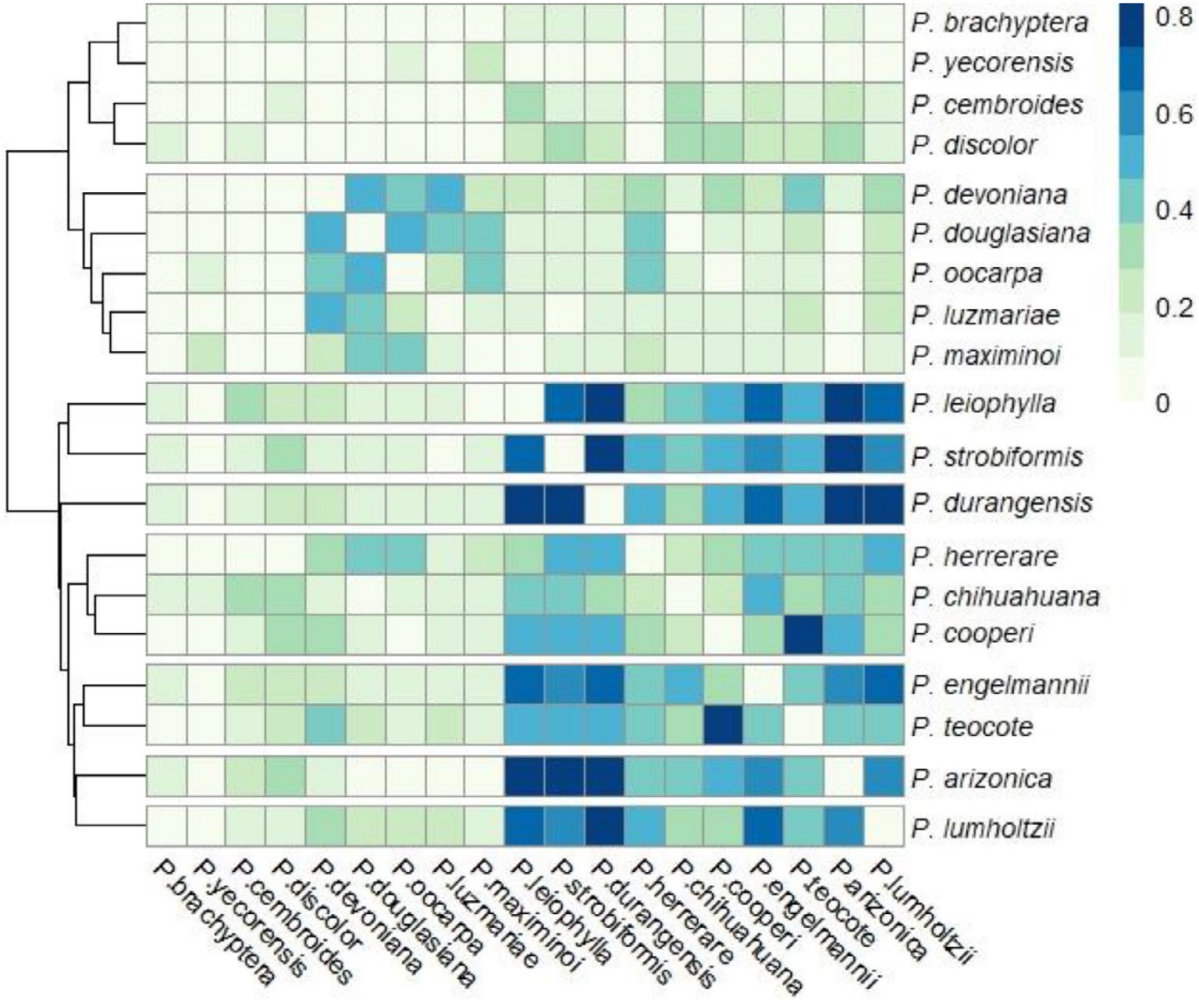
Heatmap and dendrogram of the pairwise similarity of the predicted geographic distributions among *Pinus* species quantified by the *D* index. Darker blue indicates higher similarity, and the hierarchical cluster on the left represents relationships between species based on these similarities.

The dendrogram of the *D* index similarity matrix shows several clustering branches (Figure 1). In the first cluster, *P. brachyptera* , which is distributed in the Madrean region, and *P. yecorensis* from Tropical Madrean have a small area of overlap in the northern zone of the SMO. This distribution appears related to that of the pinyons, which are distributed mainly on the eastern slopes but reach the north very close to the aforementioned species. In another group are the pines of tropical affinity: *P. devoniana*, *P. douglasiana*, *P. maximinoi*, *P. luzmariae*, and *P. oocarpa* . And in a larger group with very short branches are the species that have a wide distribution in the SMO and have the greatest affinity to the Madrean region.

Among the species found on the western slopes, *P. devoniana* and *P. luzmariae* limit their distribution to the north only as far as Durango; *P. douglasiana* reaches Chihuahua; and *P. herrerae* , *P. maximinoi*, and *P. oocarpa* get up to the Yécora region in central Sonora.

*Pinus arizonica* has a continuous distribution from Sonora to Durango, whereas *P. durangensis* and *P. engelmannii* extend south to Zacatecas near the border with Durango. *Pinus chihuahuana* , *P. leiophylla* , and *P. strobiformis* have a broad but fragmented distribution along the SMO.

Two species, *P. cooperi* and *P. teocote*, have a distribution restricted to the Madrean region in the central and southern zones of the SMO (Figures S1.6 and S1.19).

### 3.3 Species Distribution in Future Scenarios and Risk Level

The models show reductions in the potential distribution area for 18 of the 19 studied pine species. These results suggest that site suitability conditions will be reduced 97% for *P. brachyptera* by 2040 and will lead to its total disappearance by 2060 (Figure S3.2). Other species that will decrease, although less drastically, are *P. durangensis* (16%–22% between 2040 and 2100) (Figure S3.9), *P. devoniana* (22%–28%) (Figure S3.6), *P. cooperi* (23%–30%) (Figure S3.5), and *P. engelmannii* (25%–29%) (Figure S3.10).

Maximum reductions for species such as *P. cembroides* , *P. devoniana*, *P. discolor* , *P. maximinoi*, and *P. oocarpa* will be observed in 2080, followed by a slighter increase in 2100 (Figures S3.3, S3.6, S3.7, S3.15, and S3.16). The only species in which the total distribution area is foreseen unchanged by the end of the century is *P. yecorensis* (Figure S3.19). Some species such as *P. cembroides* , *P. devoniana*, and *P. oocarpa* show migration patterns to higher elevations (Figures S3.3, S3.6, and S3.16).

### 3.4 Hotspot Areas

The overlay of all the models for each period shows how the potential richness of *Pinus* varies spatially as a response to climate change scenarios. Although some areas show limiting conditions for pines, others show richness ranging from low (< 3 spp.), to typical (4–6 spp.) to high (7–9 spp.), with some exceptional areas potentially exceeding nine species (Figures S1.21–S1.25).

**FIGURE 2:**
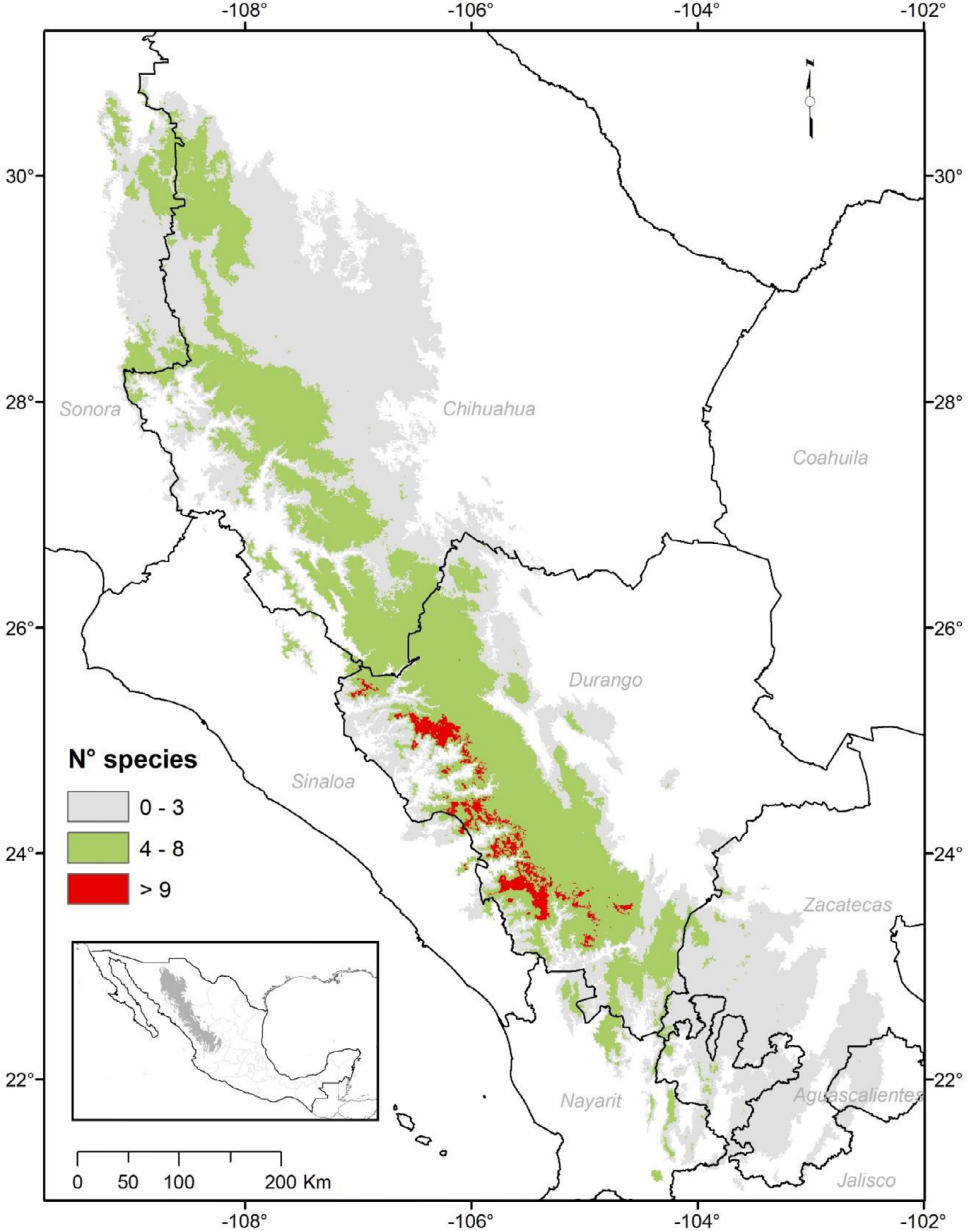
Current and future potential pine species distribution richness in Sierra Madre Occidental.

Figure 2 shows that a considerable proportion of the area is suitable for the distribution of multiple pine species in both the current and 2100 projections. It also identifies the hotspot areas where the highest number of species can be found (up to 9 spp.). The green areas represent the common pine diversity found in these forests (4–8 spp.). The highest number of pine species is found at the boundaries between the Madrean and Tropical Madrean ecoregions on the western slopes of the sierra in Durango.

**FIGURE 3:**
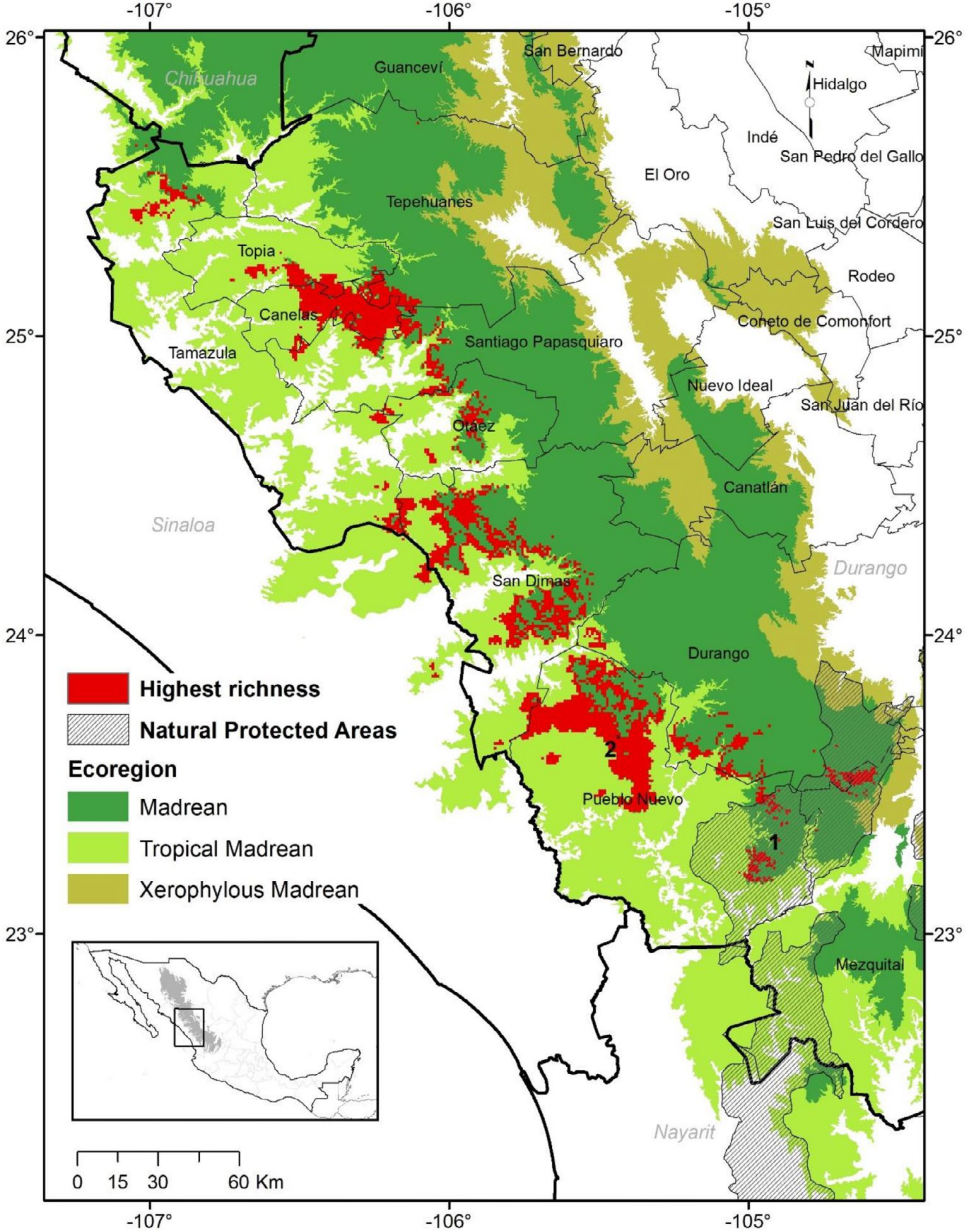
Areas with highest pine potential richness in the Sierra Madre Occidental. The numbers refer to the natural protected areas present in the area: (1) CADRN 043 Nayarit, and (2) Quebrada de Santa Bárbara.

Finally, only a small fraction of those potentially diverse areas is included within Natural Protected Areas (NPAs). One of them is “CADNR 043 Nayarit,” which includes small zones in the municipalities of Durango, Mezquital and Pueblo Nuevo. Another in Durango is the “Quebrada de Santa Bárbara,” also in southern Durango (Figure 3).

## 4 Discussion

### 4.1 General Findings in Environmental Variables

The models reflect the importance of temperature and precipitation for the presence of pines in the temperate forests of the SMO, especially during the driest period of the year, which occurs between March and June. Notably, the mean temperature of driest quarter (Bio9), the precipitation of the driest month (Bio14) and the precipitation of warmest quarter (Bio18) were key variables for most species. In addition, minimum solar radiation was found as an important variable for the *Pinus* group, especially in the early stages of growth (Foroughbakhch‐Pournavab et al. 2019).

### 4.2 Species Distribution Models

#### 4.2.1 Indicator Variables for Models

There are studies that were developed with predictor variables different from those reported here. For example, the models developed by Arreola‐Ramos (2016) for *P. devoniana* showed that the most important variables to predict its distribution were max temperature of warmest month (Bio5), mean temperature of wettest quarter (Bio8) and then Bio4. The differences may be caused by the number of records used while model development, since Arreola‐Ramos (2016) considered the total distribution of *P. devoniana* and included 292 records, almost six times more than the records used in this work. Nonetheless, the information presented here for the SMO contributes to the knowledge of the requirements of that species in its northernmost range.

For *P. cembroides* , the most important variable was the winter precipitation in our study, which reaches the SMO in the form of snow or sleet. Sáenz‐Ceja et al. (2022) found that the main variable for *P. cembroides* was elevation, which makes sense considering that at the national level this species is normally found at the lower parts of mountain ranges; however, at smaller scales like the SMO, the effect of elevation is likely not so relevant because there are records of this tree species also at locations with more than 3000 m asl, but always in the Xerophylous Madrean region.

Sáenz‐Ceja et al. (2022) also reported that the mean temperature of the coldest quarter (Bio11) was the most important variable for another pinyon, *P. discolor* (48.6%); however, in this work, it only contributed 12.8%. For *P. luzmariae*, the same authors report that the most important variables were elevation and (Bio12), variables that were eliminated in our study due to a low correlation. For *P. yecorensis*, they found that temperature seasonality (Bio4) and annual precipitation (Bio12) were the most important variables. These discrepancies may be attributable to the fact that Sáenz‐Ceja et al. (2022) conducted a principal component analysis (PCA) to identify the variables associated with the spatial distribution of the entire set of selected species. This approach may have resulted in the exclusion of specific conditions represented in a component of the PCA that was not included in the selection of the predictive variables and that are not representative for some taxa, such as the three species that have relatively restricted distributions (Greenacre et al. 2022).

The heterogeneity in the contribution and predictor variables in the models developed for the generalist species ( *P. chihuahuana* , *P. durangensis* , *P. engelmannii* , *P. leiophylla* , and *P. strobiformis* ) could be due in part to the wide distribution of those species throughout the SMO, as well as to the morphological and genetic variation they present in some areas (González‐Elizondo et al. 2012; Ávila‐Flores et al. 2016; Morales‐Nieto et al. 2021).

Some variables that influence the spatial distribution of certain species, such as soil depth, or the degree of slope and exposure, were not relevant in this work. These variables could give better results at a finer scale, as evidenced by Shirk et al. (2018). Due to the highly steep relief and the orographic complexity of the SMO, especially on the western slopes, the topographic variables at a scale of 1 km^2^ are sometimes somewhat biased, given that in a few tens of meters abrupt environmental shiftsoccur.

#### 4.2.2 Distributions and Affinities

The potential distribution areas obtained in this research are similar to those reported by Shirk et al. (2018) for *P. strobiformis* in the SMO and the southwestern United States, as well as those found by Sáenz‐Ceja et al. (2022) for *P. durangensis* and *P. yecorensis*, both endemics to the SMO. However, the same authors reported smaller distribution areas for *P. cembroides* , *P. discolor* , *P. leiophylla* , *P. luzmariae*, and *P. strobiformis* than those reported here. These results are contrary to the expected because their study included records with distribution in other mountainous ranges of Mexico.

On the other hand, there are models that estimate a larger distribution area for *P. engelmannii* predicting suitability in areas outside the natural distribution of the species, such as northcentral United States or Chile in South America (Jiménez‐Salazar and Méndez‐González 2021). These discrepancies may be due to the number of records included for modeling, the predictor variables (as well as their sources and scales) and the parameterization of the models in each analysis (Ahmadi et al. 2023).

The dendrogram of the similarity matrix provides valuable information on ecological affinities among pine species. For example, there is a relatively low probability (29%) that *P. leiophylla* (a species of humid and cold places) can be found with *P. cembroides* (of more arid environments); that co‐occurrence has been recorded for these species by Pompa‐García et al. (2023) but in that case, the name of *P. leiophylla* should refer to *P. chihuahuana* , which is at present recognized as a species (Gernandt and Pérez‐de la Rosa 2014). Both *P. cembroides* and *P. chihuahuana* usually occur together in sites sharing the same environmental characteristics. The probabilities of *P. leiophylla* encountering species from the Tropical Madrean region (those of warmer and more humid climates) are even lower (< 12%), but they are occasionally found in the same place (González‐Elizondo et al. 2012). Although low probabilities of mistake may occur, it is always recommended to verify the identity of species being worked on during field sampling.

### 4.3 Vulnerability due to Climatic Change

The increase in temperature and drought due to climate change has a negative impact on the future distribution of temperate climate tree species. Some projections indicate a reduction in the range of cold‐climate and high‐elevation species (Gutiérrez and Trejo 2014; Manzanilla‐Quiñones et al. 2019; Manzanilla‐Quijada 2021; Shirk et al. 2018). Others, instead, show an increase in their distribution area (Cruz‐Cárdenas et al. 2016; Romero‐Sánchez et al. 2018), probably because those with predicted expansion are naturally found in relatively warm regions, so the increase in temperatures may favor them. Specifically, Sáenz‐Romero et al. (2015) predict a decrease in *P. leiophylla* ; Shirk et al. (2018) for *P. strobiformis* ; and Antúnez et al. (2018) for *P. arizonica* , *P. cooperi* , *P. durangensis* , *P. engelmannii* , *P. leiophylla* , and *P. teocote* in the limits of Durango state.

These predictions represent a problem for forest conservation in the region, affecting future harvesting and environmental services. Although there are activities such as assisted migration that seek to place species in regions of better suitability, these types of actions should be developed with greater care, and their evaluation should consider socio‐cultural impacts, biodiversity, soil composition, as well as medium‐and long‐term monitoring (Seddon et al. 2021; Twardek et al. 2023). On the other hand, it is recommended to explore the genetic variation of individuals locally adapted to off‐average conditions and to test and reforest on the rear edge of their potential distribution (Arenas et al. 2021).

Although the models presented here predict varying degrees of reduction over time, they indicate that some of the pine species studied may potentially migrate to higher elevations where they can find the necessary conditions to continue developing. However, the rate at which this might naturally occur is uncertain, especially considering the current pace of climate change (Lenoir et al. 2008). On the other hand, warming and long periods of drought have been reported to impede the migration of some tree species (Minott and Kolb 2020). A worrying example is *P. brachyptera* , a tree that faces a high risk of disappearance due to its dependence on extremely low temperatures and its distribution, which is limited to a small area of the SMO, highlighting its vulnerability to rising temperatures.

There is evidence that warming increases plant water stress, which impacts plant growth, as well as biomass formation and reproductive structures, leading to low regeneration or survival rates (Marquardt et al. 2019; Badano et al. 2019). In the case of pines in western Mexico, water stress reduces latewood formation, and winter rains help mitigate the cessation of growth with the formation of early wood (González‐Cásares et al. 2020; Pompa‐García et al. 2021). Another study shows that *P. lumholtzii* is highly resistant to long periods of drought, but its recovery is very slow (Correa‐Díaz et al. 2023), which leaves these trees in vulnerable conditions to be affected by pests or diseases, which eventually leads to death (Sáenz‐Romero et al. 2023; Gómez‐Pineda et al. 2022).

However, rapid changes in climate could overcome the ability of plants to migrate or survive in the new environmental conditions (Sáenz‐Romero et al. 2016; Kijowska‐Oberc et al. 2020), especially when there are extraordinary events such as heat waves and extreme drought (Menezes‐Silva et al. 2019), which eventually lead to changes in the communities due to the empty niches of individuals or populations that do not survive (Kijowska‐Oberc et al. 2020).

### 4.4 Hotspots and Conservation

The diversity hotspots reported here show environmental conditions that are suitable for at least nine of the studied pine species for both the current and the more distant scenarios (2100). Most of these areas are located in the middle part of the SMO towards the western slopes, just where the Madrean (cooler climates) and Tropical Madrean regions converge, at elevations between 2100 and 2700 m asl. There is also a hotspot located in the Madrean region in southern Durango, right in front of the San Pedro‐Mezquital River basin, which works as an ecological corridor in which tropical, xerophytic, and temperate elements mix given its warm influence expanding throughout the middle basin (González‐Elizondo et al. 2007).

These sites match with the hotspot proposals of Sáenz‐Ceja et al. (2022) for pines that have been previously recognized for their great biological diversity, due to their endemism or the discovery of new plant species (Ávila‐González et al. 2022; González‐Gallegos et al. 2022; Castro‐Castro et al. 2023). These hotspots are also very important habitats for other biological groups such as birds (Kobelkowsky‐Vidrio et al. 2014; Remolina‐Figueroa et al. 2022), and coincide with the priority terrestrial regions proposed by Arriaga et al. (2000), as well as in two sites of extreme priority for conservation by CONABIO et al. (2007). Therefore, the results reported here only reaffirm the ecological importance of some areas of the SMO.

Despite these reports, these zones are poorly represented in only two NPA in the SMO. The first, “CADNR 043 Nayarit,” is an area that includes 23,290 km^2^ divided in portions of the states of Aguascalientes, Durango, Jalisco, Nayarit and Zacatecas. Approximately 218 km^2^ of the hotspot is located in this NPA, which represents barely 1% of its surface. A total of 21 pine species have been recorded there, including the endangered *P. maximartinezii* (SEMARNAT 2019). However, this NPA does not have a management plan neither a manager, and its occasional administration is divided between the North or West regional offices of the National Commission for Natural Protected Areas (CONANP) due to its multiple polygons and the large area that it occupies. Its lack of regulations and offices for physical presence have led it to be a vulnerable area that has a large number of mining concessions and several unregulated activities (Armendáriz‐Villegas et al. 2015; Jardel‐Peláez et al. 2017).

The second NPA, “Quebrada de Santa Bárbara” includes 590 ha of the core and buffer zone, which is completely within the hotspot obtained for pines. Five species of pines and nine species of other gymnosperms have been found in this area, in addition to being reported with a great richness of native and endemic plants (Noriega‐Villa et al. 2023). In this NPA, a Ejido (El Brillante) oversees monitoring and forest harvesting activities, ecotourism, and environmental education, as well as managing the recognition of its properties as High Conservation Value Forests by the Forest Stewardship Council (Heredia‐Telles et al. 2023).

There is scope for the creation of new NPAs to protect the diversity of temperate forests in the municipalities of Canelas (practically the entire upper part of the municipality is feasible), Pueblo Nuevo, San Dimas (with the proposed Piaxtla‐Tayoltita area; CONANP 2023), and Topia. However, one of the most common obstacles is the resistance of landowners and land users who prefer to manage their forest for timber harvesting or work in mines rather than open up to dialog and explore the possibility of setting aside areas for biodiversity conservation and low‐impact economic activities (Merino‐Pérez 2019; Hensler and Merçon 2020).

We agree with Sáenz‐Ceja et al. (2022) and López‐González et al. (2023) that an evaluation of conservation priorities in SMO with updated information and prospective on future socio‐environmental situations is imperative. It is necessary for the State to regulate the activities, programs, and jurisdiction of dependencies already working within the current NPAs to avoid possible overlapping of activities (Armendáriz‐Villegas et al. 2015; Merino‐Pérez 2019).

In addition, it is necessary to socialize the different NPA categories and to clarify that there are socio‐ecological models that consider human settlements and sustainable use activities within forests under conservation, as well as alternative economic activities that favor biodiversity (Galicia et al. 2018). Successful conservation requires a holistic approach that integrates the socioeconomic aspects of forest owners, along with ecological and environmental aspects. This approach not only benefits biodiversity or ecosystems but also generates welfare for the social sector that depends on forest services.

## Author Contributions

**Lizeth Ruacho‐González:** conceptualization (lead), data curation (lead), formal analysis (lead), methodology (lead), writing – original draft (lead). **José Javier Corral‐Rivas:** formal analysis (supporting), funding acquisition (lead), investigation (equal), resources (equal), supervision (equal), validation (equal), writing – review and editing (equal). **Jesús Guadalupe González‐Gallegos:** formal analysis (supporting), funding acquisition (supporting), methodology (supporting), resources (equal), supervision (equal), validation (equal), writing – review and editing (equal). **M. Socorro González‐Elizondo:** data curation (equal), funding acquisition (lead), investigation (equal), methodology (supporting), resources (equal), validation (equal), writing – review and editing (supporting). **Pablito Marcelo López‐Serrano:** formal analysis (supporting), methodology (supporting), writing – review and editing (supporting). **Jaime Briseño‐Reyes:** formal analysis (supporting), methodology (supporting), writing – review and editing (supporting).

## Conflicts of Interest

The authors declare no conflicts of interest.

## Supporting information


Appendix S1.

## Data Availability

The data that support the findings of this study are openly available in Dryad at https://doi.org/10.5061/dryad.p8cz8wb06.
