# Supplementary material for: Diversity Hotspots and Vulnerability of Pine Species in the Sierra Madre Occidental, Western Mexico
Source: Ecol Evol. 2025 Jul 9;15(7):e71743. doi: 10.1002/ece3.71743 (PMC12240594; doi:10.1002/ece3.71743)
Supplement: Supplementary file 1 — Appendix S1. [file ECE3-15-e71743-s001.zip › ece371743-sup-0001-supinfo.docx]

**Supporting Information 3 for**

**Diversity hotspots and vulnerability of pine species in the Sierra Madre Occidental, western Mexico**

Lizeth Ruacho-González^1,2^, José Javier Corral-Rivas*^3^, Jesús Guadalupe González-Gallegos^2^, M. Socorro González-Elizondo^2^, Pablito Marcelo López-Serrano^4^, Jaime Briseño-Reyes^3^

^1^ Programa Institucional de Doctorado en Ciencias Agropecuarias y Forestales, Universidad Juárez del Estado de Durango, Durango, México.

^2^ Centro Interdisciplinario de Investigación para el Desarrollo Integral Regional Unidad Durango, Instituto Politécnico Nacional, Durango, México

^3^ Facultad de Ciencias Forestales y Ambientales, Universidad Juárez del Estado de Durango, Durango, México

^4^ Instituto de Silvicultura e Industria de la Madera, Universidad Juárez del Estado de Durango, Durango, México.

* Corresponding author: [jcorral@ujed.mx](mailto:jcorral@ujed.mx)

**CONTENTS**:

Species current (A) and future (B-E) individual distribution models. The graph (F) illustrates the approximate area occupied by the potential distribution of the species in each altitudinal interval in the Sierra Madre Occidental in the current scenario (dark green) and in the 2100 scenario (dark orange).

[Figure S3.1. *Pinus arizonica* 3](#_Toc193728243)

[Figure S3.2. *Pinus brachyptera* 4](#_Toc193728244)

[Figure S3.3. *Pinus cembroides* 5](#_Toc193728245)

[Figure S3.4*. Pinus chihuahuana* 6](#_Toc193728246)

[Figure S3.5. *Pinus cooperi* 7](#_Toc193728247)

[Figure S3.6. *Pinus devoniana* 8](#_Toc193728248)

[Figure S3.7*. Pinus discolor* 9](#_Toc193728249)

[Figure S3.8. *Pinus douglasiana* 10](#_Toc193728250)

[Figure S3.9. *Pinus durangensis* 11](#_Toc193728251)

[Figure S3.10. *Pinus emgelmannii* 12](#_Toc193728252)

[Figure S3.11. *Pinus herrerae* 13](#_Toc193728253)

[Figure S3.12. *Pinus leiophylla* 14](#_Toc193728254)

[Figure S3.13. *Pinus lumholtzii* 15](#_Toc193728255)

[Figure S3.14. *Pinus luzmariae* 16](#_Toc193728256)

[Figure S3.15. *Pinus maximinoi* 17](#_Toc193728257)

[Figure S3.16. *Pinus oocarpa* 18](#_Toc193728258)

[Figure S3.17. *Pinus strobiformis* 19](#_Toc193728259)

[Figure S3.18. *Pinus teocote* 20](#_Toc193728260)

[Figure S3.19. *Pinus yecorensis* 21](#_Toc193728261)

| A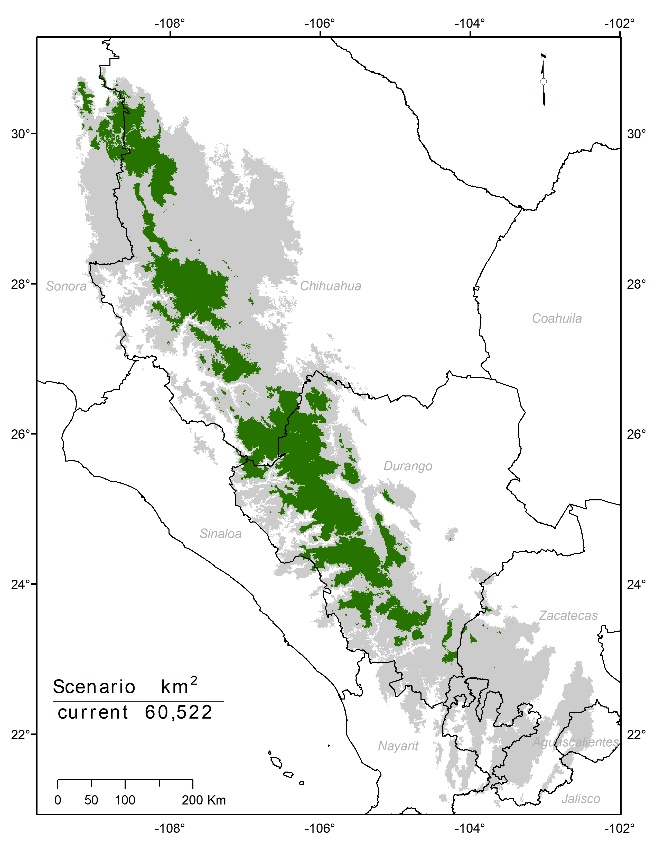 | B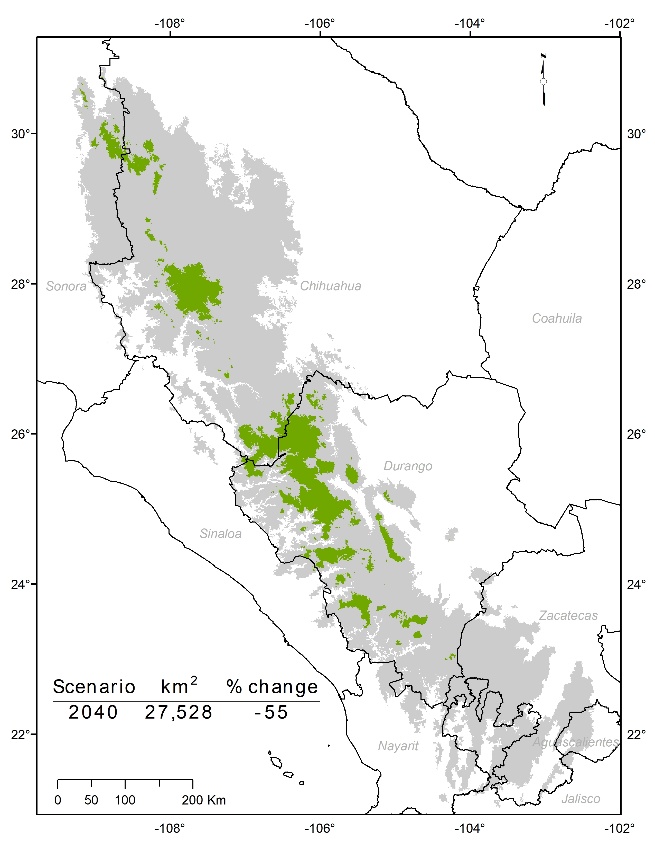 |
| --- | --- |
| C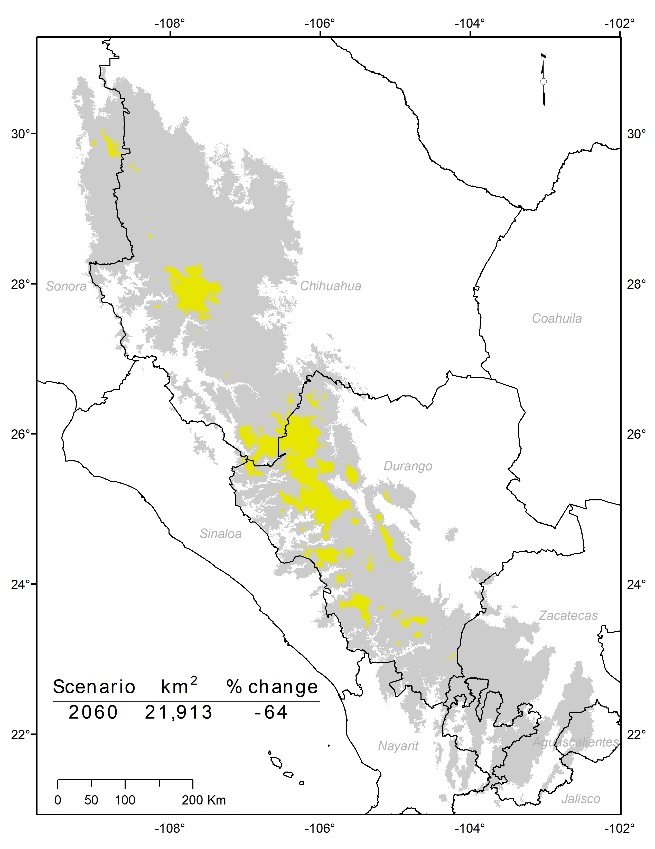 | D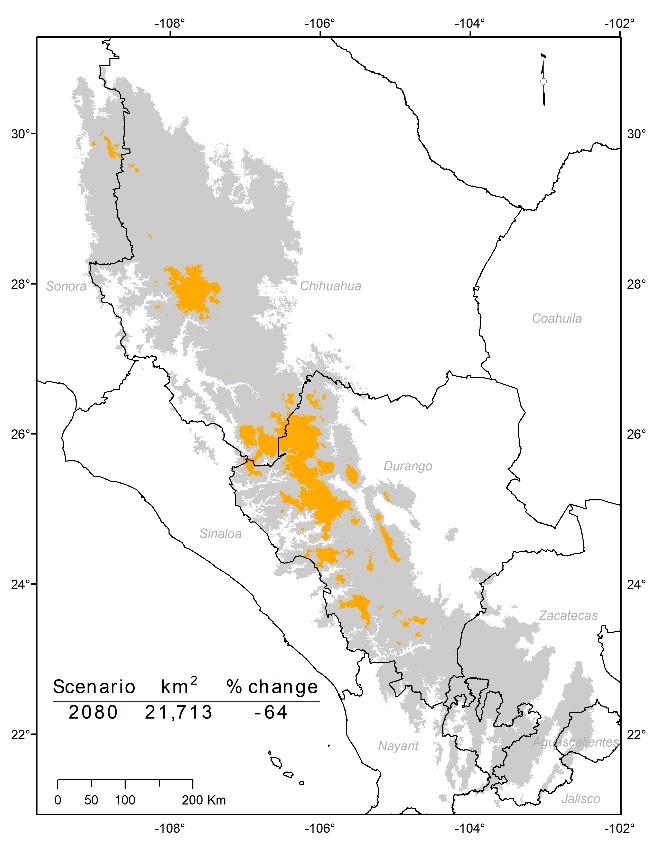 |
| E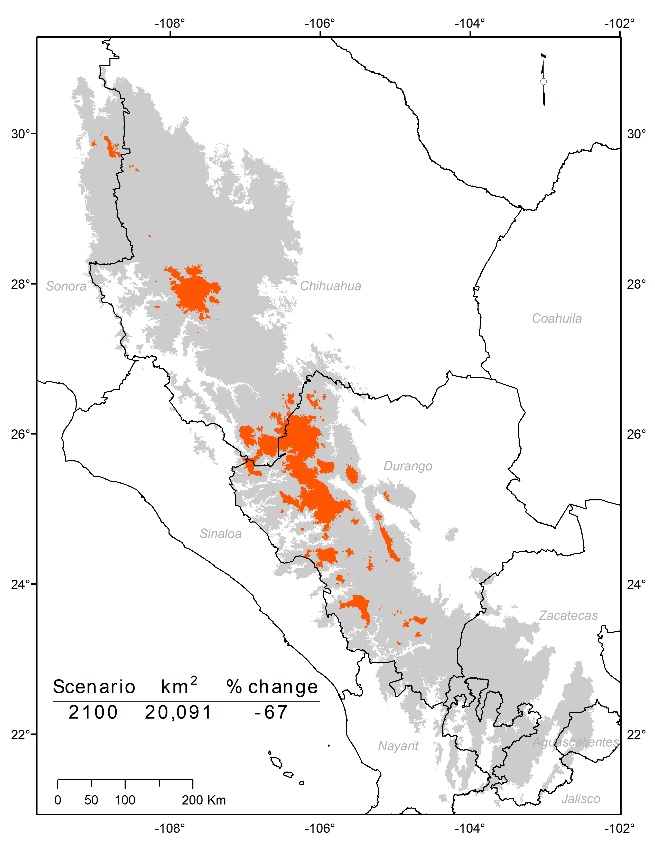 | F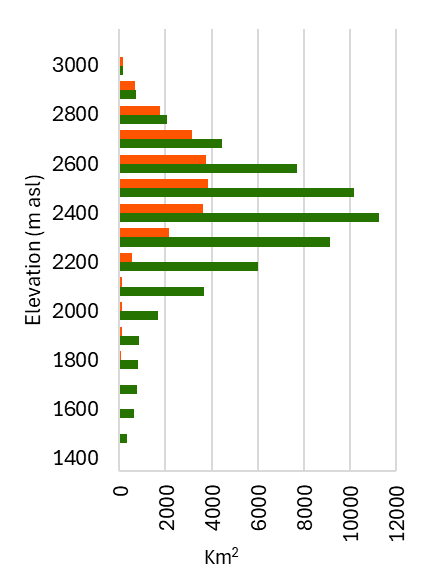 |

Figure S3.1. *Pinus arizonica* current (A) and future (B-E) distribution models. The graph (F) illustrates the approximate area occupied by the potential distribution of the species in each altitudinal interval in the Sierra Madre Occidental in the current scenario (dark green) and in the 2100 scenario (dark orange).

| A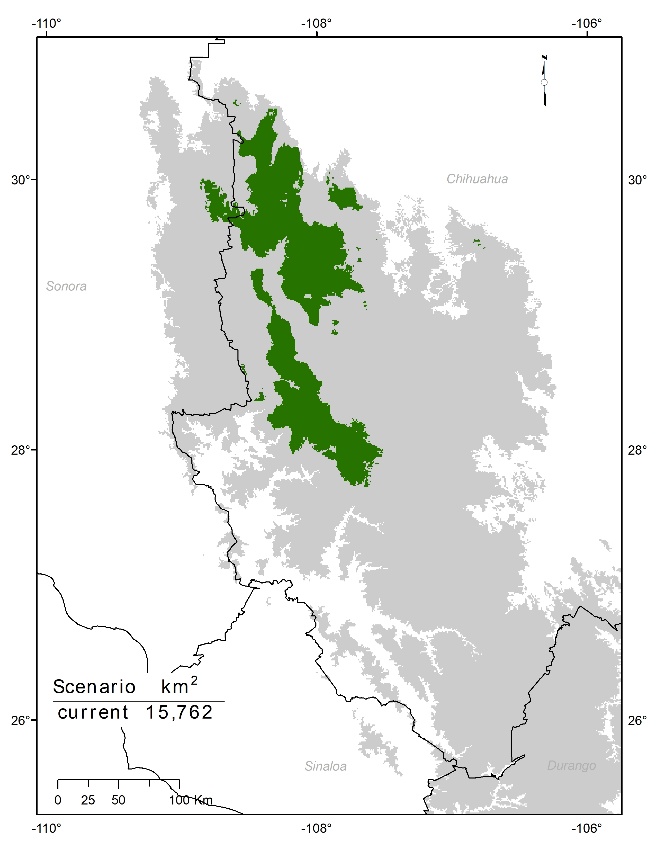 | B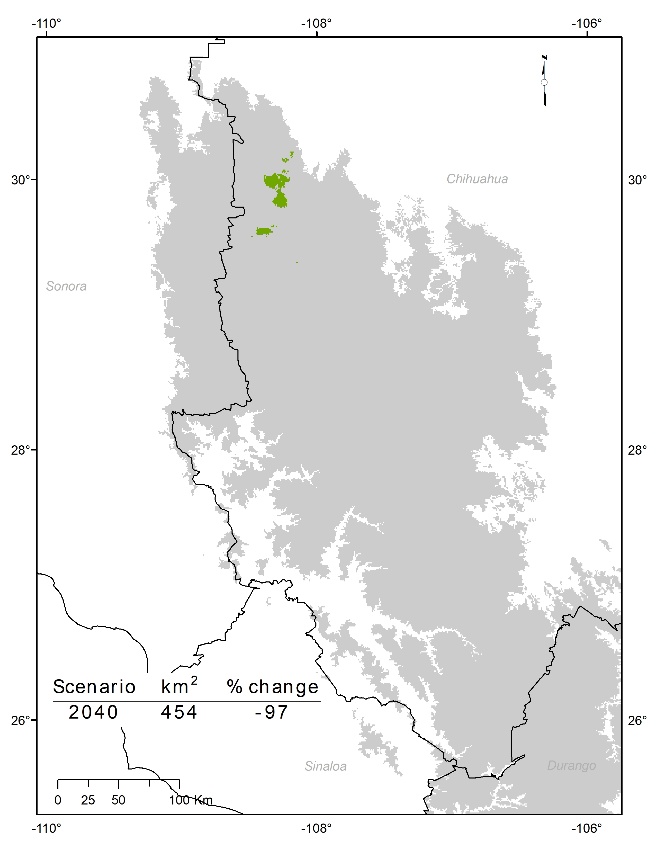 |
| --- | --- |
| C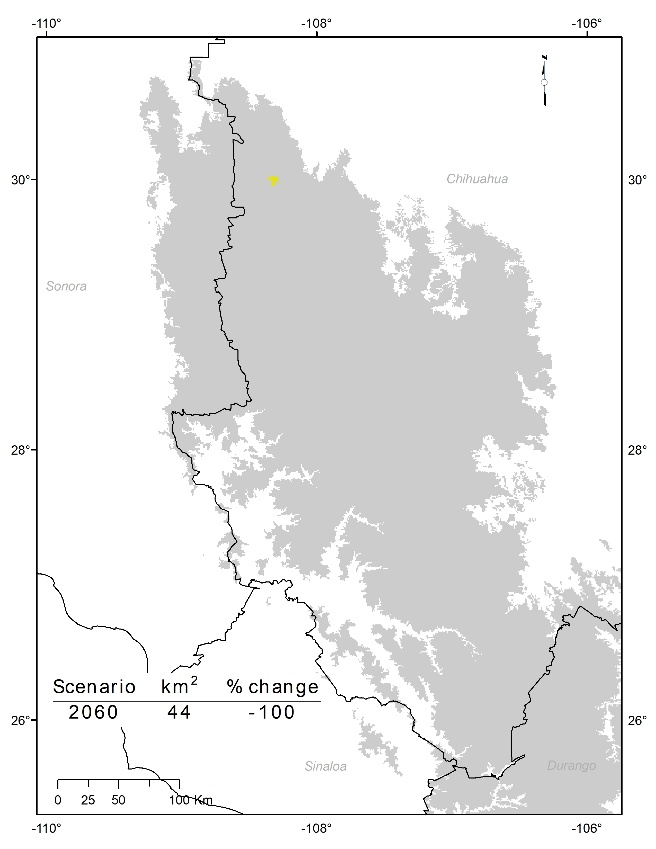 | D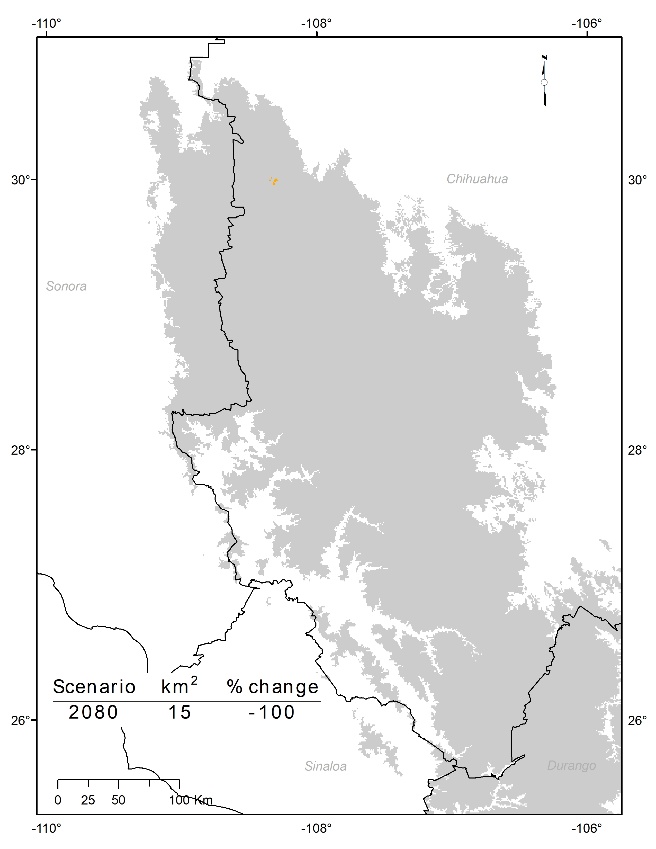 |
| E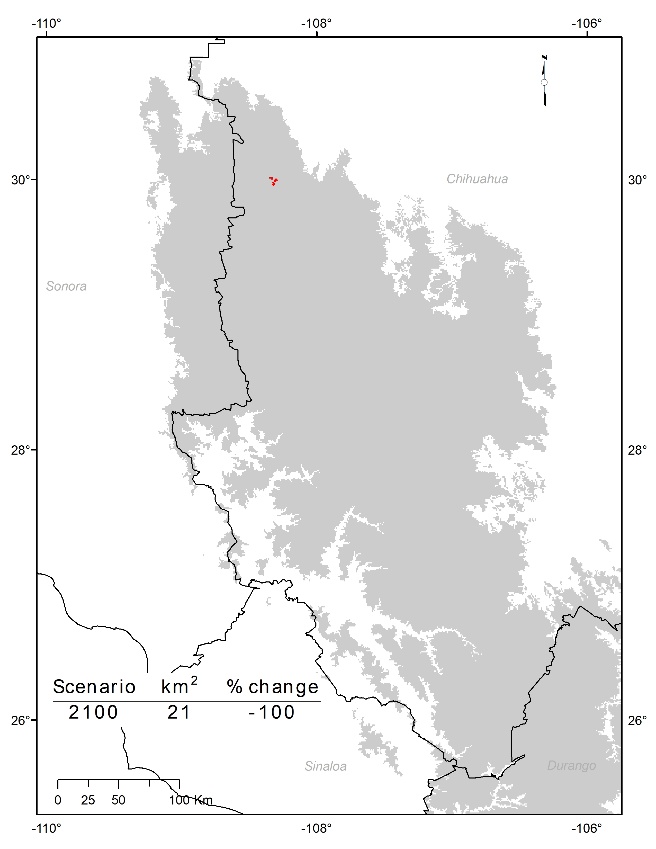 | F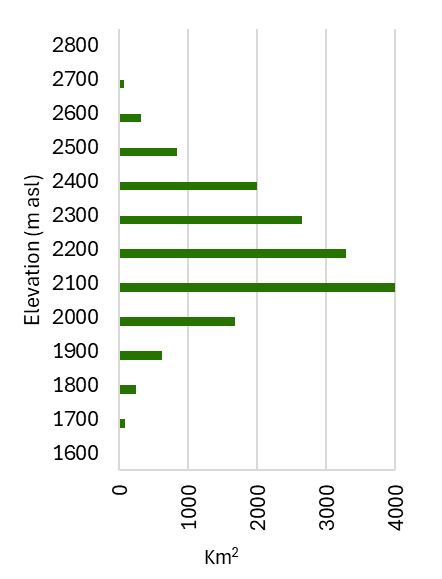 |

Figure S3.2. *Pinus brachyptera* current (A) and future (B-E) distribution models. The graph (F) illustrates the approximate area occupied by the potential distribution of the species in each altitudinal interval in the Sierra Madre Occidental in the current scenario (dark green) and in the 2100 scenario (dark orange).

| A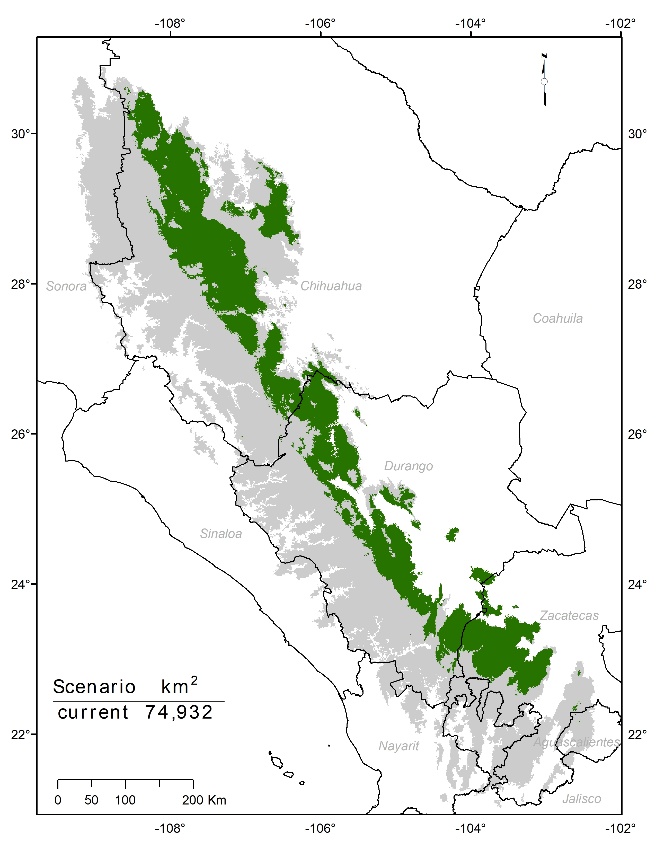 | B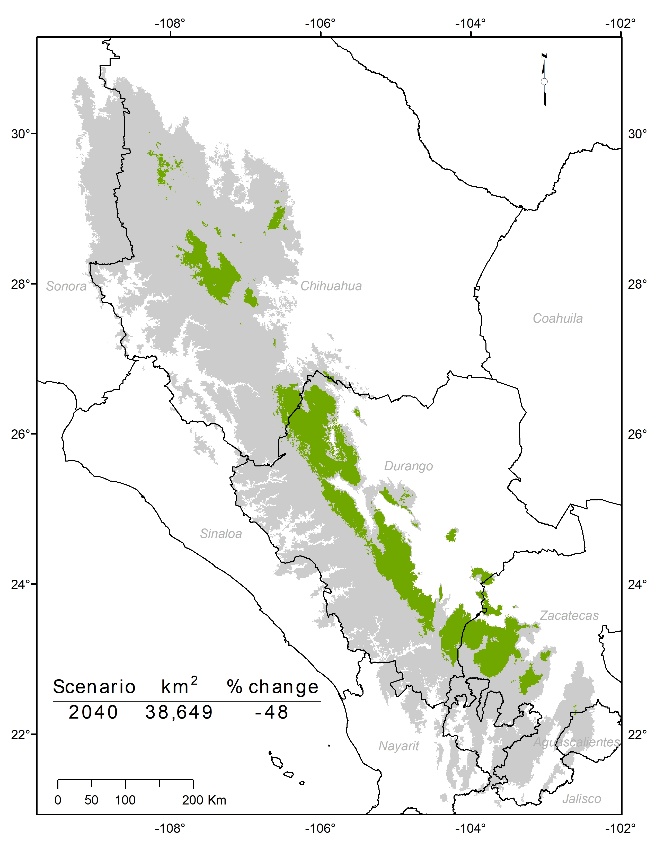 |
| --- | --- |
| C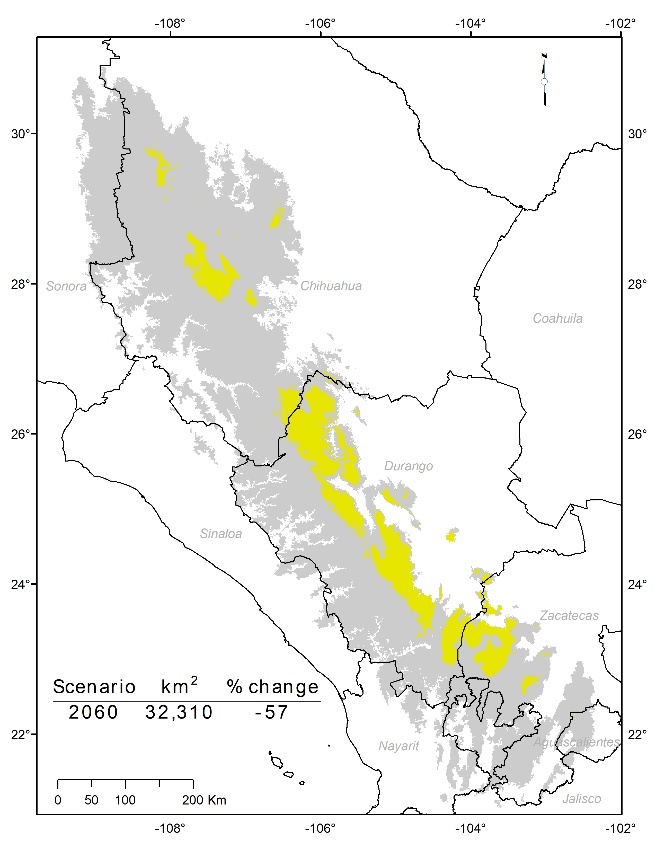 | D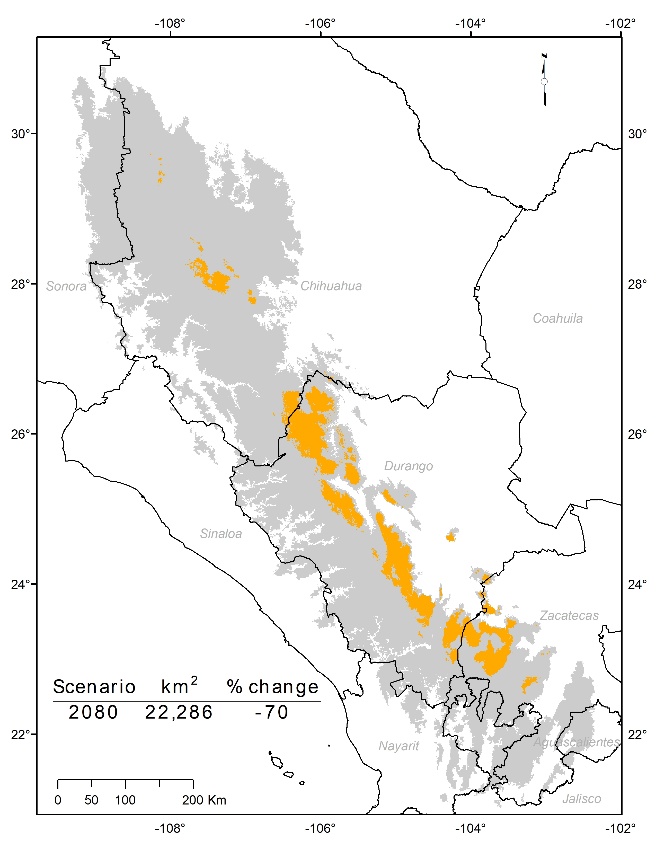 |
| E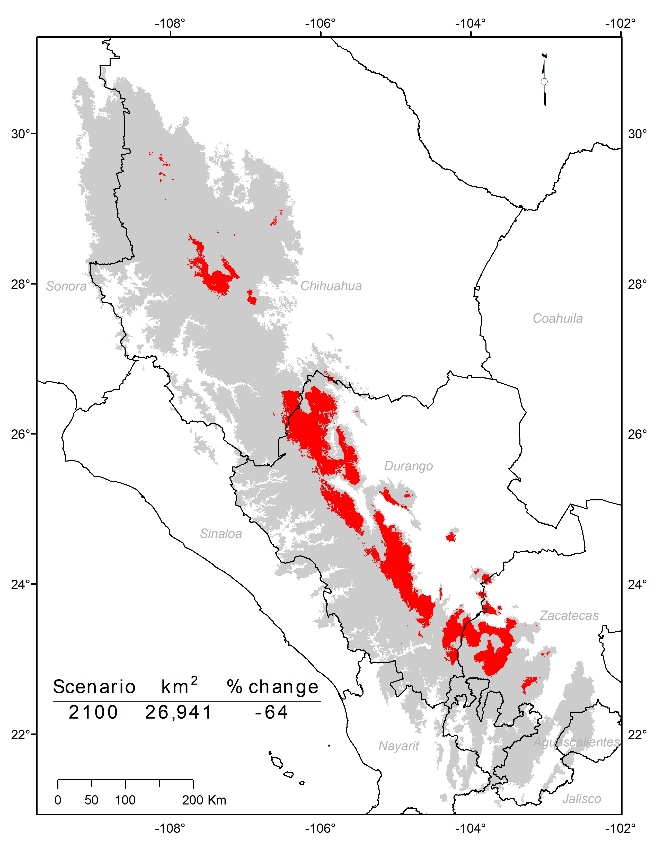 | F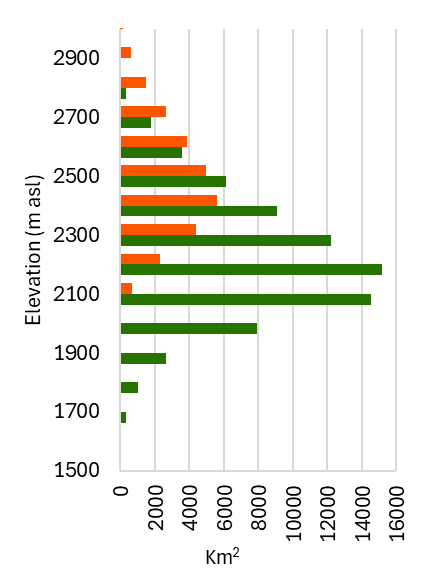 |

Figure S3.3. *Pinus cembroides* current (A) and future (B-E) distribution models. The graph (F) illustrates the approximate area occupied by the potential distribution of the species in each altitudinal interval in the Sierra Madre Occidental in the current scenario (dark green) and in the 2100 scenario (dark orange).

| A 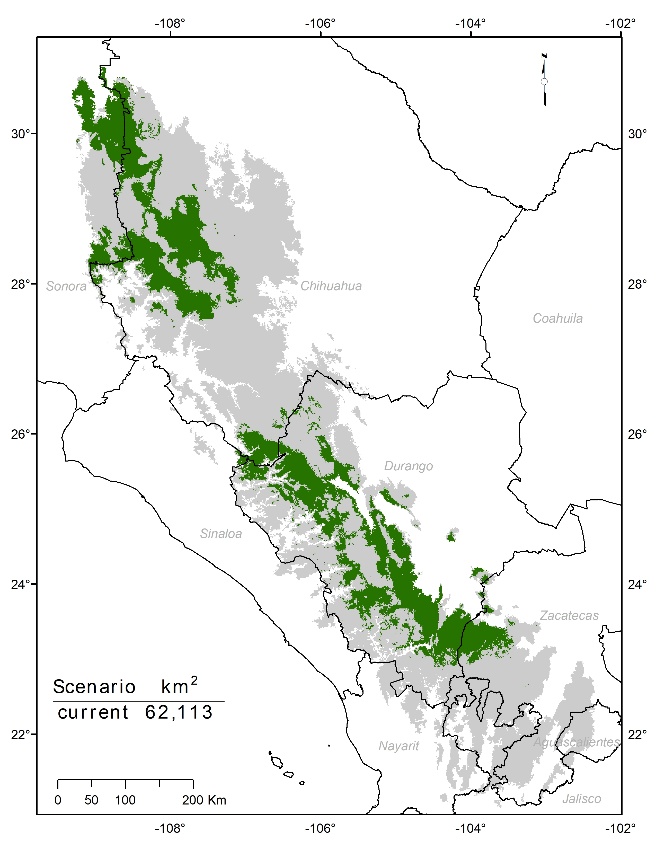 | B 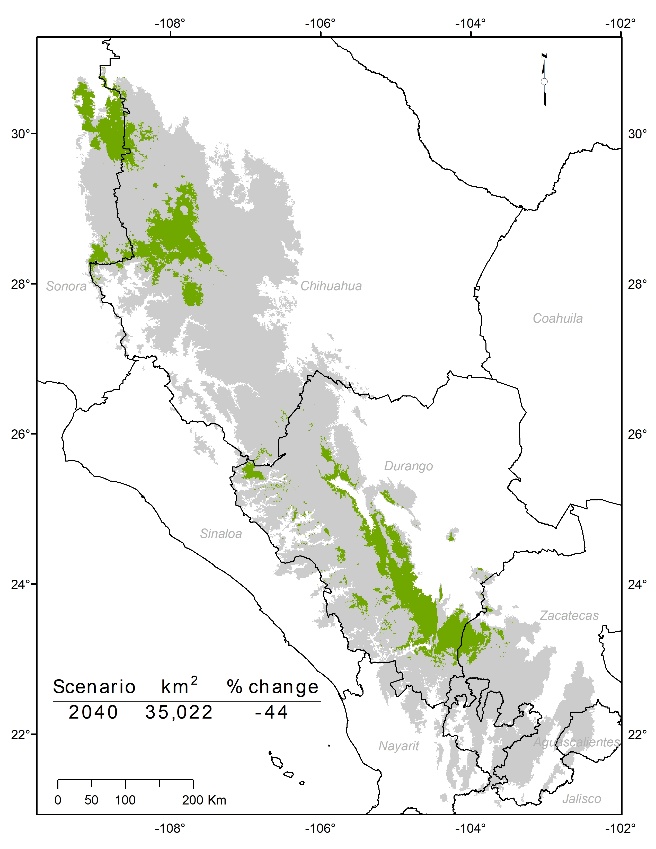 |
| --- | --- |
| C 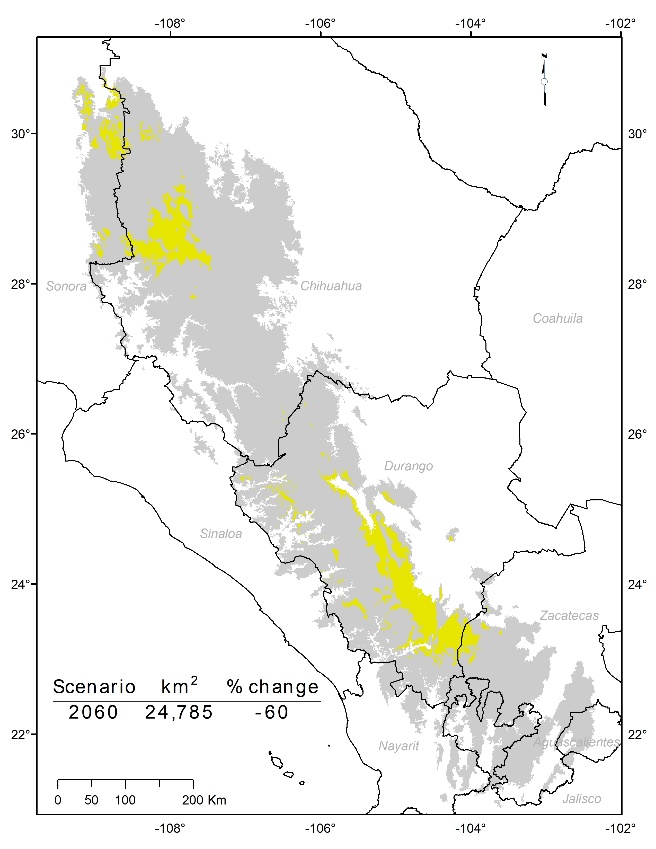 | D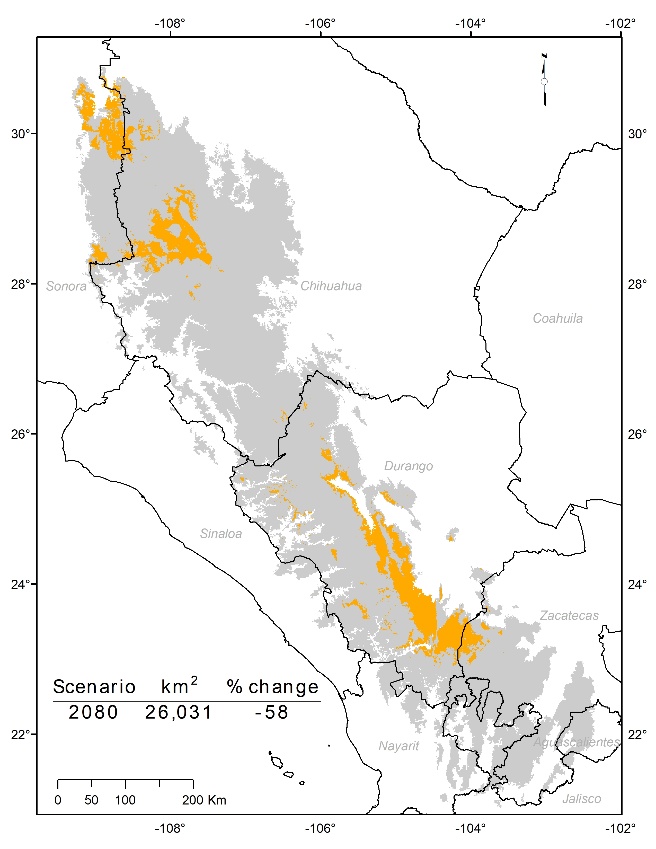 |
| E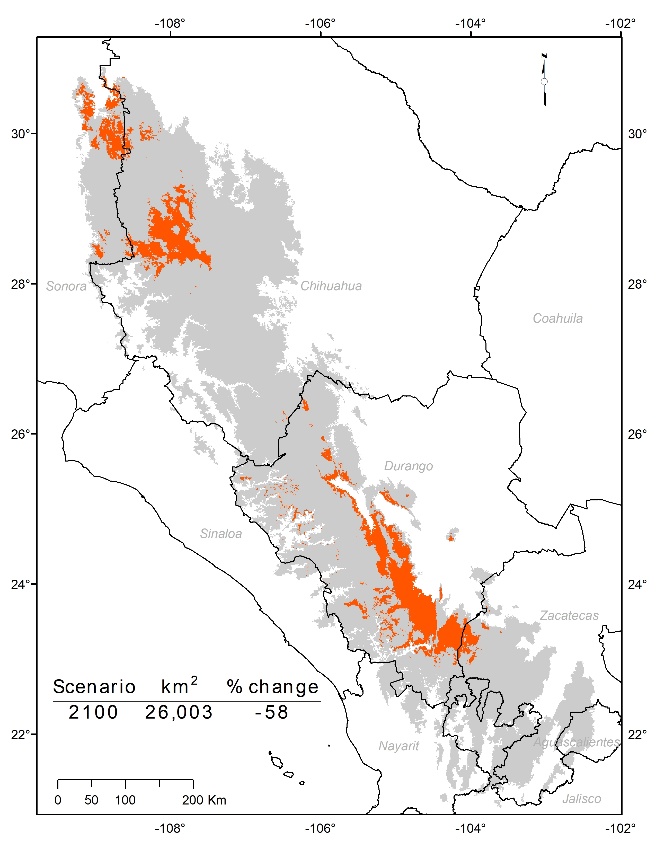 | F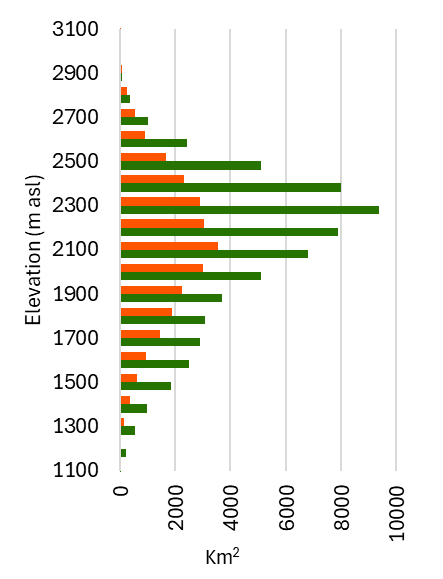 |

Figure S3.4*. Pinus chihuahuana* current (A) and future (B-E) distribution models. The graph (F) illustrates the approximate area occupied by the potential distribution of the species in each altitudinal interval in the Sierra Madre Occidental in the current scenario (dark green) and in the 2100 scenario (dark orange).

| A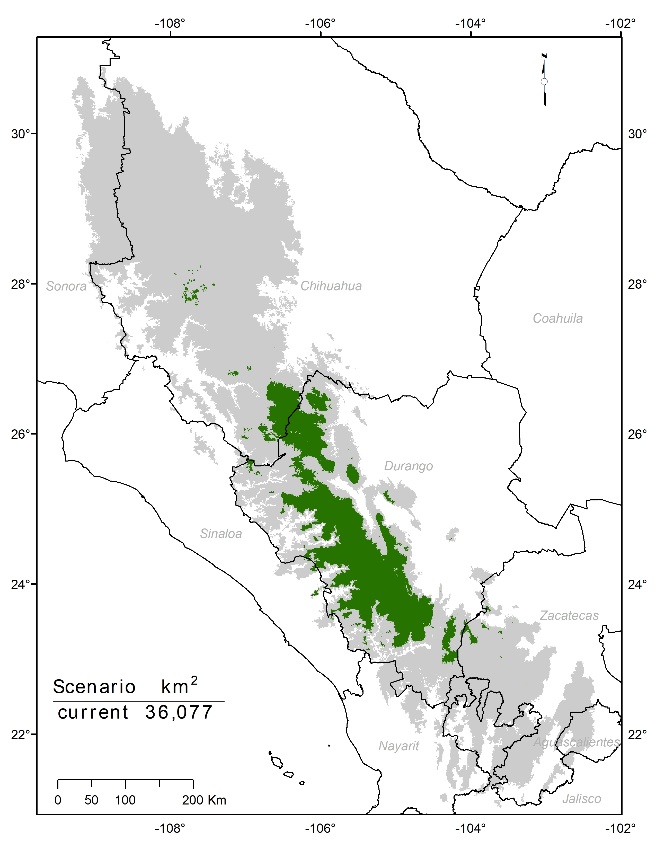 | B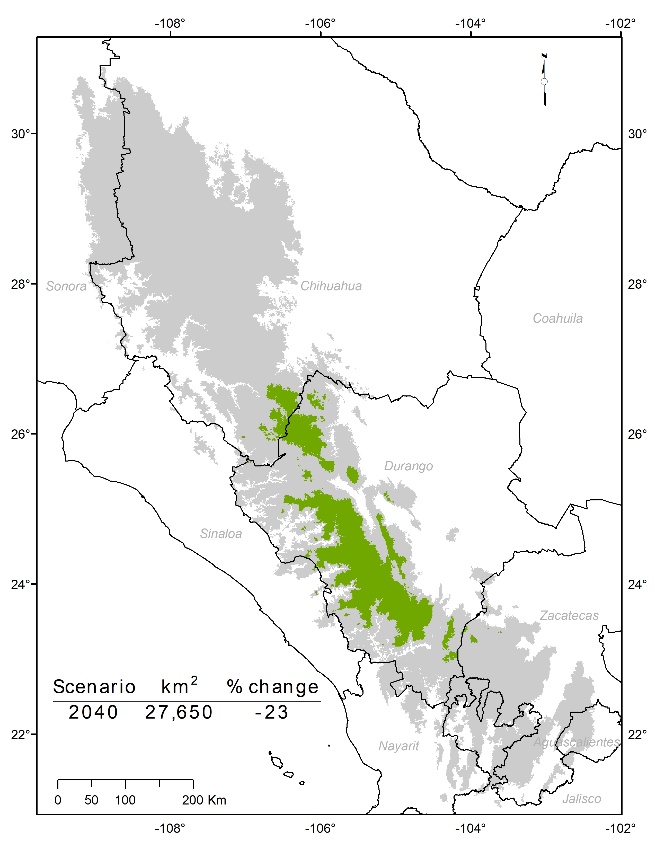 |
| --- | --- |
| C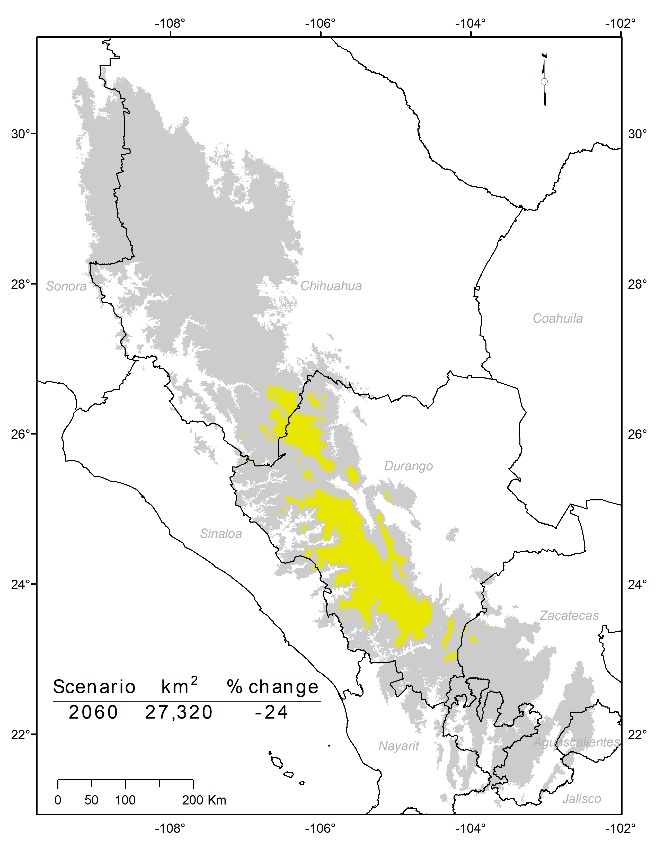 | D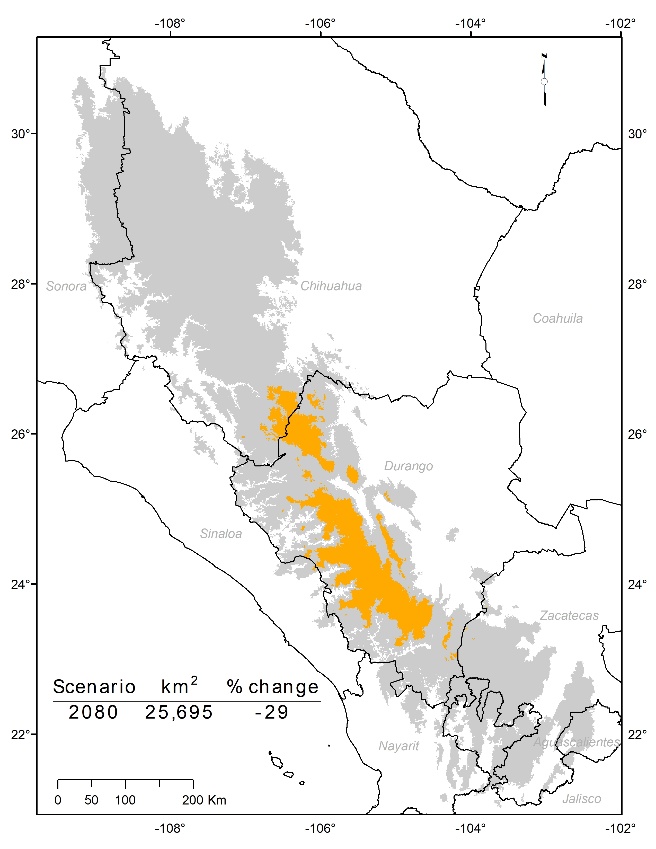 |
| E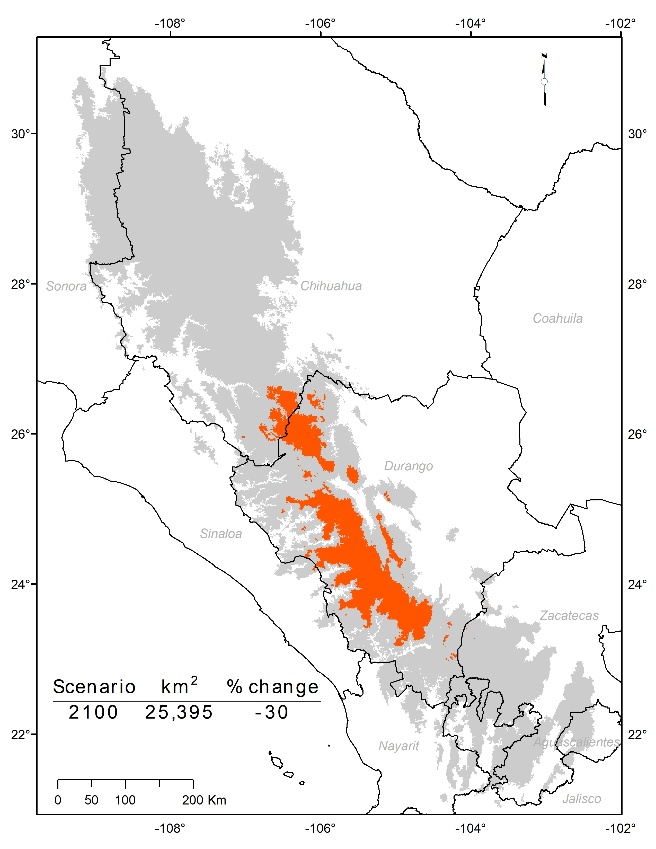 | F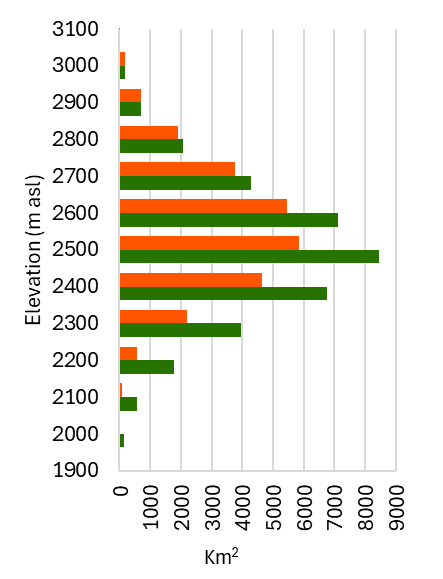 |

Figure S3.5. *Pinus cooperi* current (A) and future (B-E) distribution models. The graph (F) illustrates the approximate area occupied by the potential distribution of the species in each altitudinal interval in the Sierra Madre Occidental in the current scenario (dark green) and in the 2100 scenario (dark orange).

| A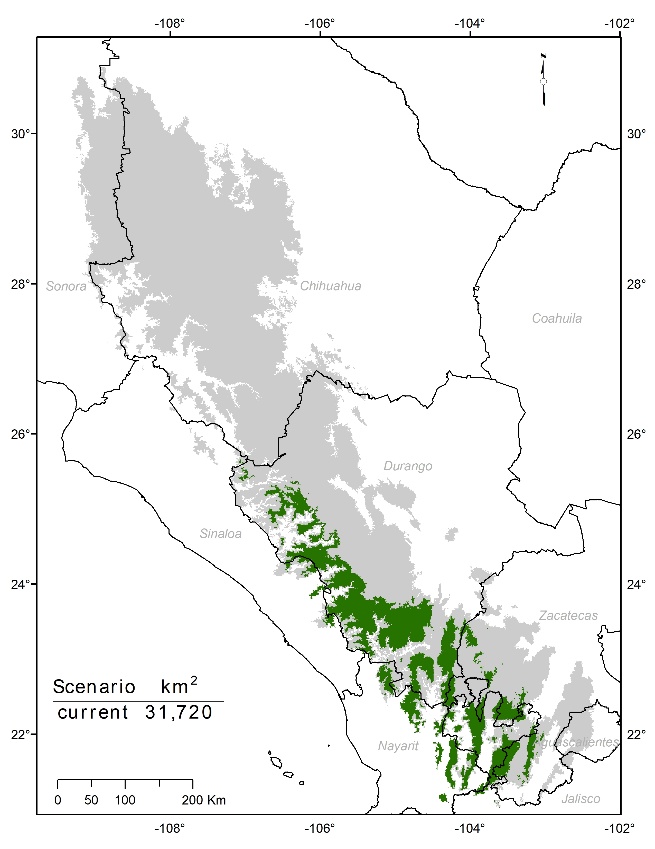 | B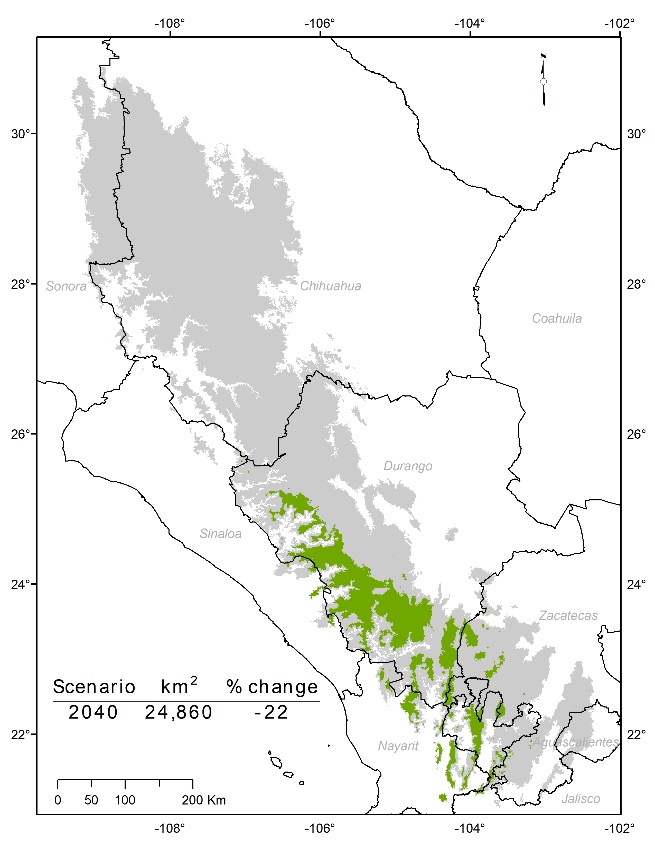 |
| --- | --- |
| C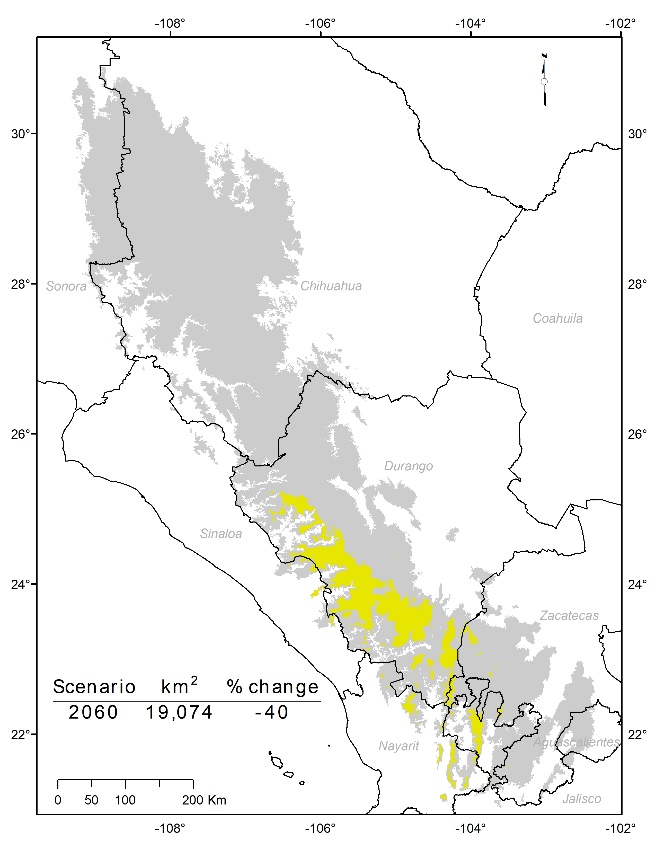 | D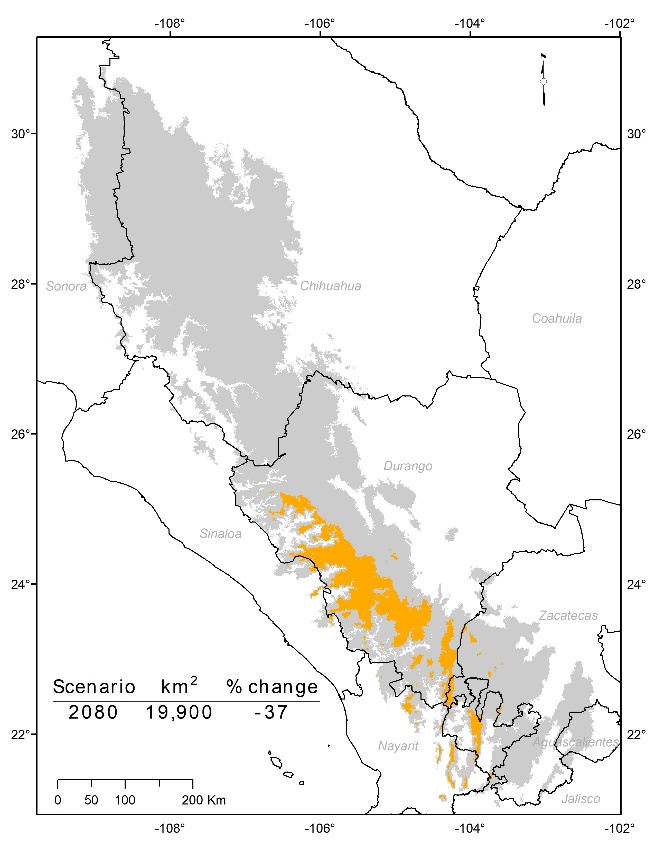 |
| E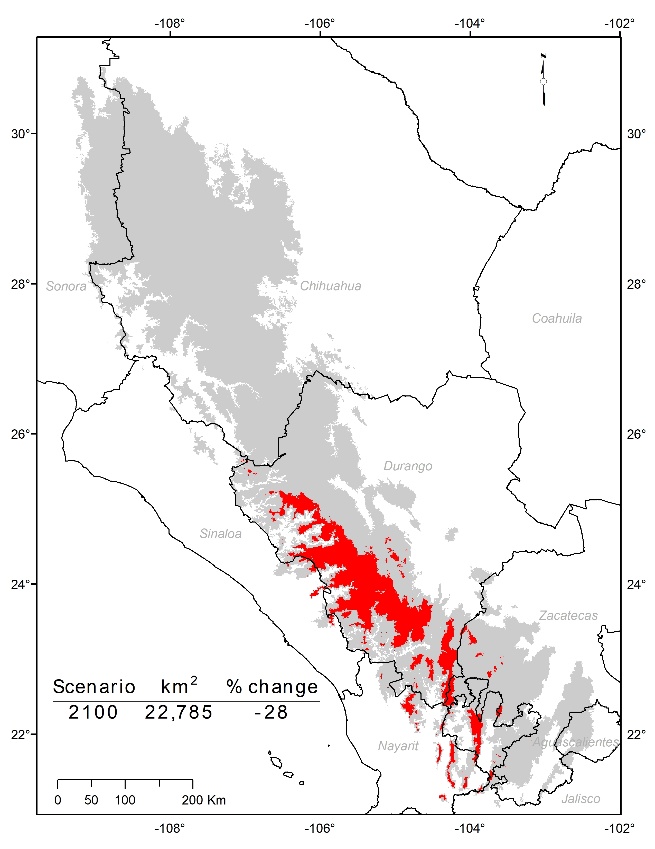 | F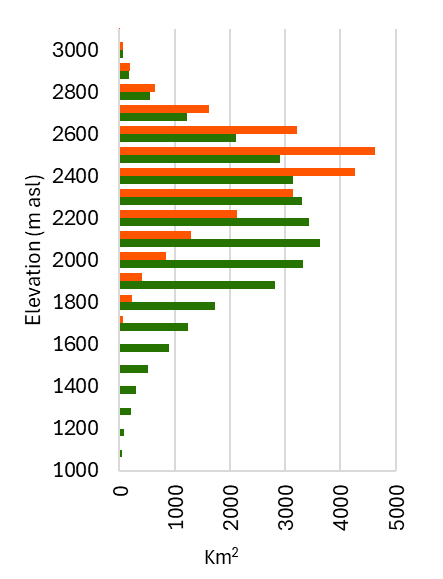 |

Figure S3.6. *Pinus devoniana* current (A) and future (B-E) distribution models. The graph (F) illustrates the approximate area occupied by the potential distribution of the species in each altitudinal interval in the Sierra Madre Occidental in the current scenario (dark green) and in the 2100 scenario (dark orange).

| A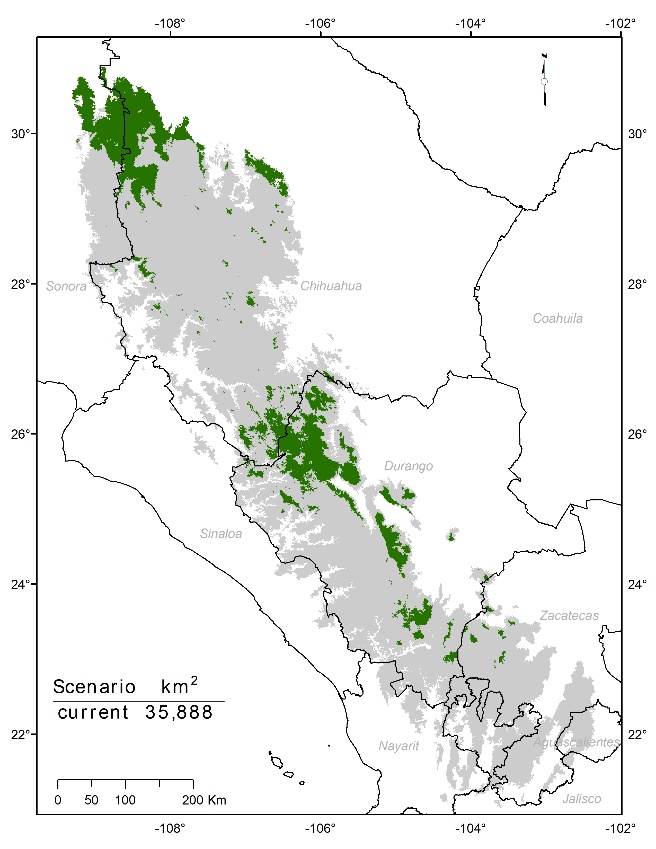 | B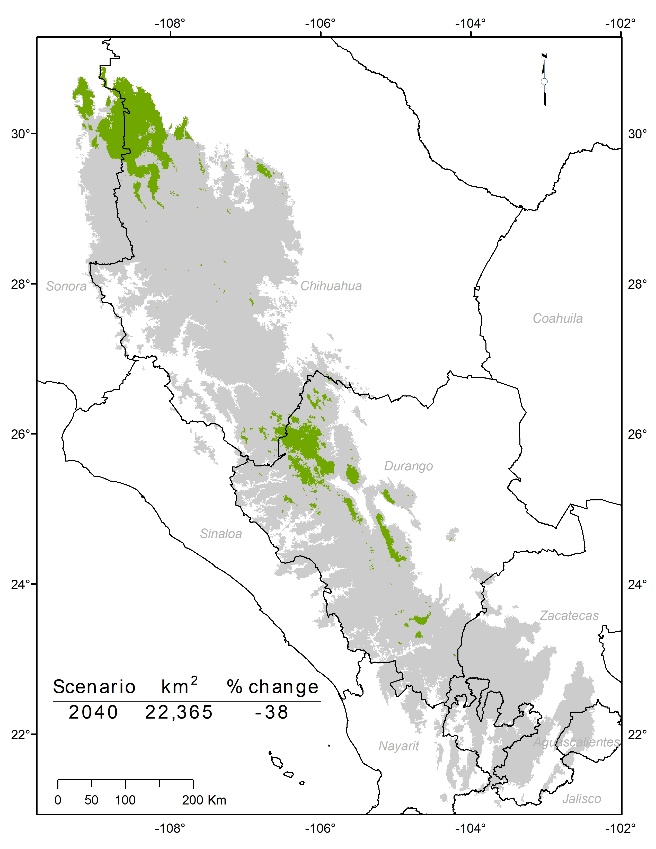 |
| --- | --- |
| C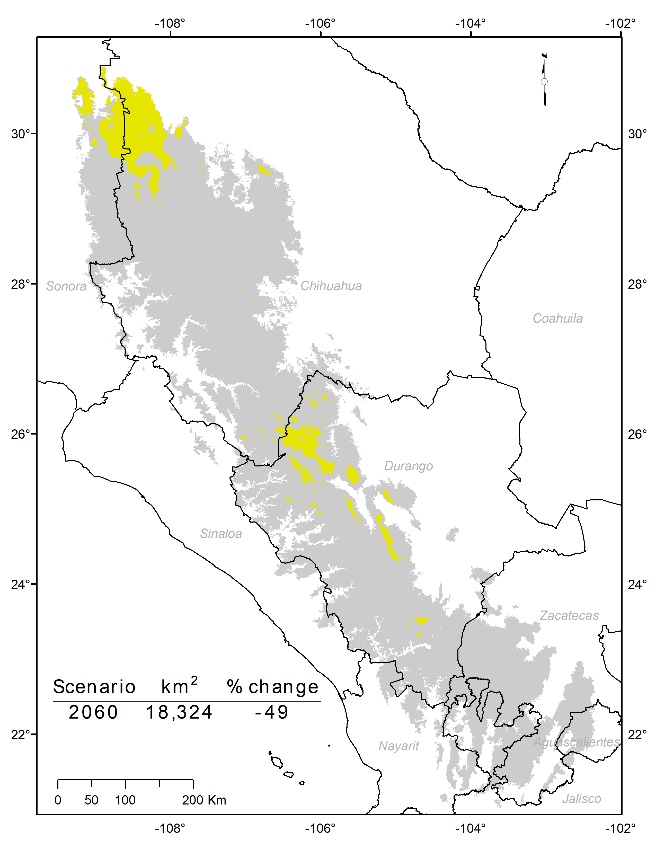 | D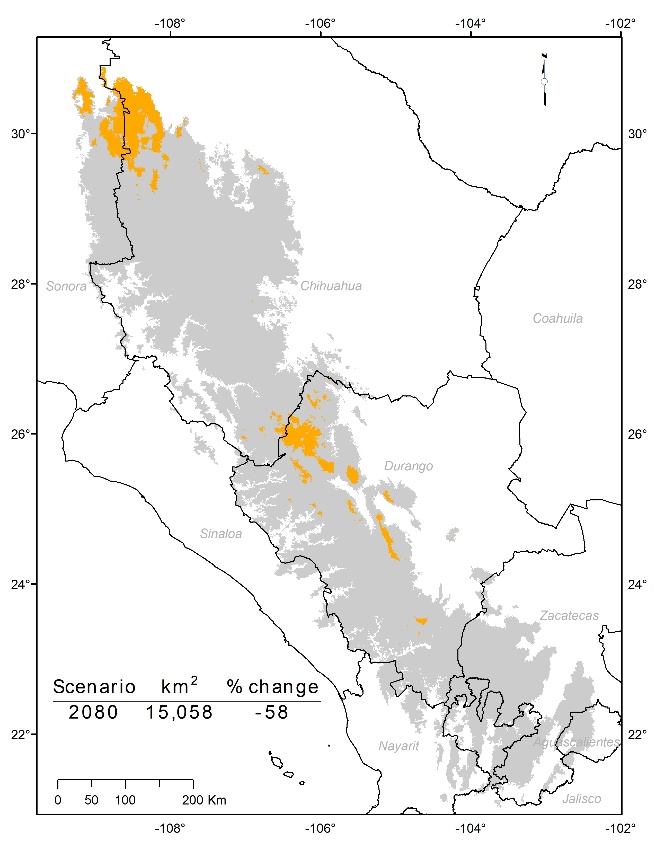 |
| E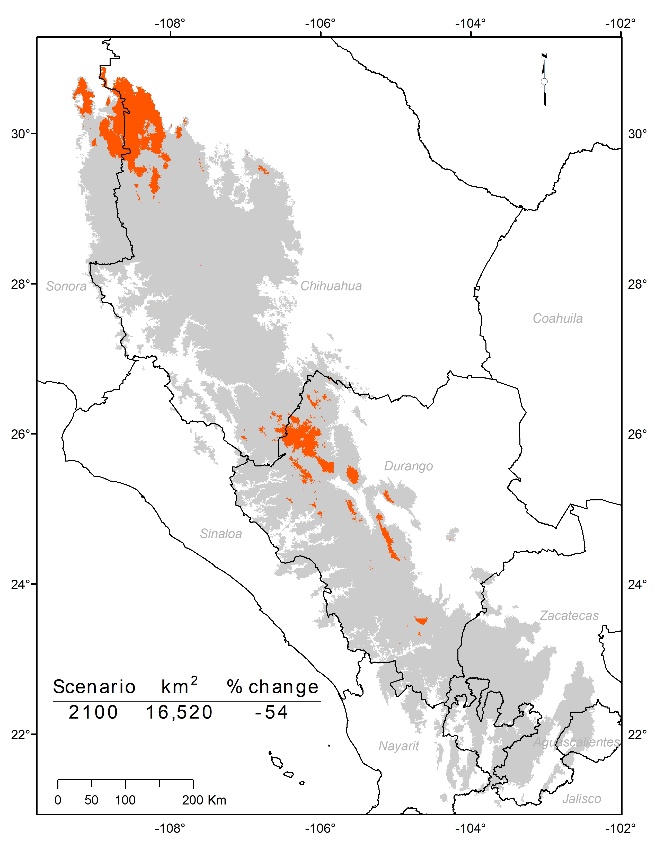 | F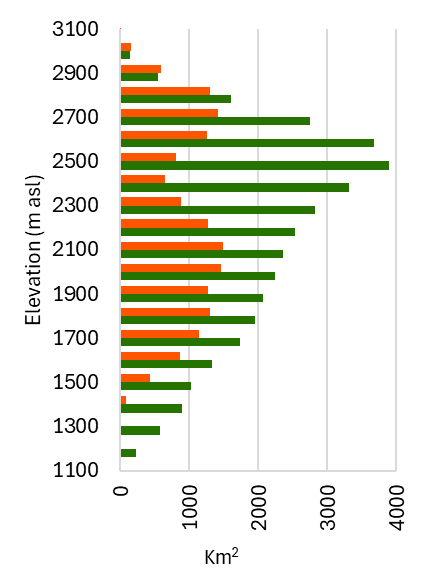 |

Figure S3.7*. Pinus discolor* current (A) and future (B-E) distribution models. The graph (F) illustrates the approximate area occupied by the potential distribution of the species in each altitudinal interval in the Sierra Madre Occidental in the current scenario (dark green) and in the 2100 scenario (dark orange).

| A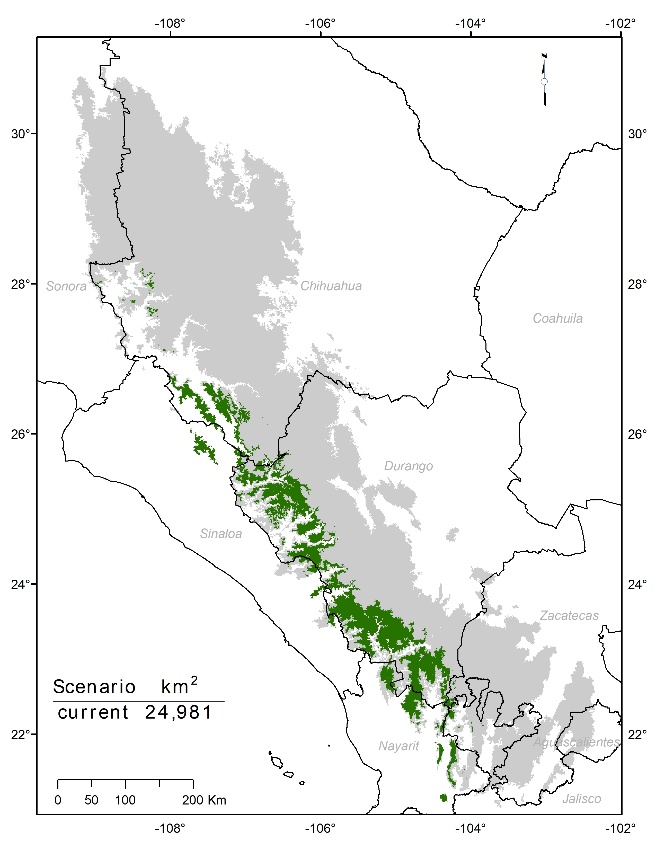 | B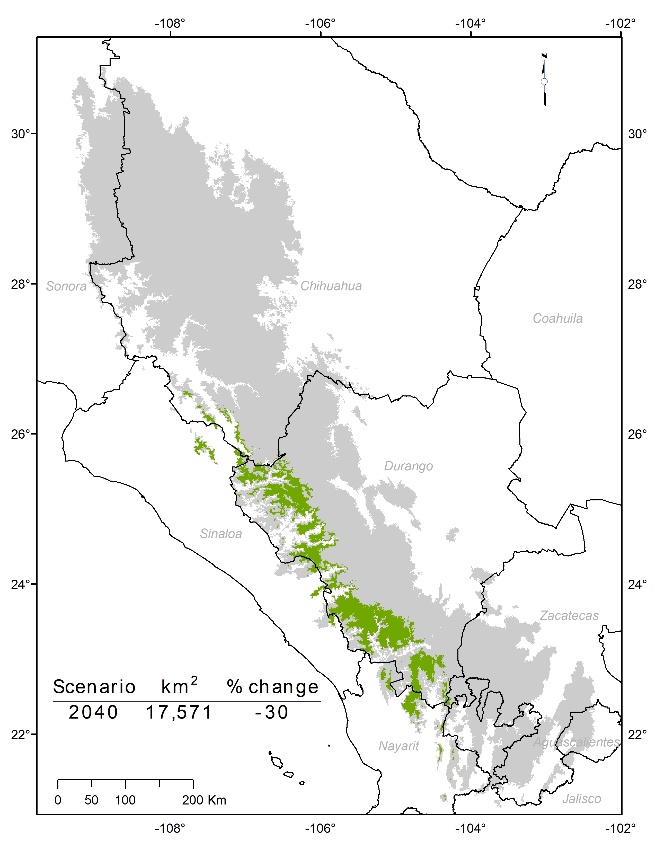 |
| --- | --- |
| C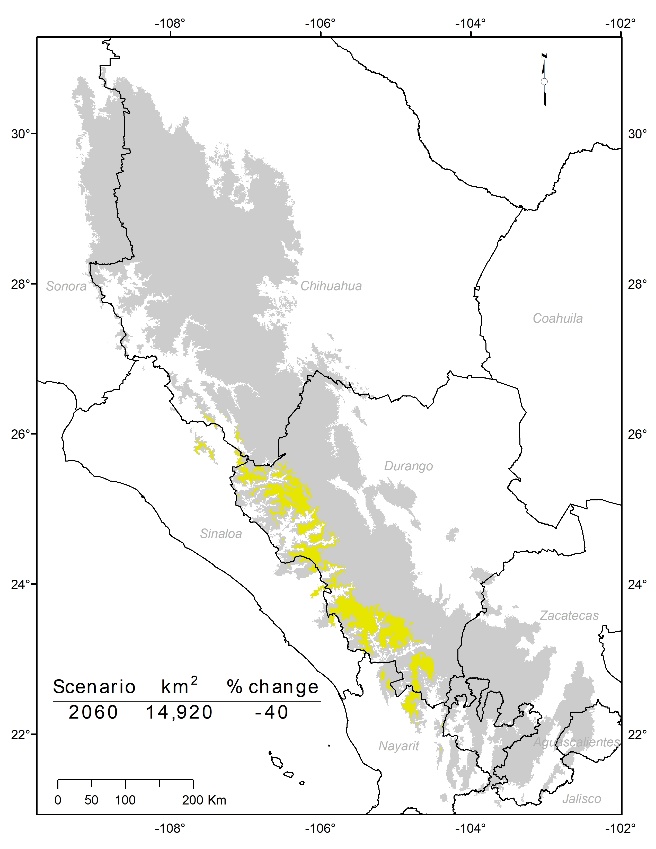 | D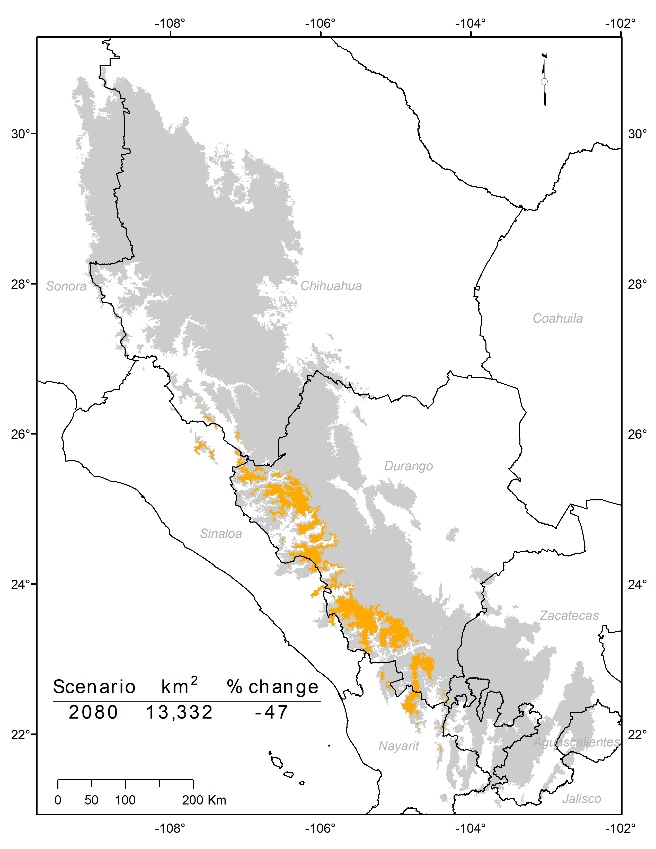 |
| E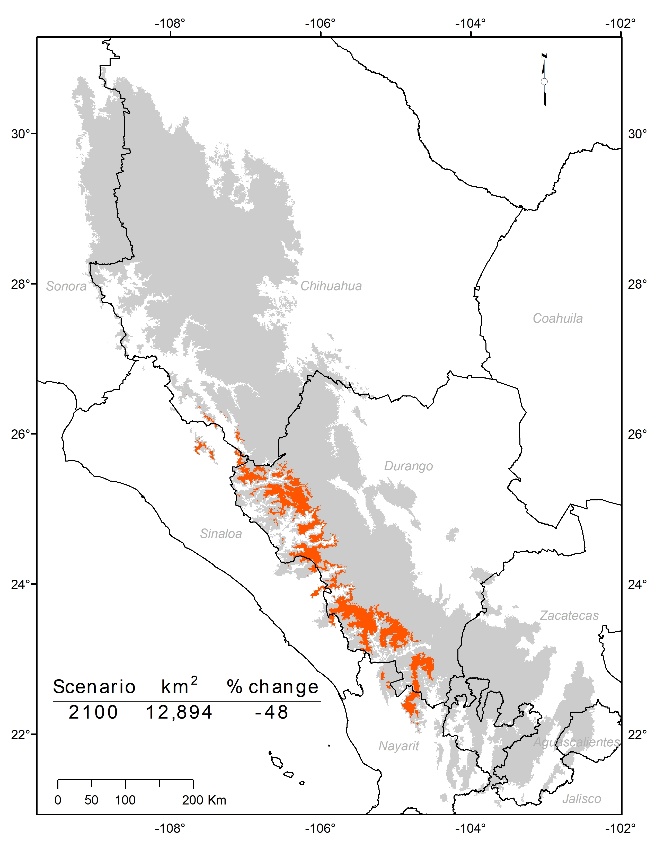 | F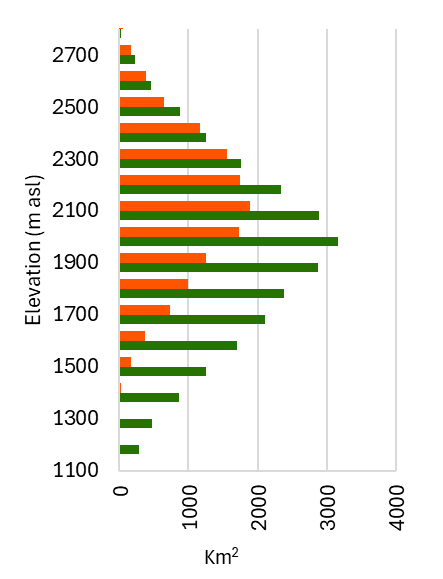 |

Figure S3.8. *Pinus douglasiana* current (A) and future (B-E) distribution models. The graph (F) illustrates the approximate area occupied by the potential distribution of the species in each altitudinal interval in the Sierra Madre Occidental in the current scenario (dark green) and in the 2100 scenario (dark orange).

| A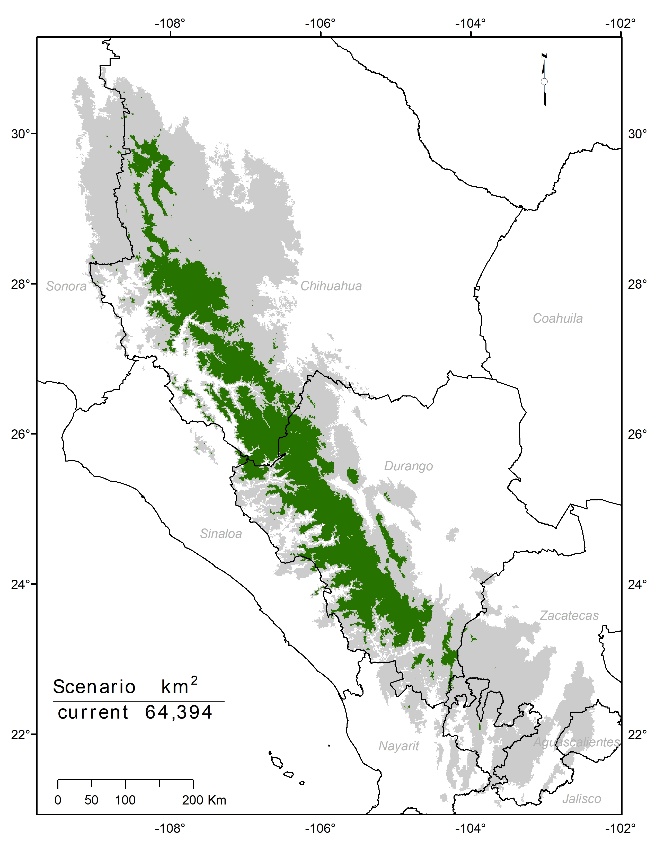 | B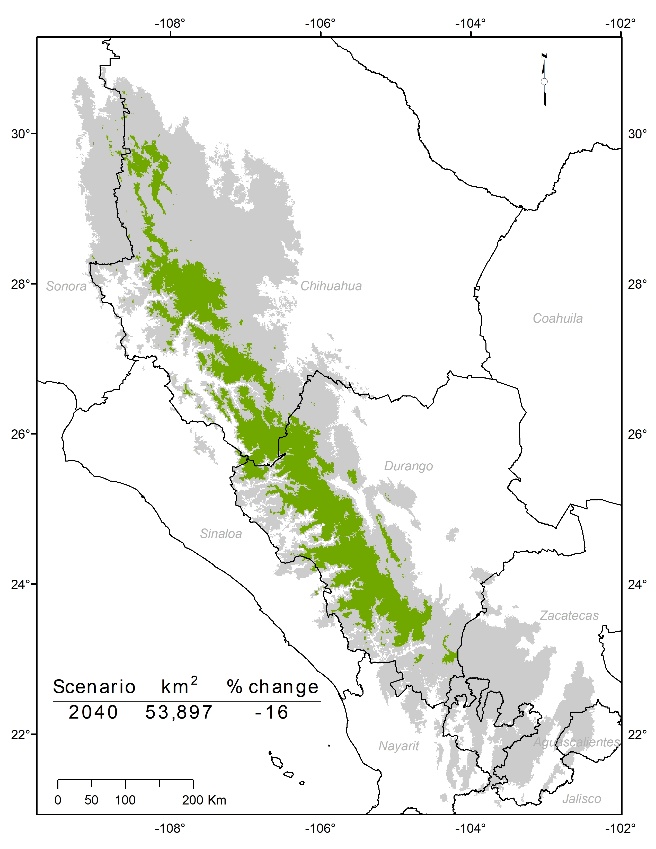 |
| --- | --- |
| C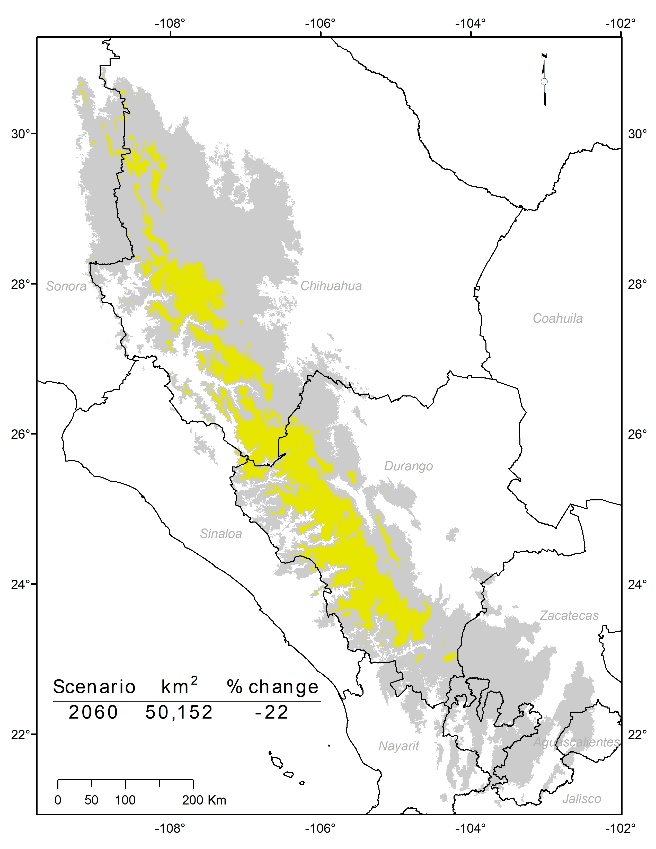 | D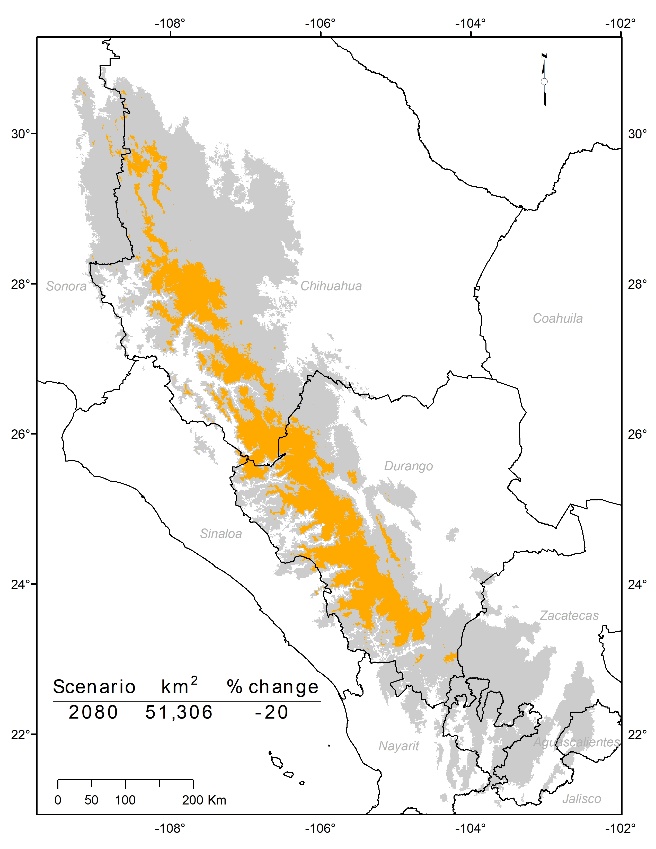 |
| E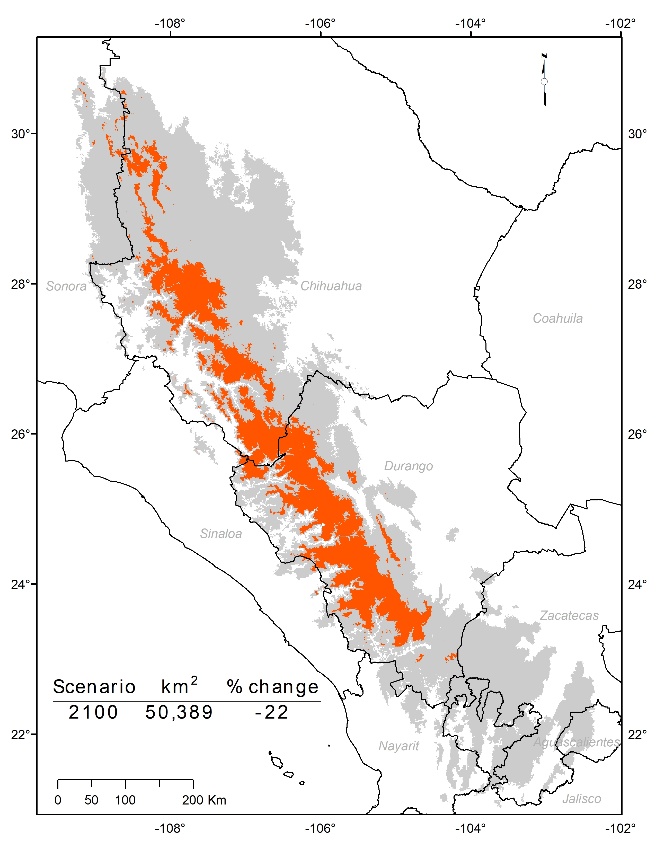 | F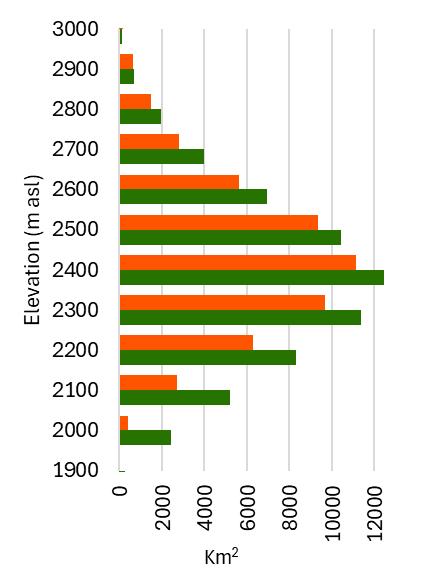 |

Figure S3.9. *Pinus durangensis* current (A) and future (B-E) distribution models. The graph (F) illustrates the approximate area occupied by the potential distribution of the species in each altitudinal interval in the Sierra Madre Occidental in the current scenario (dark green) and in the 2100 scenario (dark orange).

| A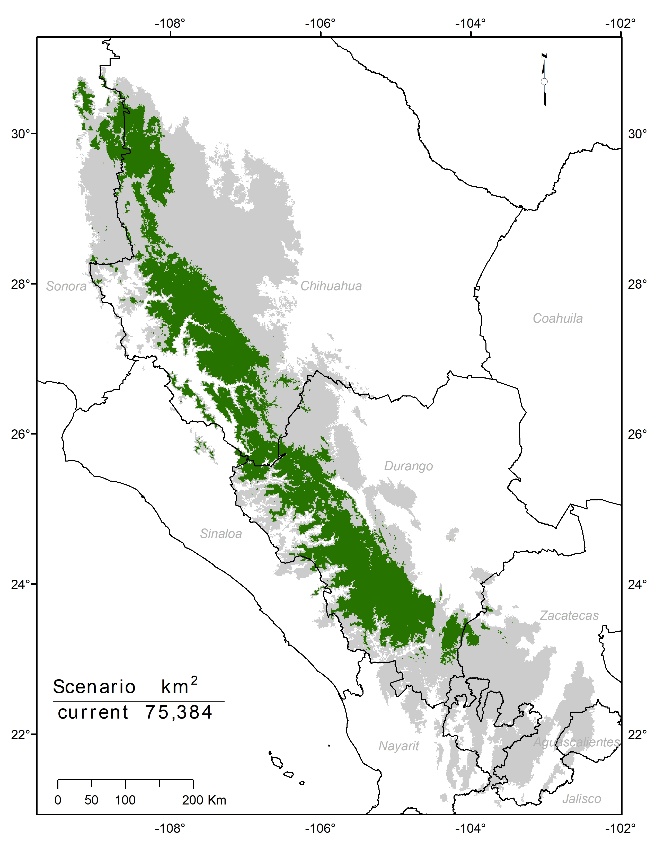 | B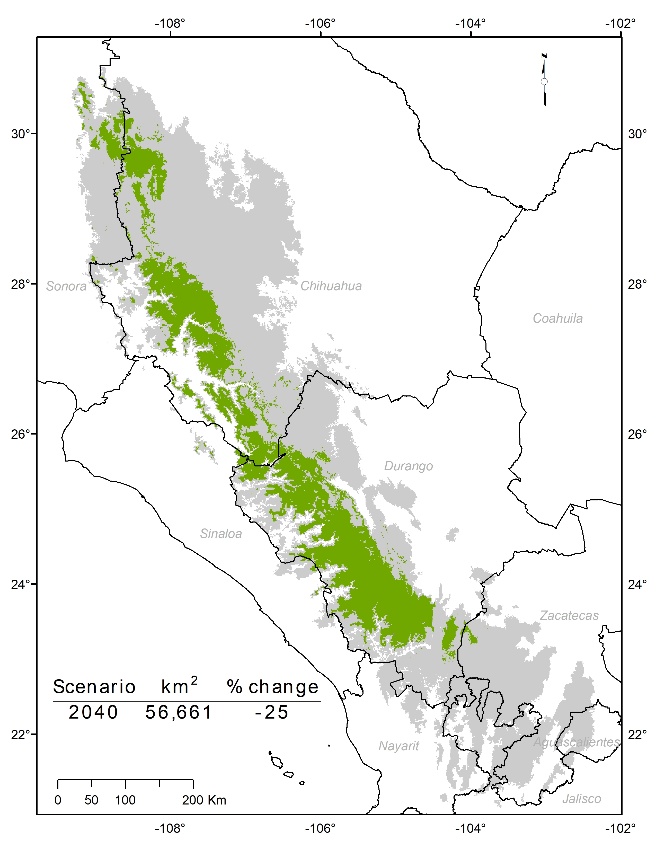 |
| --- | --- |
| C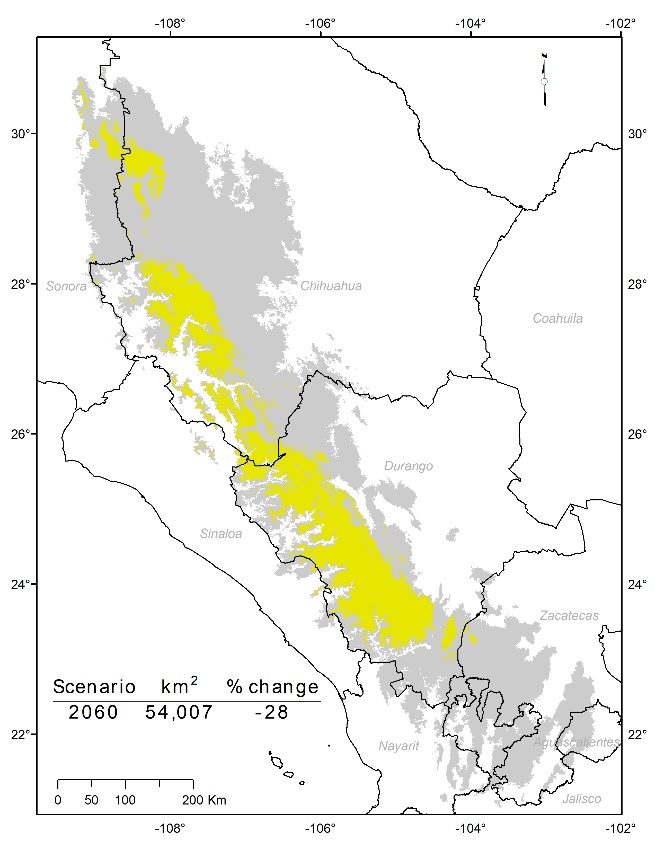 | D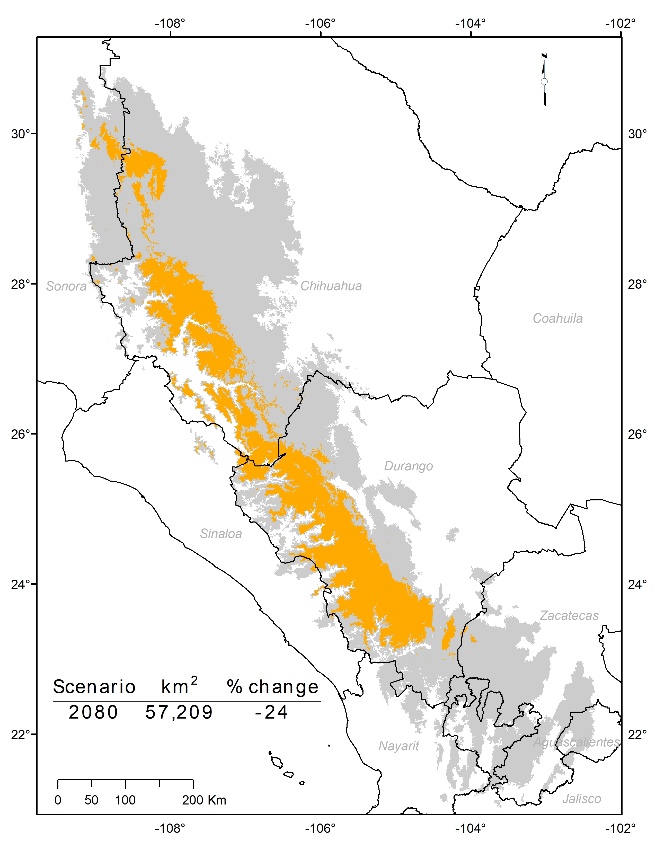 |
| E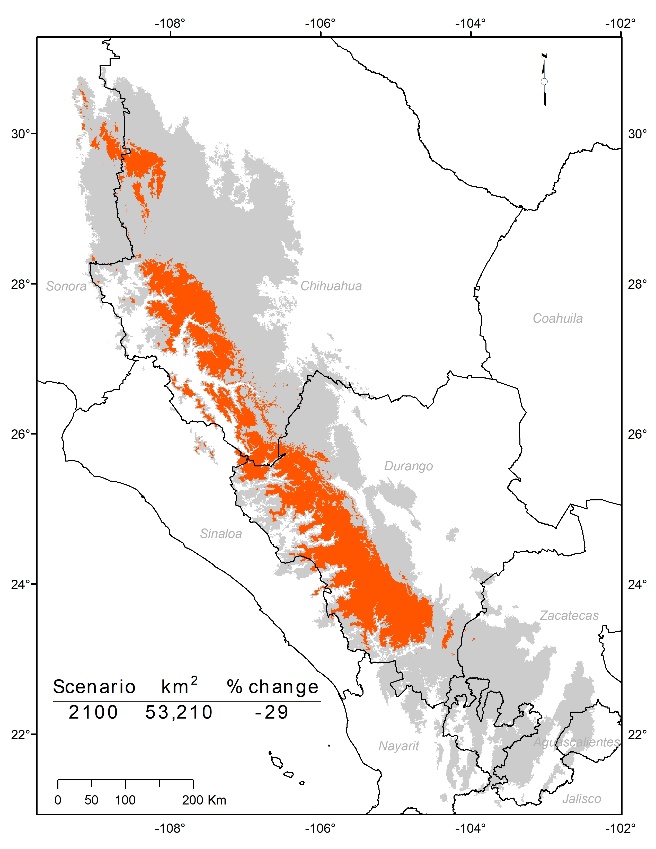 | F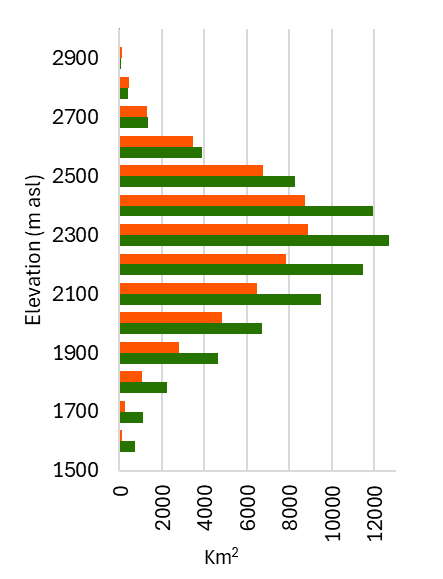 |

Figure S3.10. *Pinus emgelmannii* current (A) and future (B-E) distribution models. The graph (F) illustrates the approximate area occupied by the potential distribution of the species in each altitudinal interval in the Sierra Madre Occidental in the current scenario (dark green) and in the 2100 scenario (dark orange).

| A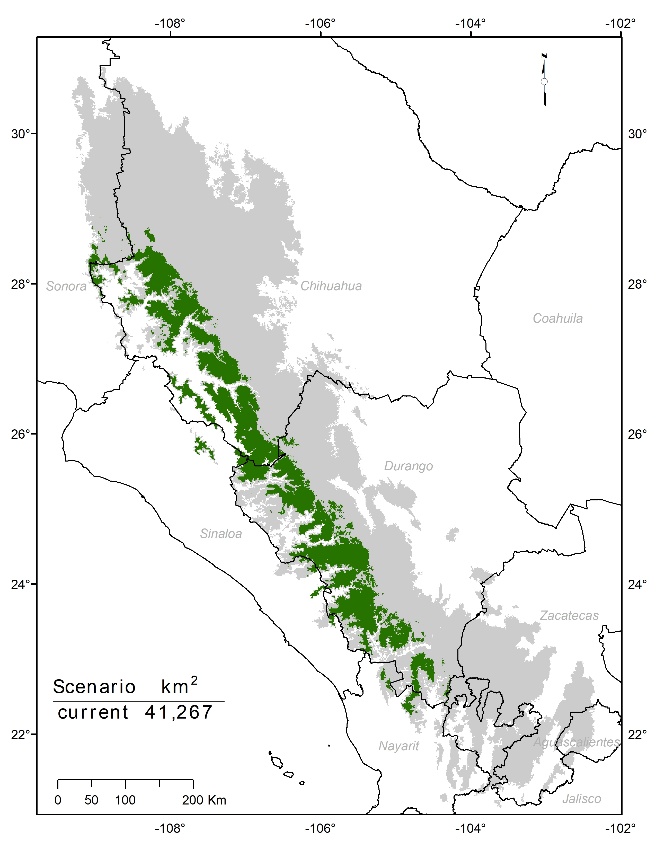 | B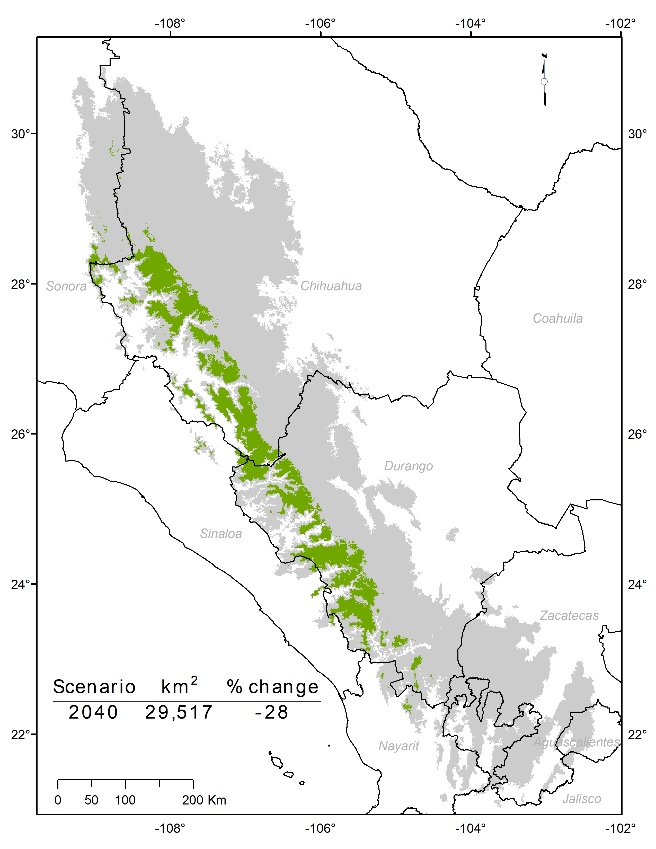 |
| --- | --- |
| C 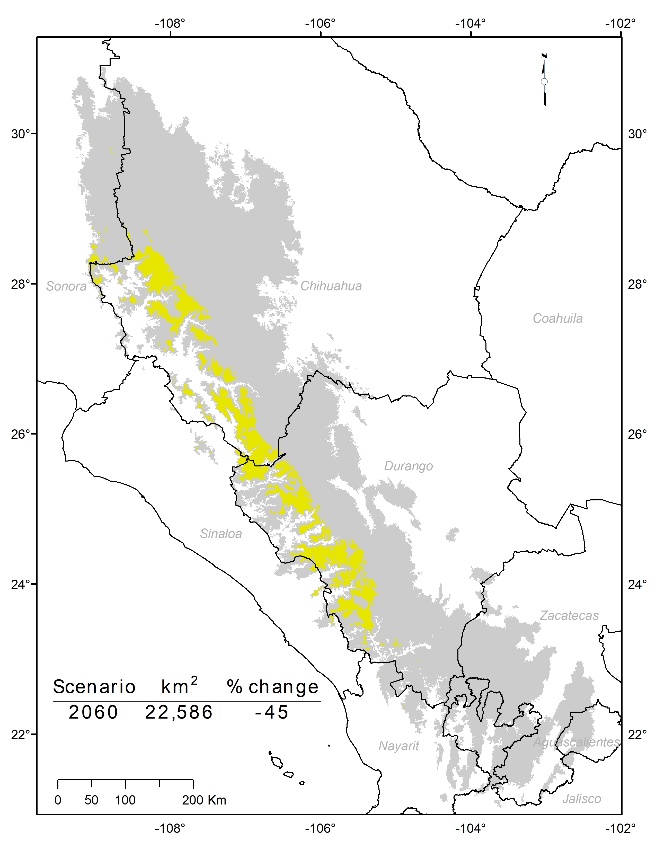 | D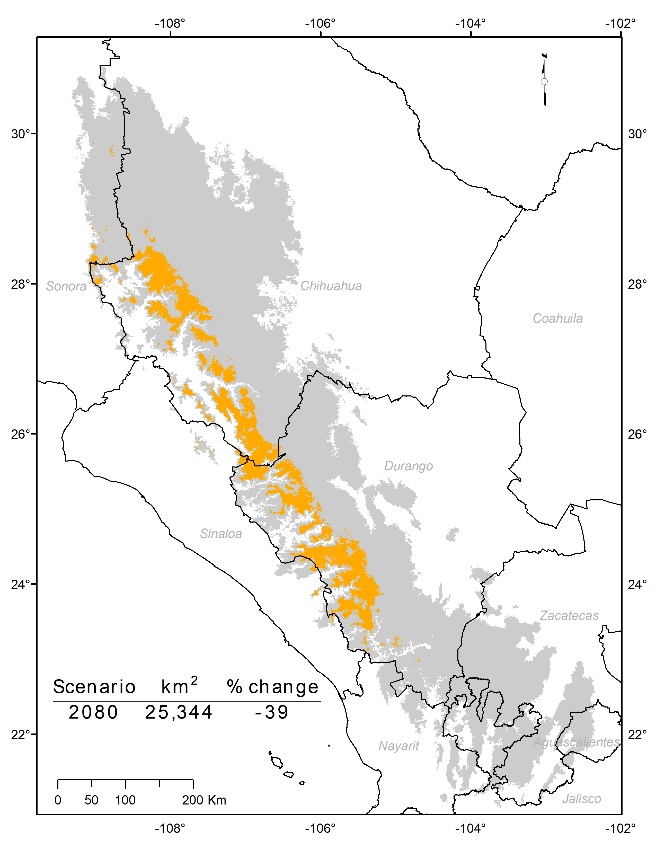 |
| E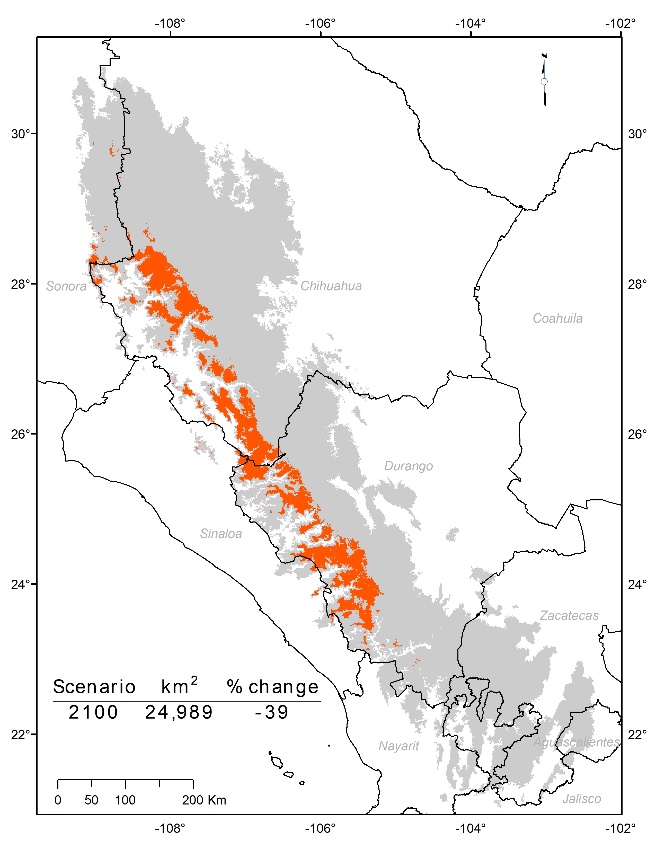 | F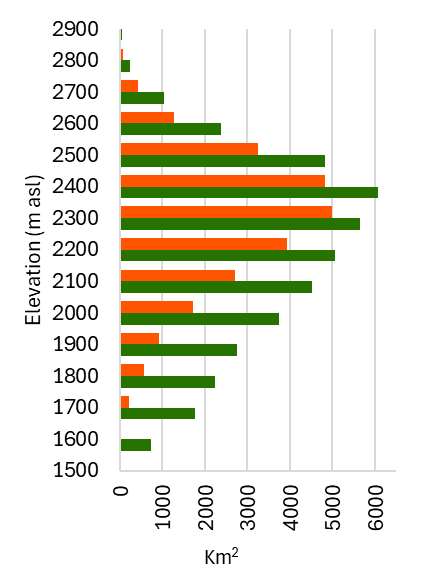 |

Figure S3.11. *Pinus herrerae* current (A) and future (B-E) distribution models. The graph (F) illustrates the approximate area occupied by the potential distribution of the species in each altitudinal interval in the Sierra Madre Occidental in the current scenario (dark green) and in the 2100 scenario (dark orange).

| A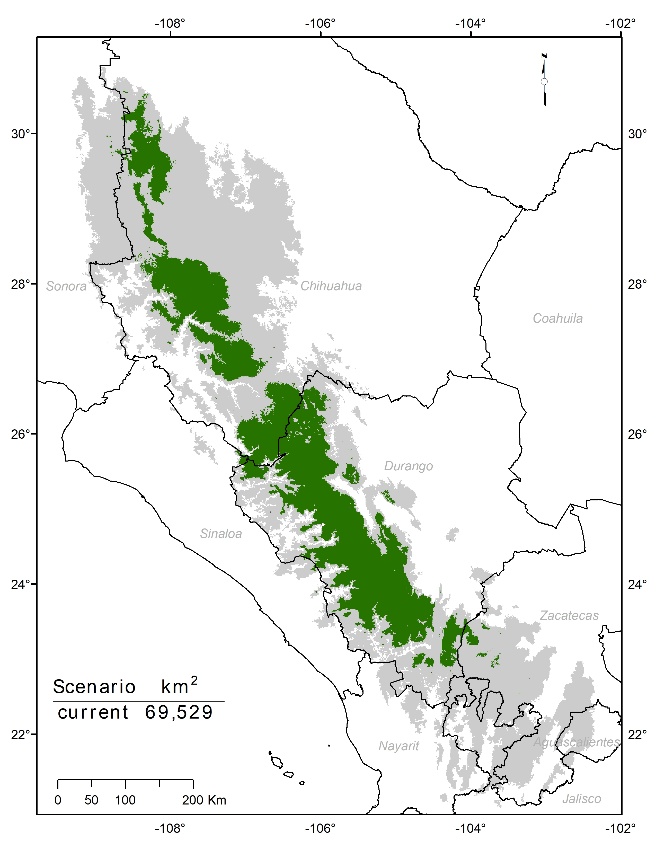 | B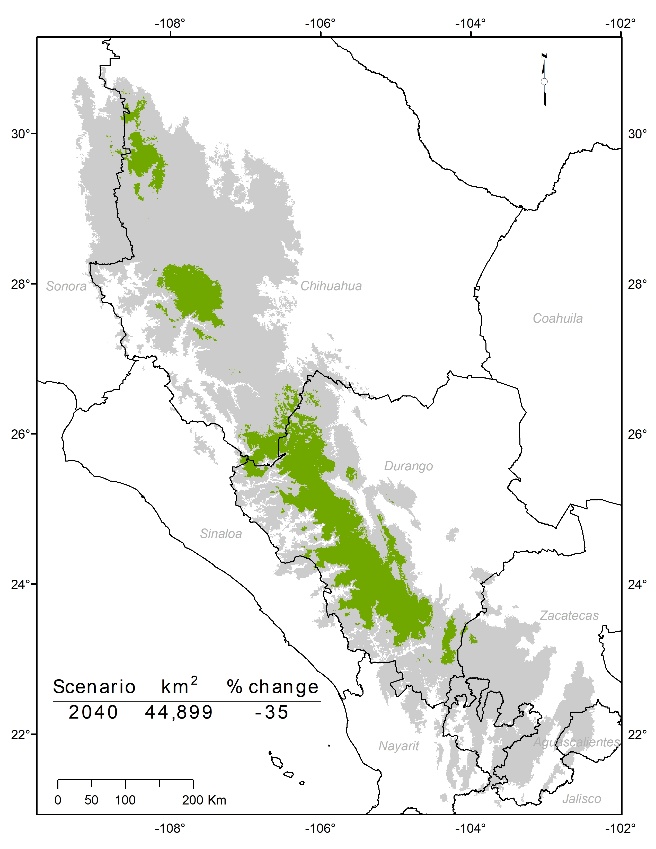 |
| --- | --- |
| C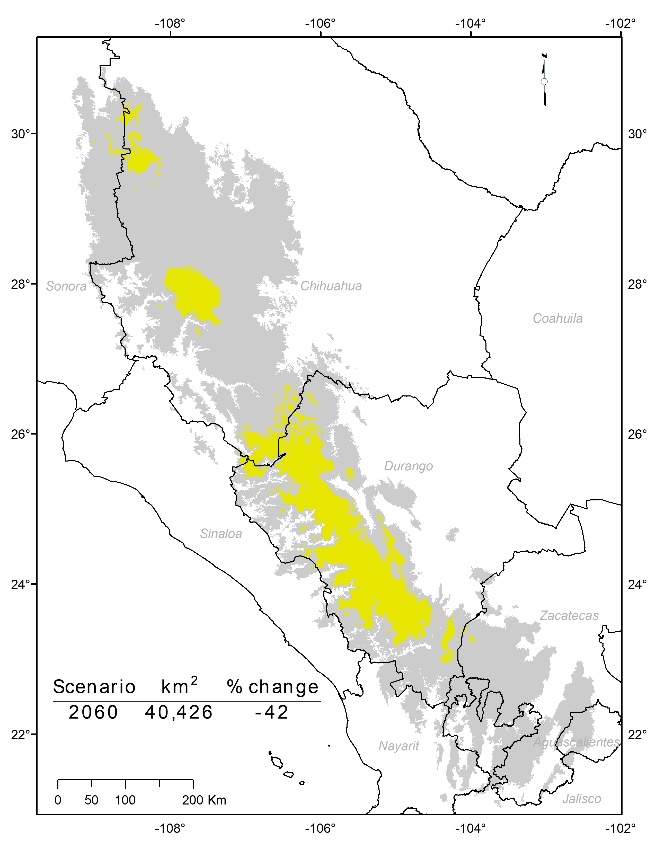 | D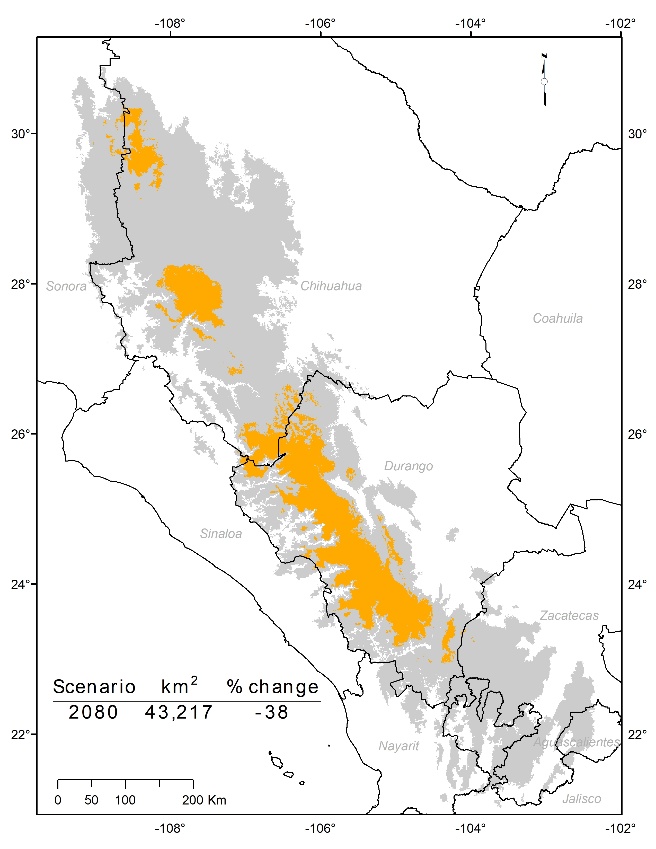 |
| E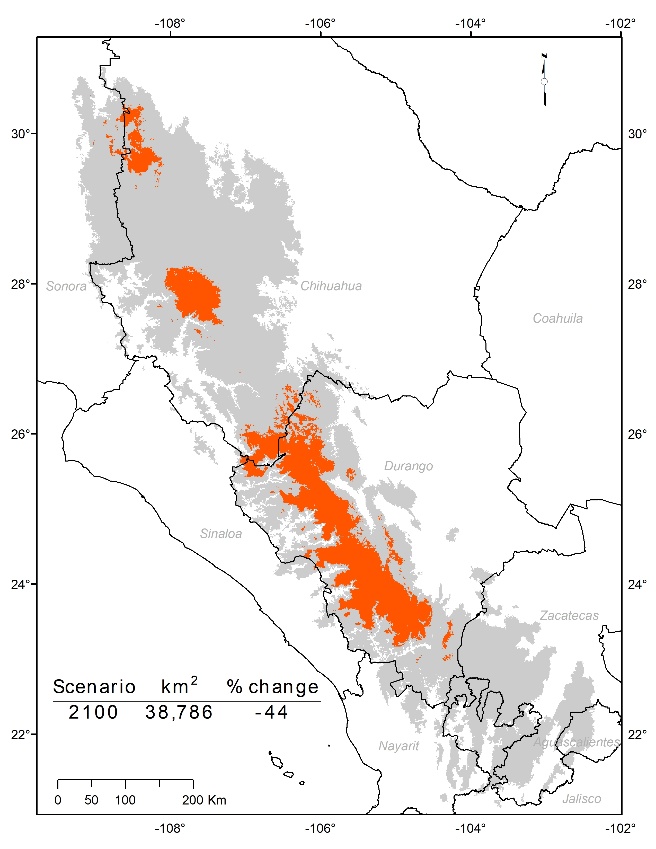 | F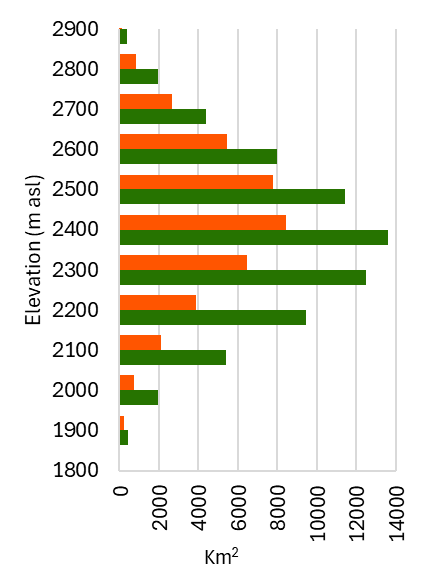 |

Figure S3.12. *Pinus leiophylla* current (A) and future (B-E) distribution models. The graph (F) illustrates the approximate area occupied by the potential distribution of the species in each altitudinal interval in the Sierra Madre Occidental in the current scenario (dark green) and in the 2100 scenario (dark orange).

| A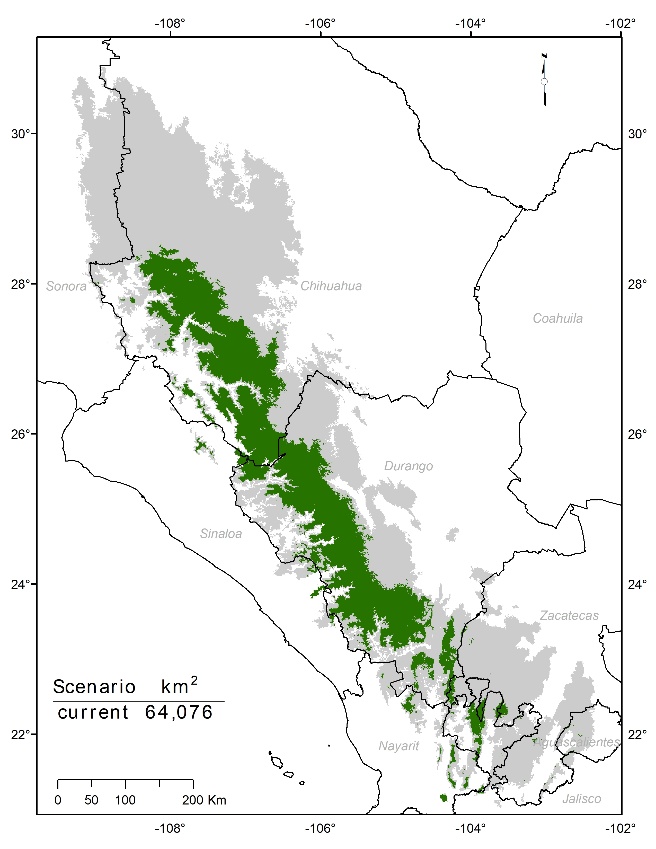 | B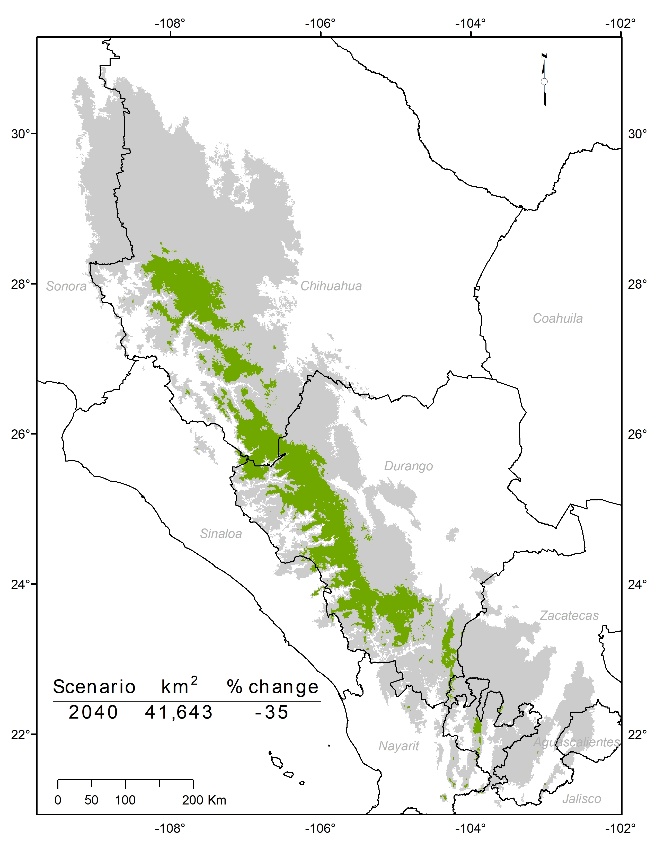 |
| --- | --- |
| C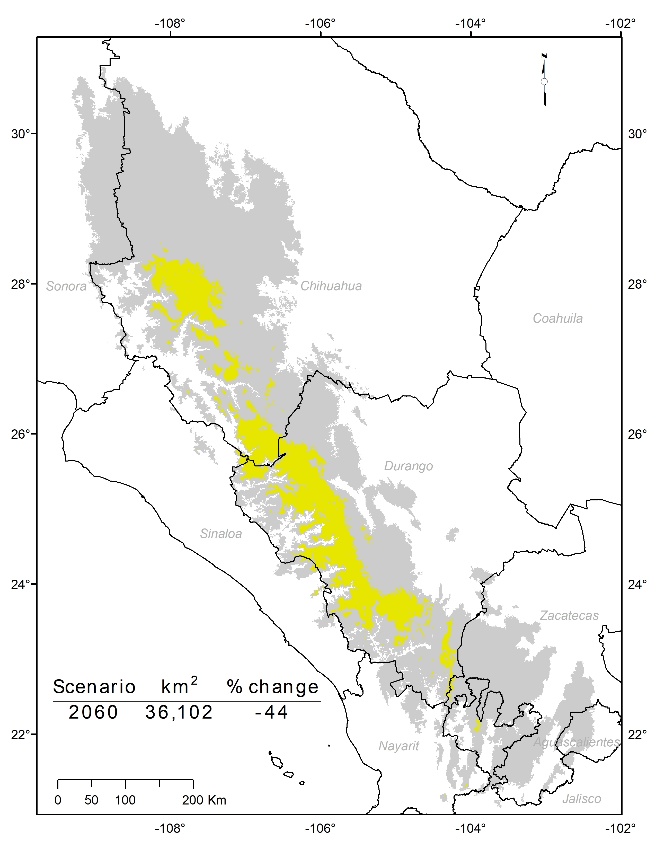 | D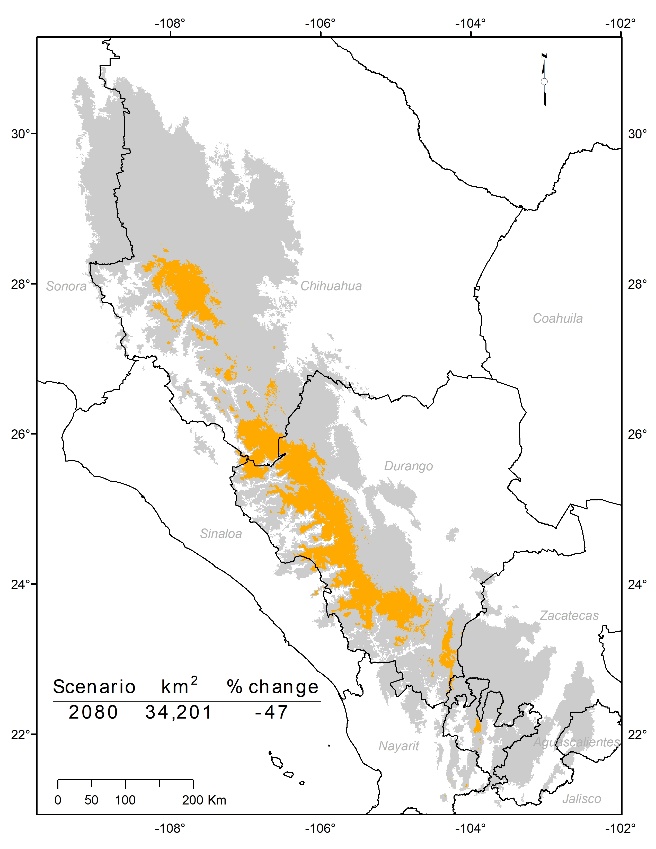 |
| E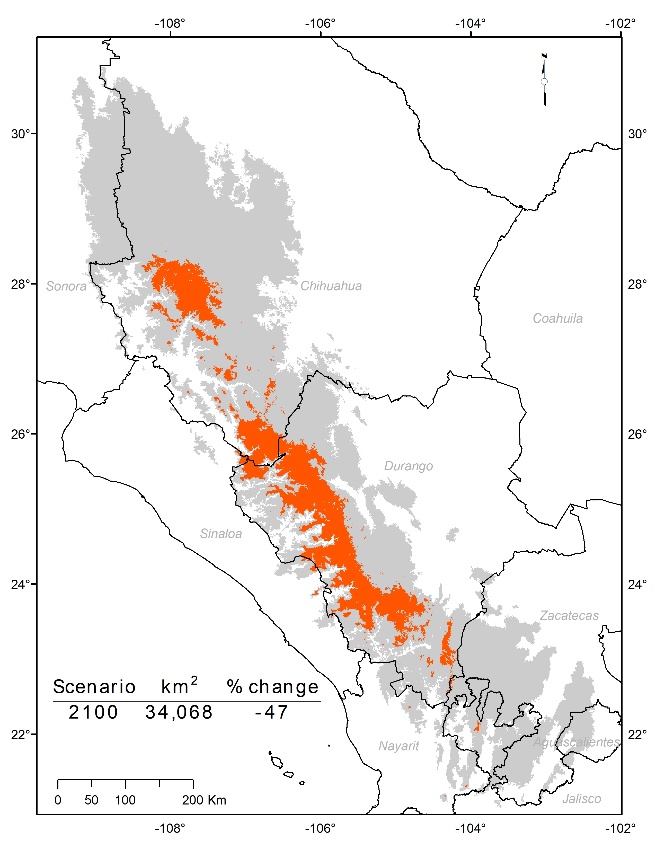 | F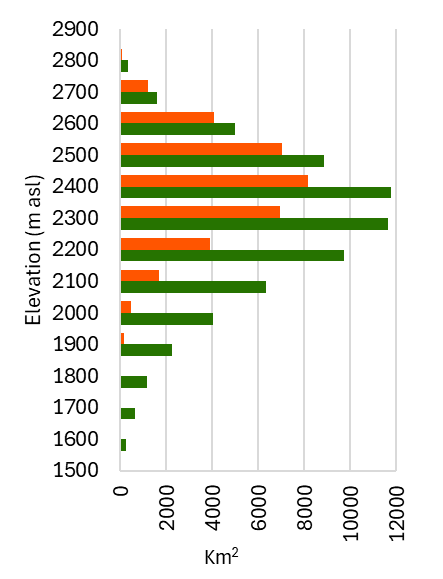 |

Figure S3.13. *Pinus lumholtzii* current (A) and future (B-E) distribution models. The graph (F) illustrates the approximate area occupied by the potential distribution of the species in each altitudinal interval in the Sierra Madre Occidental in the current scenario (dark green) and in the 2100 scenario (dark orange).

| A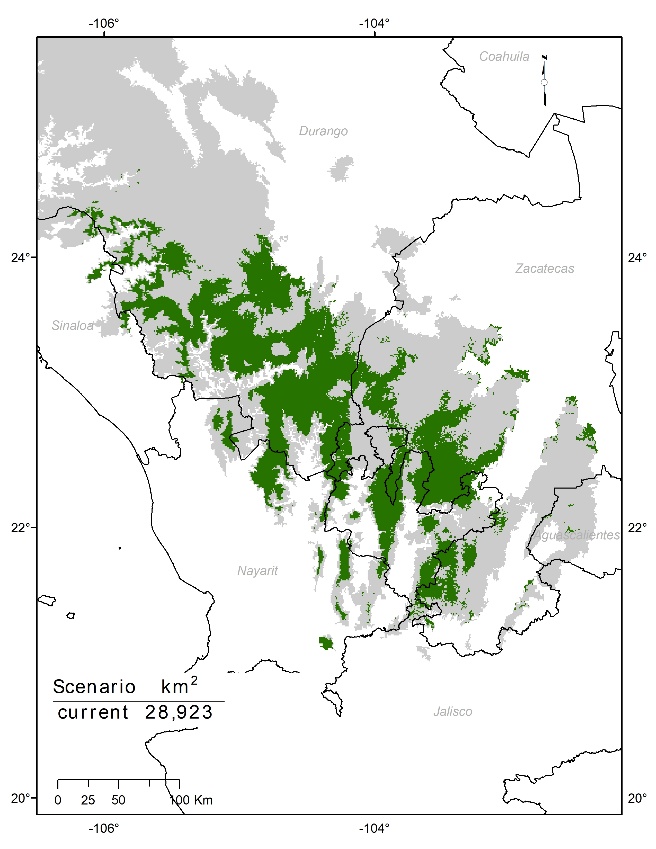 | B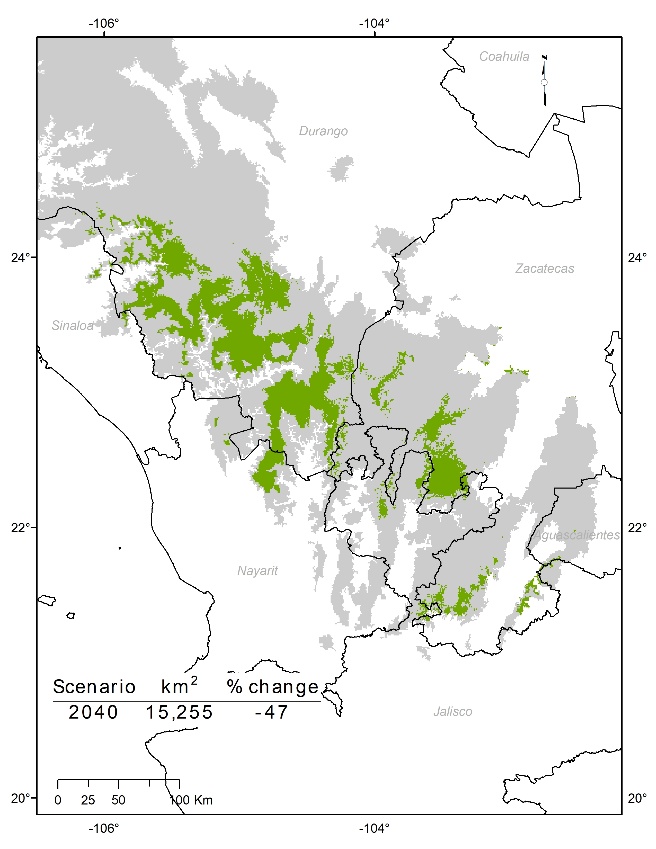 |
| --- | --- |
| C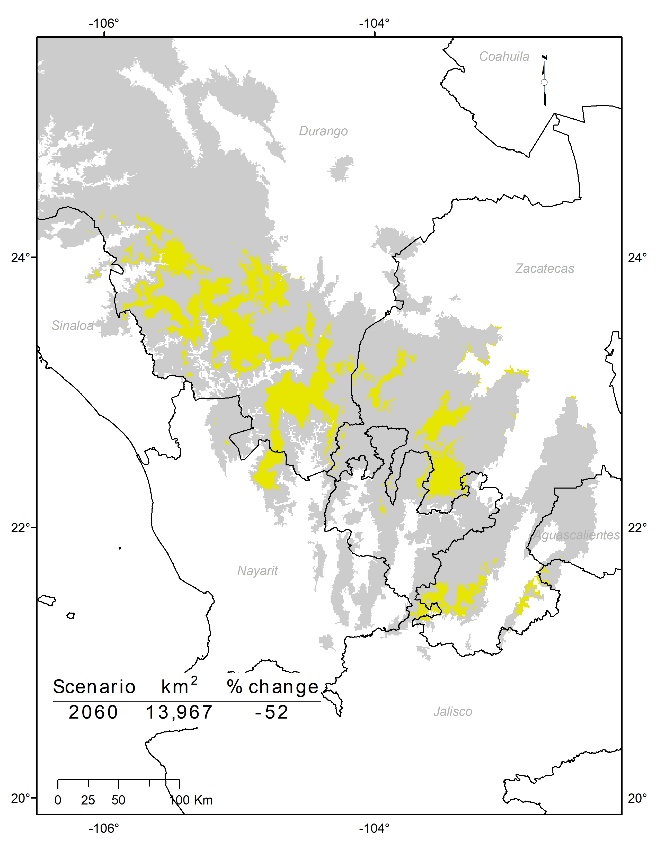 | D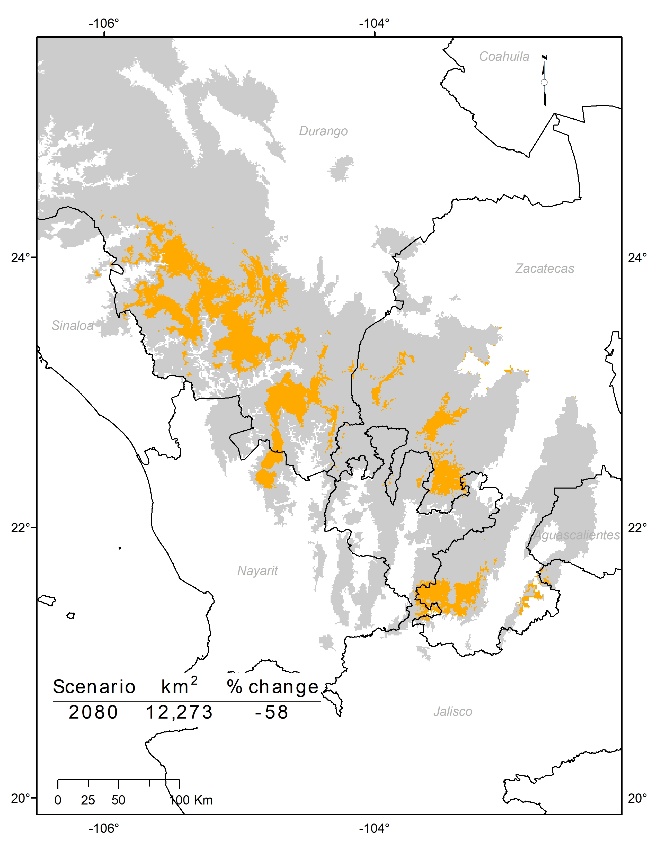 |
| E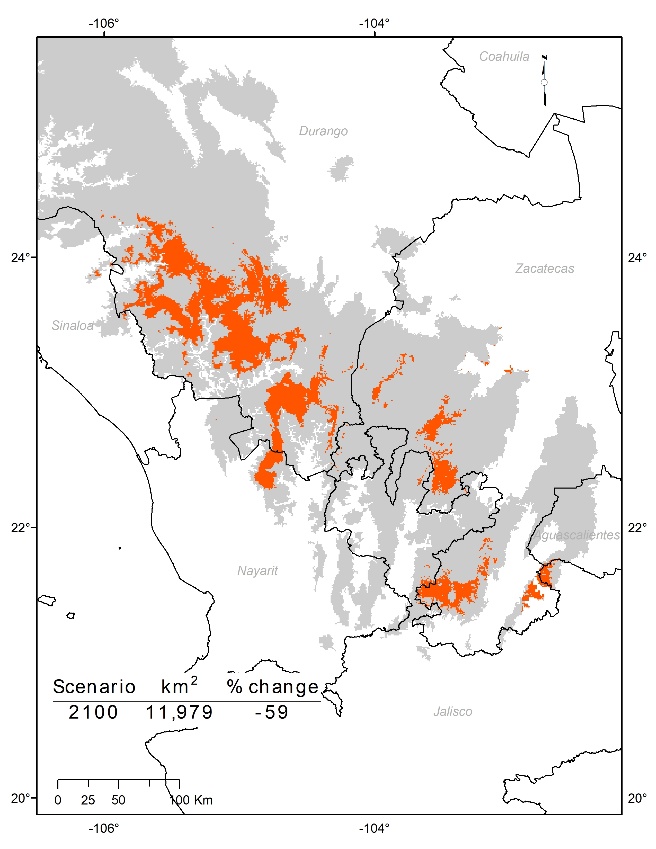 | F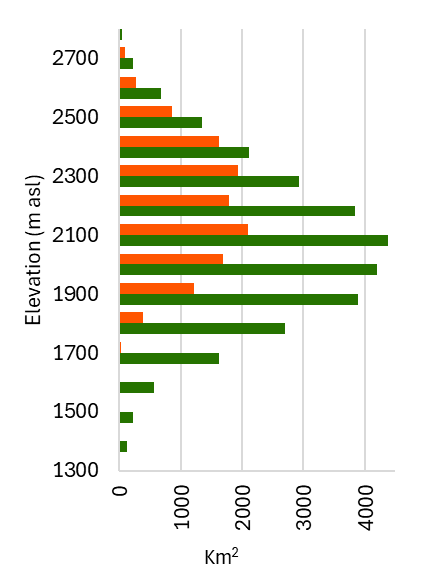 |

Figure S3.14. *Pinus luzmariae* current (A) and future (B-E) distribution models. The graph (F) illustrates the approximate area occupied by the potential distribution of the species in each altitudinal interval in the Sierra Madre Occidental in the current scenario (dark green) and in the 2100 scenario (dark orange).

| A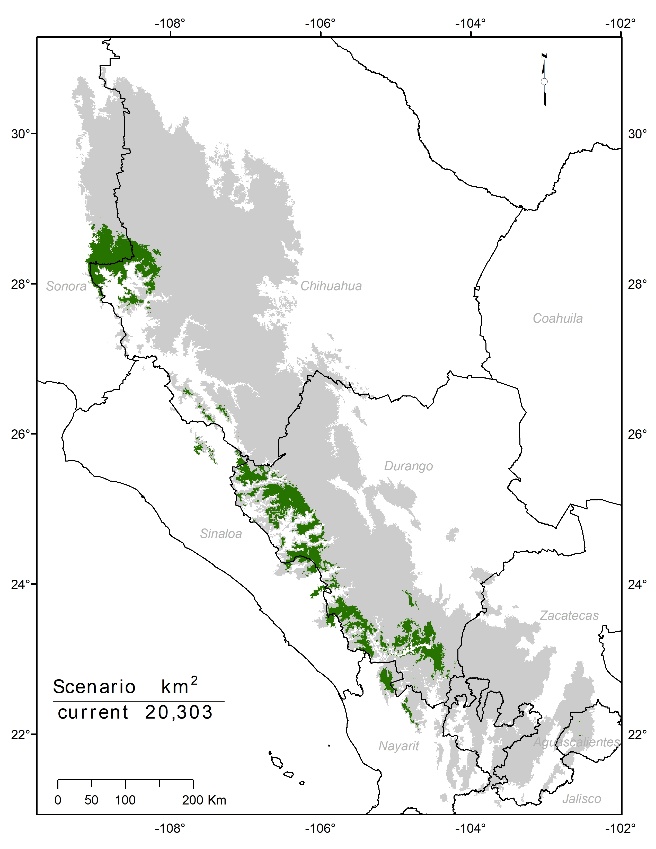 | B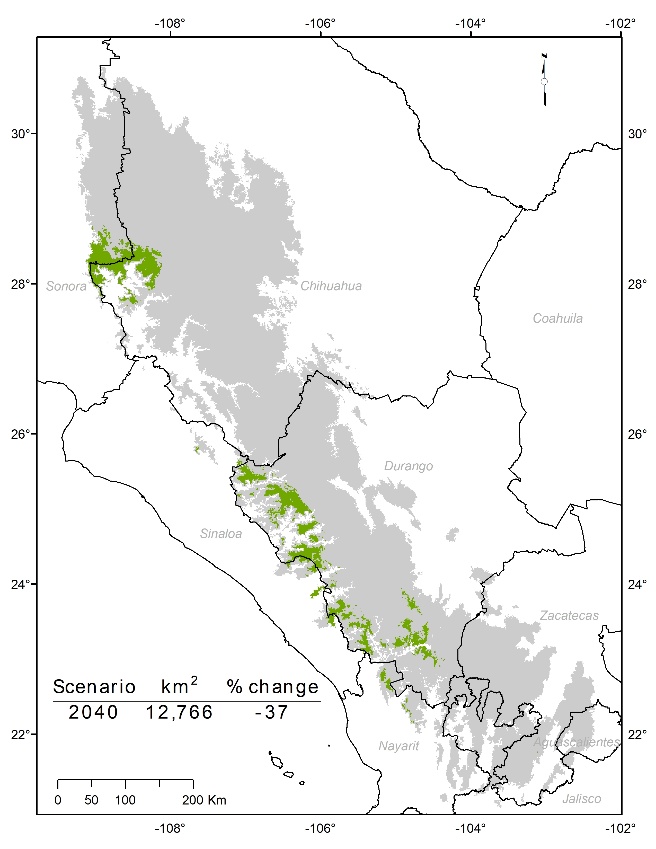 |
| --- | --- |
| C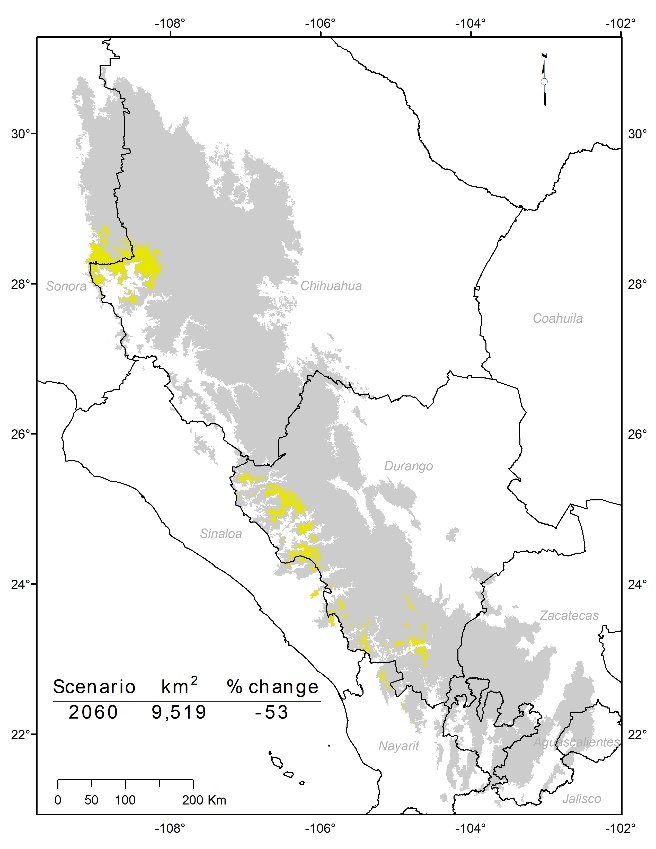 | D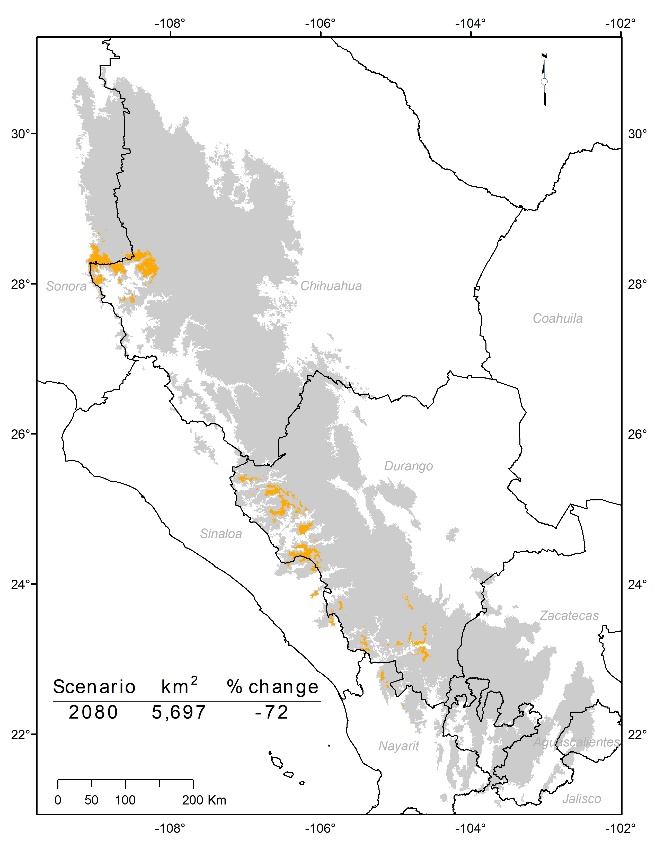 |
| E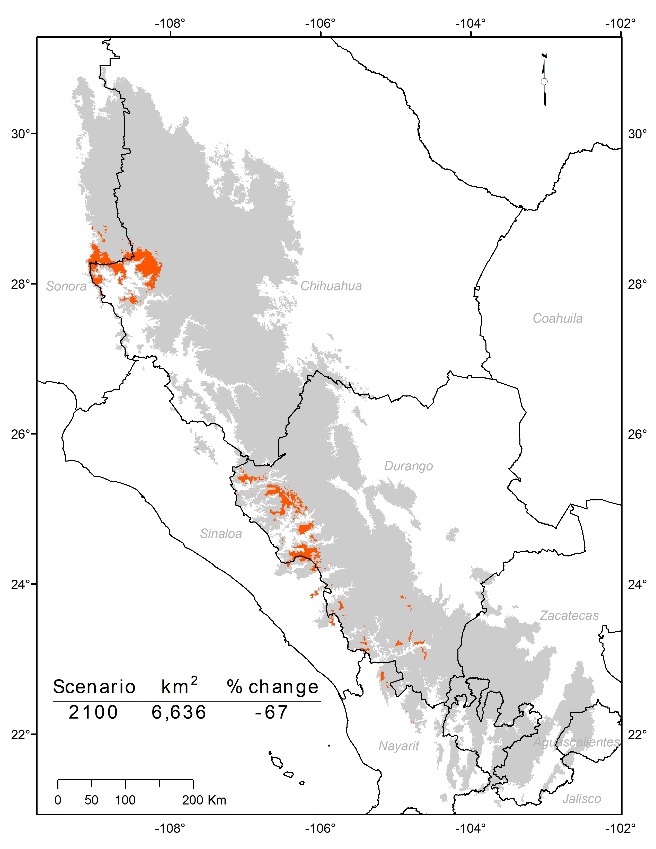 | F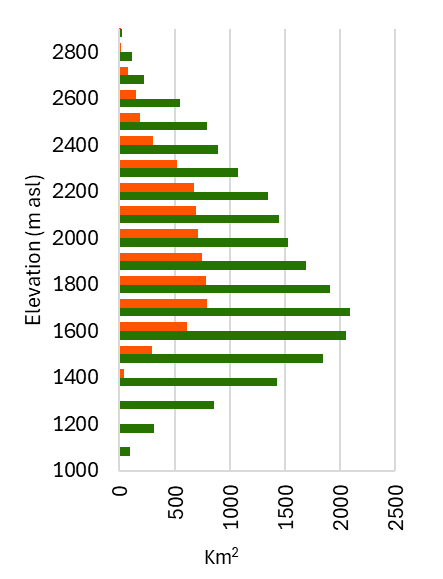 |

Figure S3.15. *Pinus maximinoi* current (A) and future (B-E) distribution models. The graph (F) illustrates the approximate area occupied by the potential distribution of the species in each altitudinal interval in the Sierra Madre Occidental in the current scenario (dark green) and in the 2100 scenario (dark orange).

| A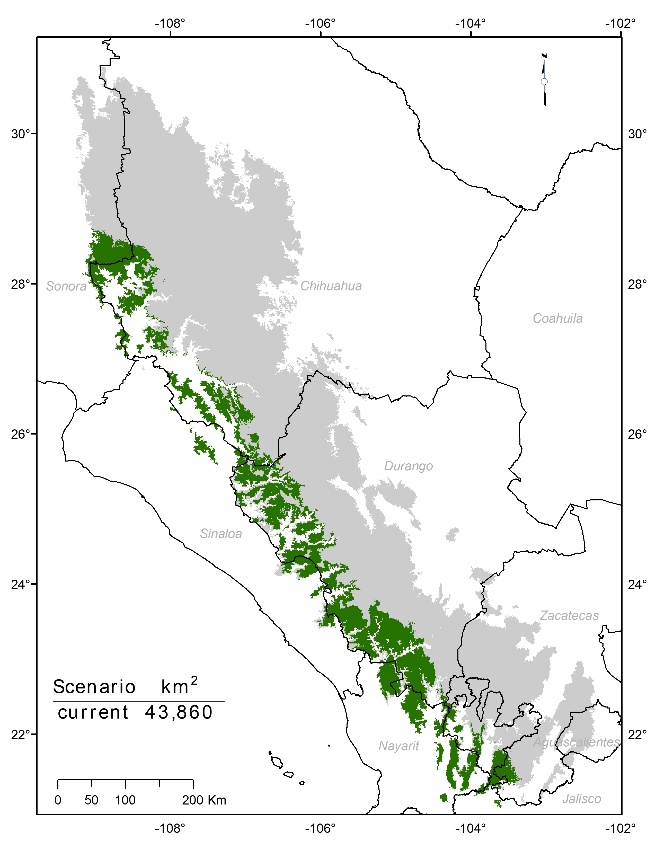 | B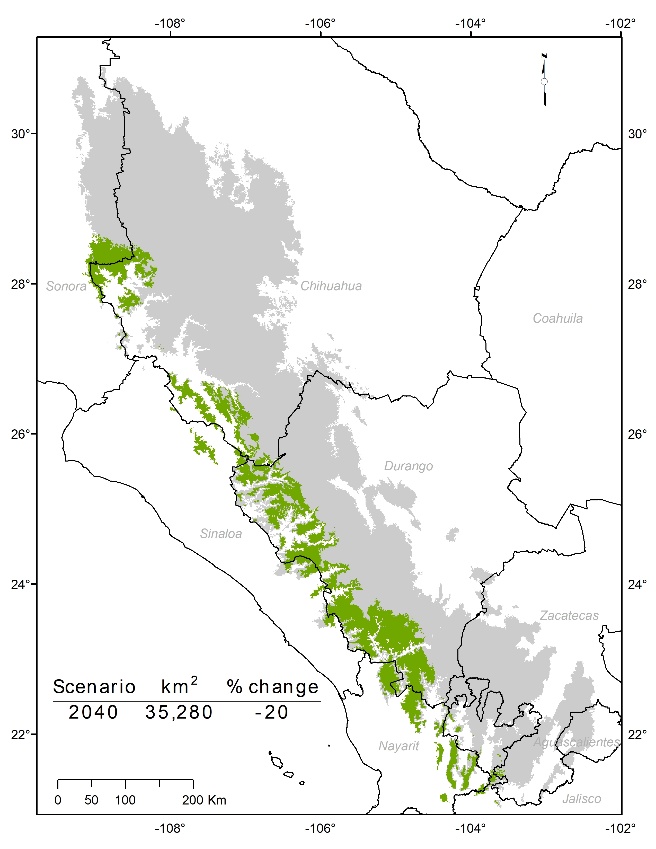 |
| --- | --- |
| C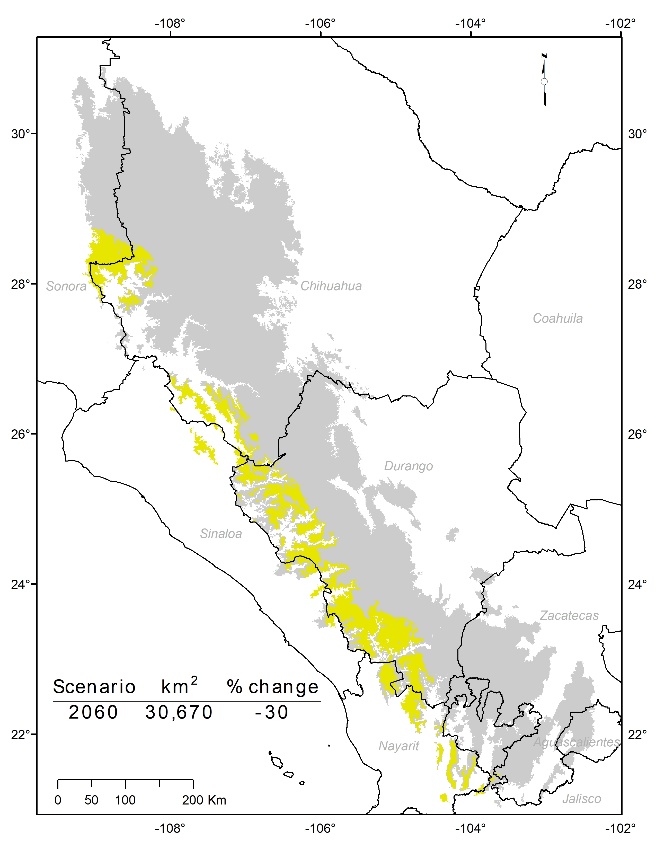 | D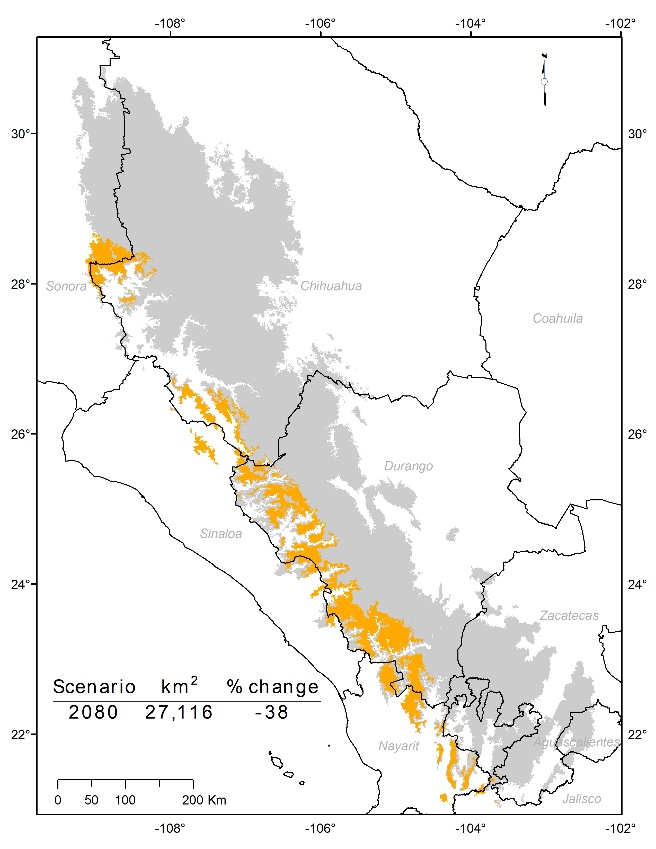 |
| E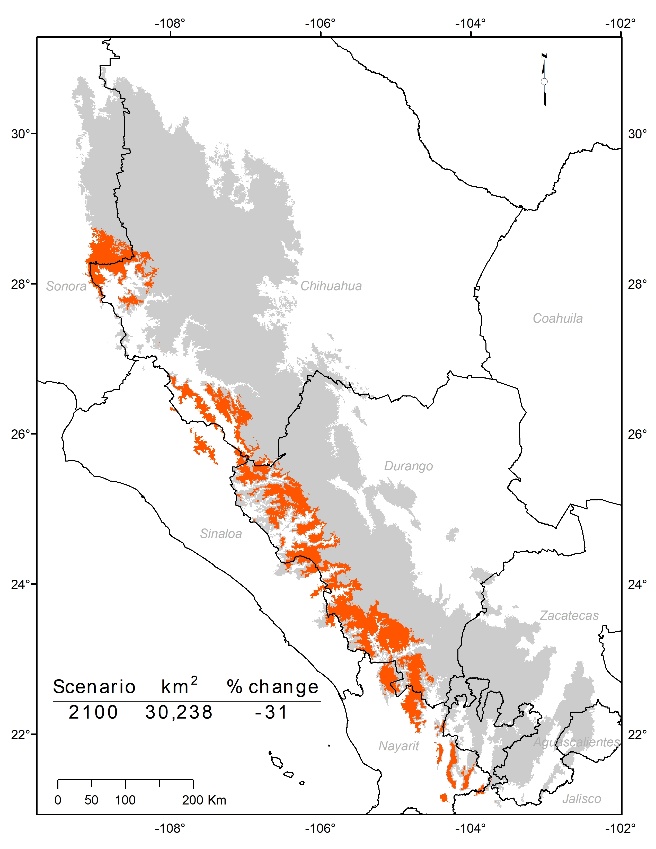 | F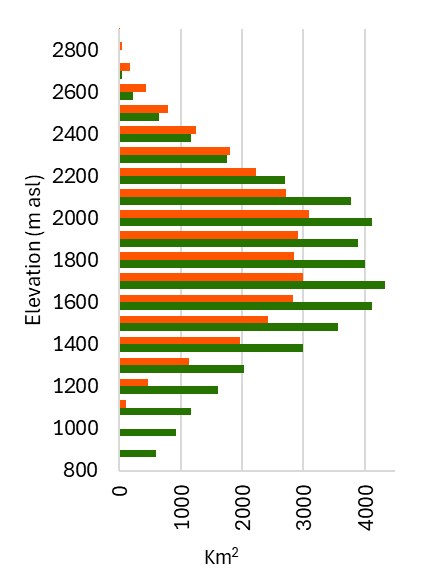 |

Figure S3.16. *Pinus oocarpa* current (A) and future (B-E) distribution models. The graph (F) illustrates the approximate area occupied by the potential distribution of the species in each altitudinal interval in the Sierra Madre Occidental in the current scenario (dark green) and in the 2100 scenario (dark orange).

| A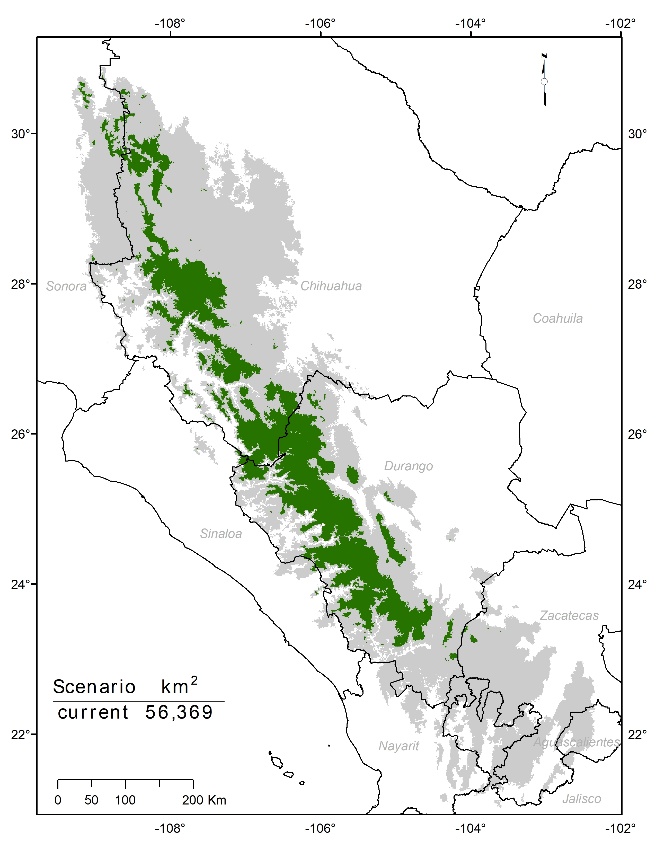 | B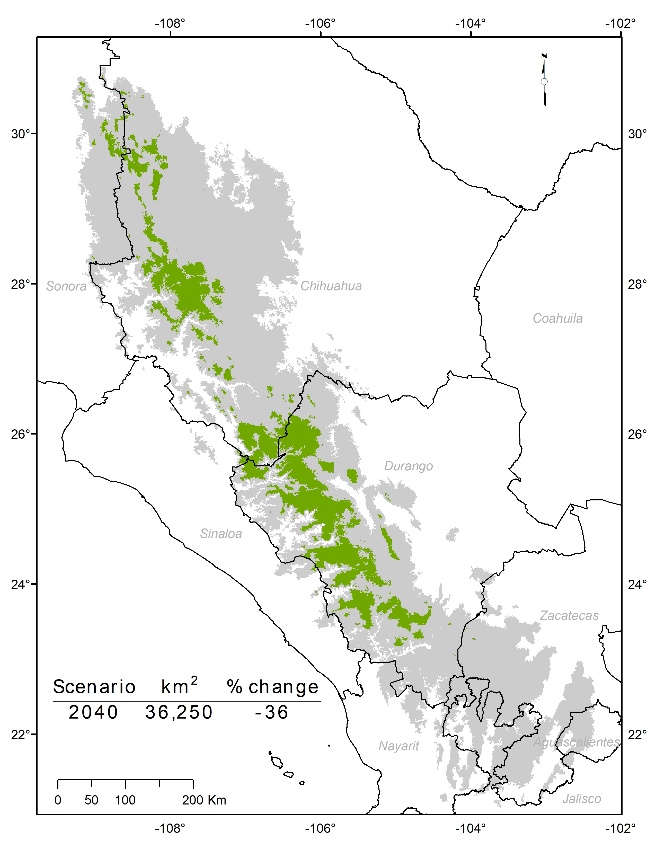 |
| --- | --- |
| C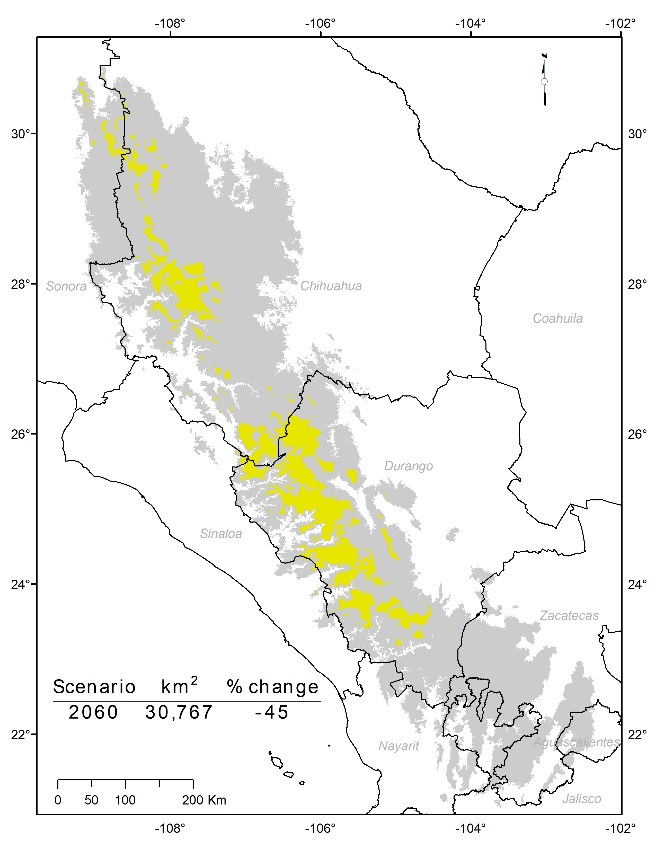 | D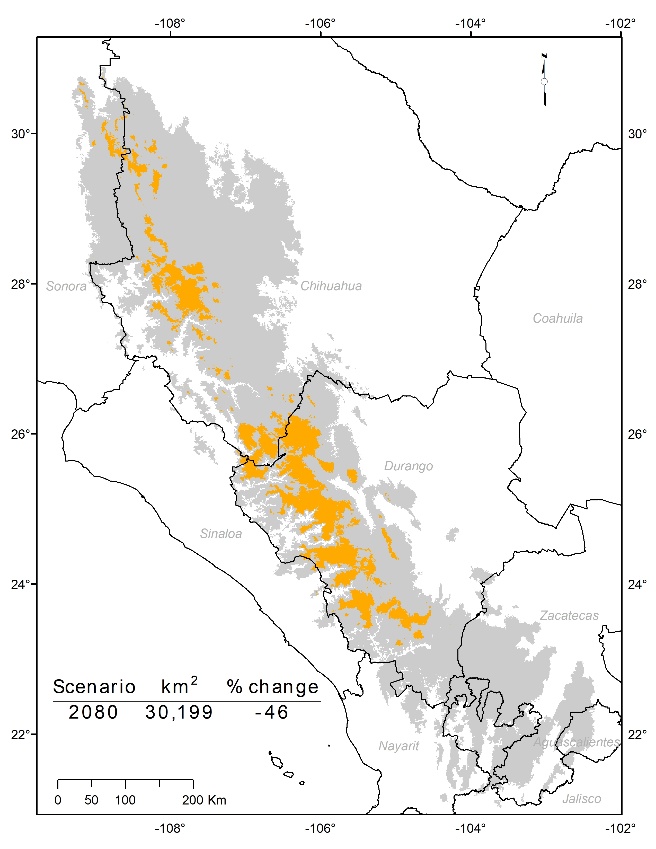 |
| E | F |

Figure S3.17. *Pinus strobiformis* current (A) and future (B-E) distribution models. The graph (F) illustrates the approximate area occupied by the potential distribution of the species in each altitudinal interval in the Sierra Madre Occidental in the current scenario (dark green) and in the 2100 scenario (dark orange).

| A | B |
| --- | --- |
| C | D |
| E | F |

Figure S3.18. *Pinus teocote* current (A) and future (B-E) distribution models. The graph (F) illustrates the approximate area occupied by the potential distribution of the species in each altitudinal interval in the Sierra Madre Occidental in the current scenario (dark green) and in the 2100 scenario (dark orange).

| A | B |
| --- | --- |
| C | D |
| E | F |

Figure S3.19. *Pinus yecorensis* current (A) and future (B-E) distribution models. The graph (F) illustrates the approximate area occupied by the potential distribution of the species in each altitudinal interval in the Sierra Madre Occidental in the current scenario (dark green) and in the 2100 scenario (dark orange).
