# Supplementary material for: Diversity Hotspots and Vulnerability of Pine Species in the Sierra Madre Occidental, Western Mexico
Source: Ecol Evol. 2025 Jul 9;15(7):e71743. doi: 10.1002/ece3.71743 (PMC12240594; doi:10.1002/ece3.71743)
Supplement: Supplementary file 1 — Appendix S1. [file ECE3-15-e71743-s001.zip › ece371743-sup-0002-Figures.docx]

**Supporting Information 1 for**

**Diversity hotspots and vulnerability of pine species in the Sierra Madre Occidental, western Mexico**

CONTENTS:

[Figure S1.1. Template ecoregions of Sierra Madre Occidental. 3](#_Toc149740182)

[Figure S1.2. *Pinus arizonica* current and future distribution models. 3](#_Toc149740183)

[Figure S1.3. *Pinus brachyptera* current and future distribution models. 4](#_Toc149740184)

[Figure S1.4. *Pinus cembroides* current and future distribution models. 4](#_Toc149740185)

[Figure S1.5. *Pinus chihuahuana* current and future distribution models. 5](#_Toc149740186)

[Figure S1.6. *Pinus cooperi* current and future distribution models. 5](#_Toc149740187)

[Figure S1.7. *Pinus devoniana* current and future distribution models. 6](#_Toc149740188)

[Figure S1.8. *Pinus discolor* current and future distribution models. 6](#_Toc149740189)

[Figure S1.9. *Pinus douglasiana* current and future distribution models. 7](#_Toc149740190)

[Figure S1.10. *Pinus durangensis* current and future distribution models. 7](#_Toc149740191)

[Figure S1.11. *Pinus engelmannii* current and future distribution models. 8](#_Toc149740192)

[Figure S1.12. *Pinus herrerae* current and future distribution models. 8](#_Toc149740193)

[Figure S1.13. *Pinus leiophylla* current and future distribution models. 9](#_Toc149740194)

[Figure S1.14. *Pinus lumholtzii* current and future distribution models. 9](#_Toc149740195)

[Figure S1.15. *Pinus luzmariae* current and future distribution models. 10](#_Toc149740196)

[Figure S1.16. *Pinus maximinoi* current and future distribution models. 10](#_Toc149740197)

[Figure S1.17. *Pinus oocarpa* current and future distribution models. 11](#_Toc149740198)

[Figure S1.18. *Pinus strobiformis* current and future distribution models. 11](#_Toc149740199)

[Figure S1.19. *Pinus teocote* current and future distribution models. 12](#_Toc149740200)

[Figure S1.20. *Pinus yecorensis* current and future distribution models. 12](#_Toc149740201)

[Figure S1.21. Potential diversity of pines in the current scenario. 13](#_Toc149740202)

[Figure S1.22. Potential diversity of pines in the 2040 scenario. 13](#_Toc149740203)

[Figure S1.23. Potential diversity of pines in the 2060 scenario. 14](#_Toc149740204)

[Figure S1.24. Potential diversity of pines in the 2080 scenario. 14](#_Toc149740205)

[Figure S1.25. Potential diversity of pines in the 2100 scenario. 15](#_Toc149740206)

| 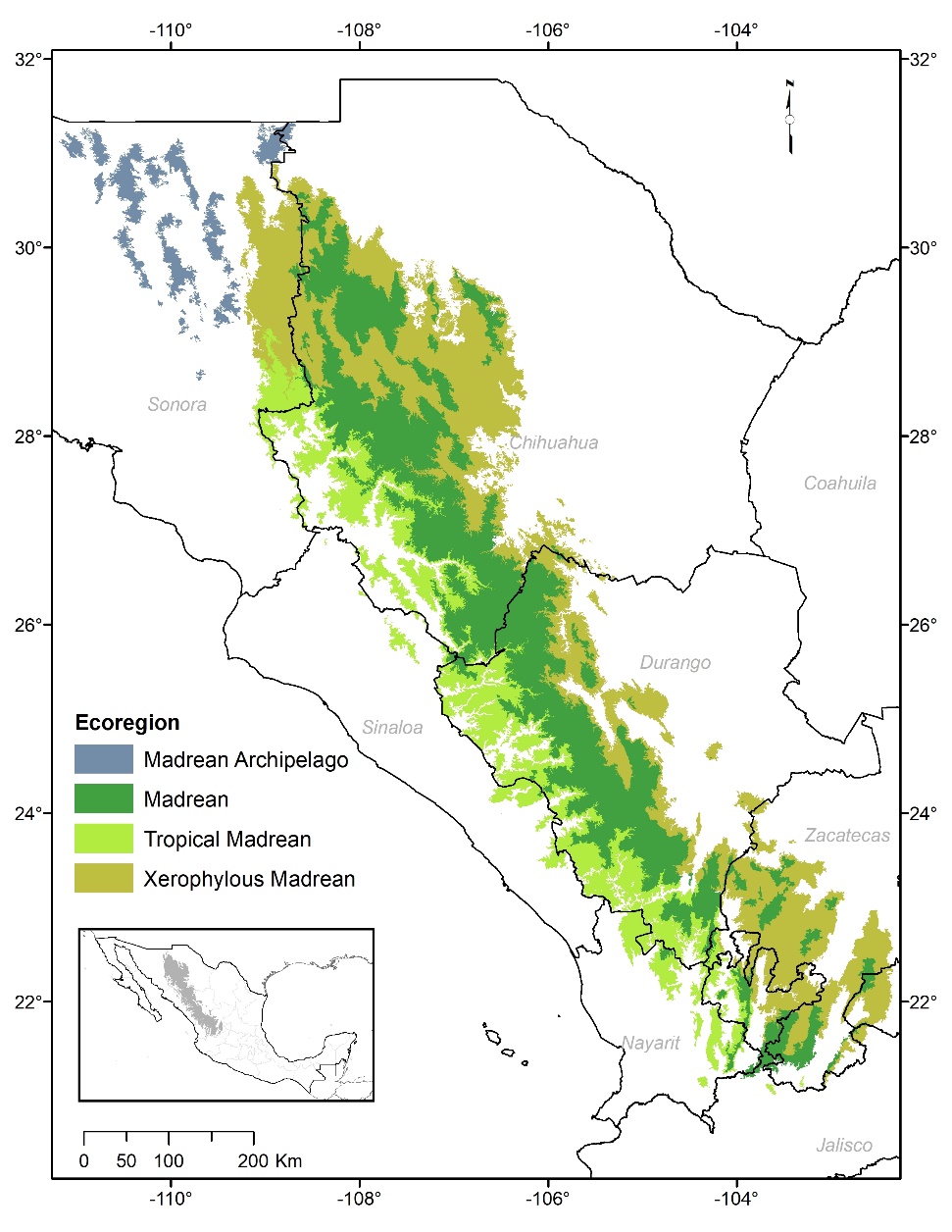 **Figure S1.1**. Madrean ecoregions of Sierra Madre Occidental. | 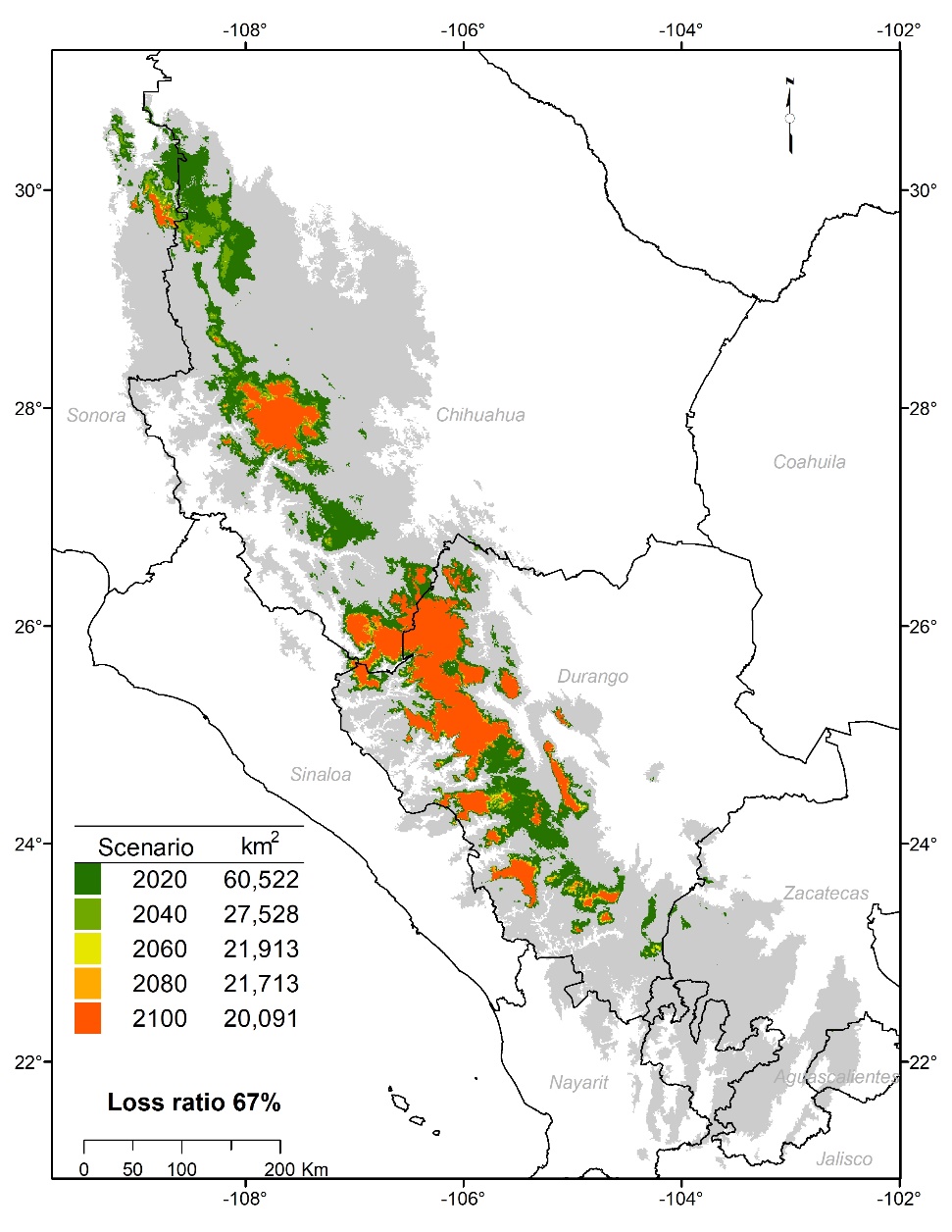 **Figure S1.2**. *Pinus arizonica* current and future distribution models. |
| --- | --- |
| 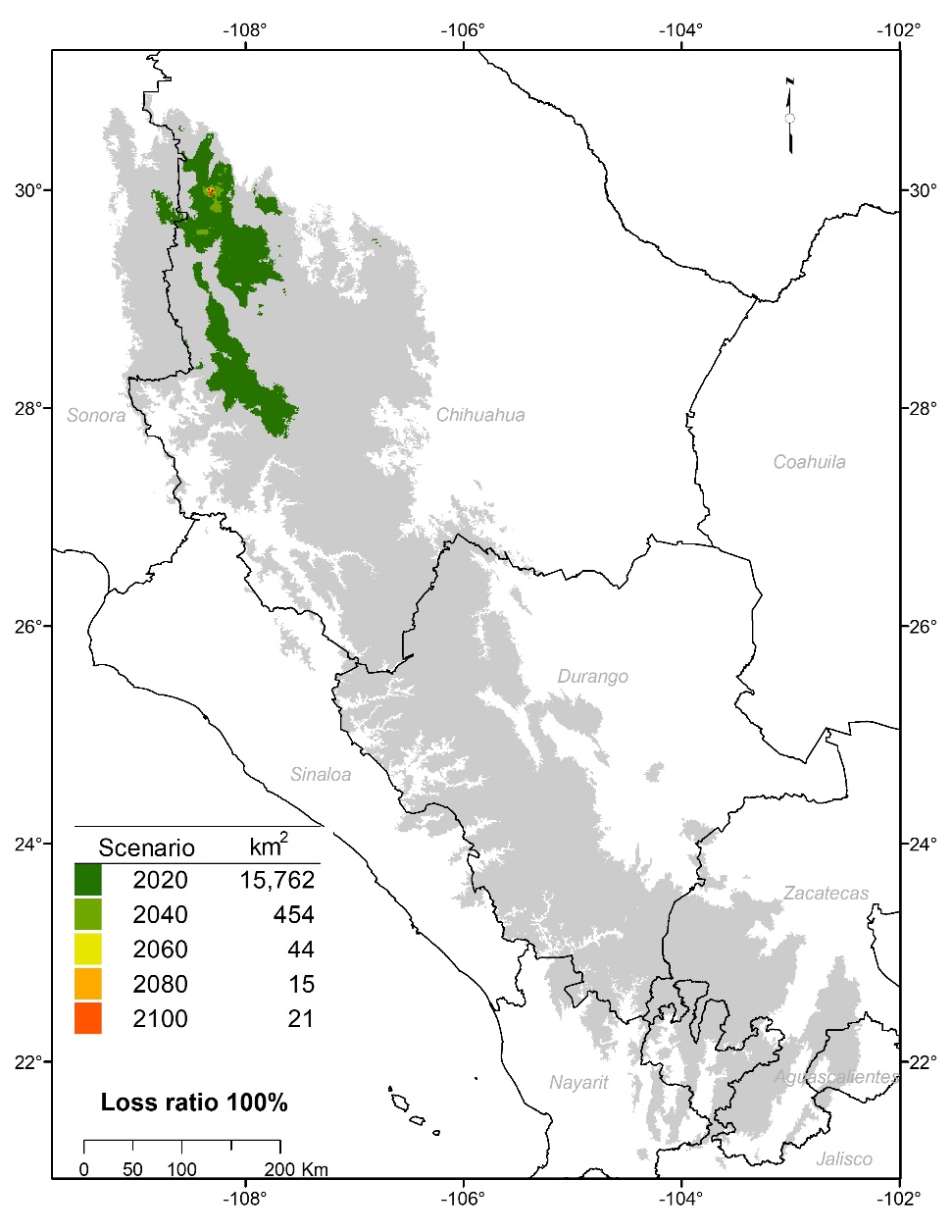 **Figure S1.3**. *Pinus brachyptera* current and future distribution models. | 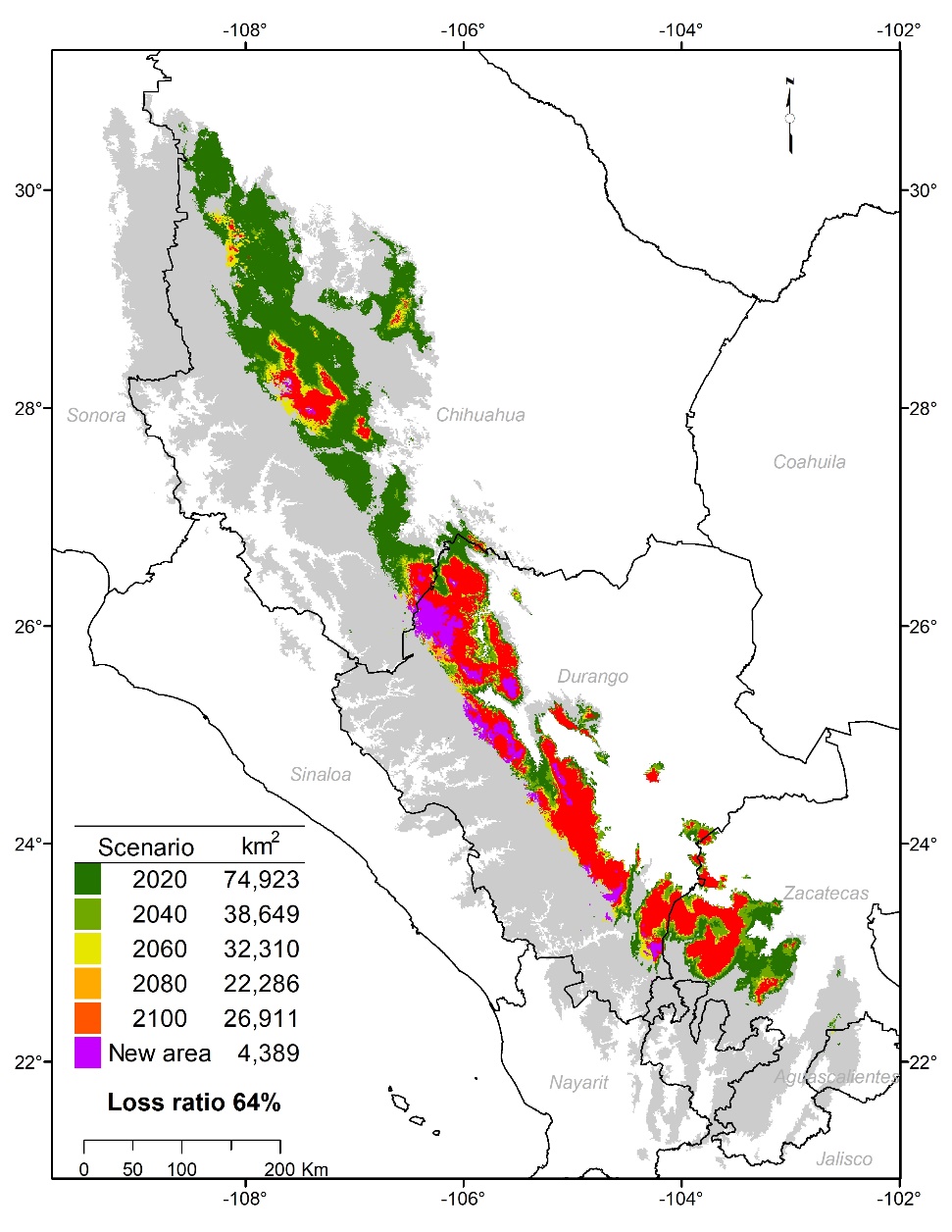 **Figure S1.4**. *Pinus cembroides* current and future distribution models. |
| 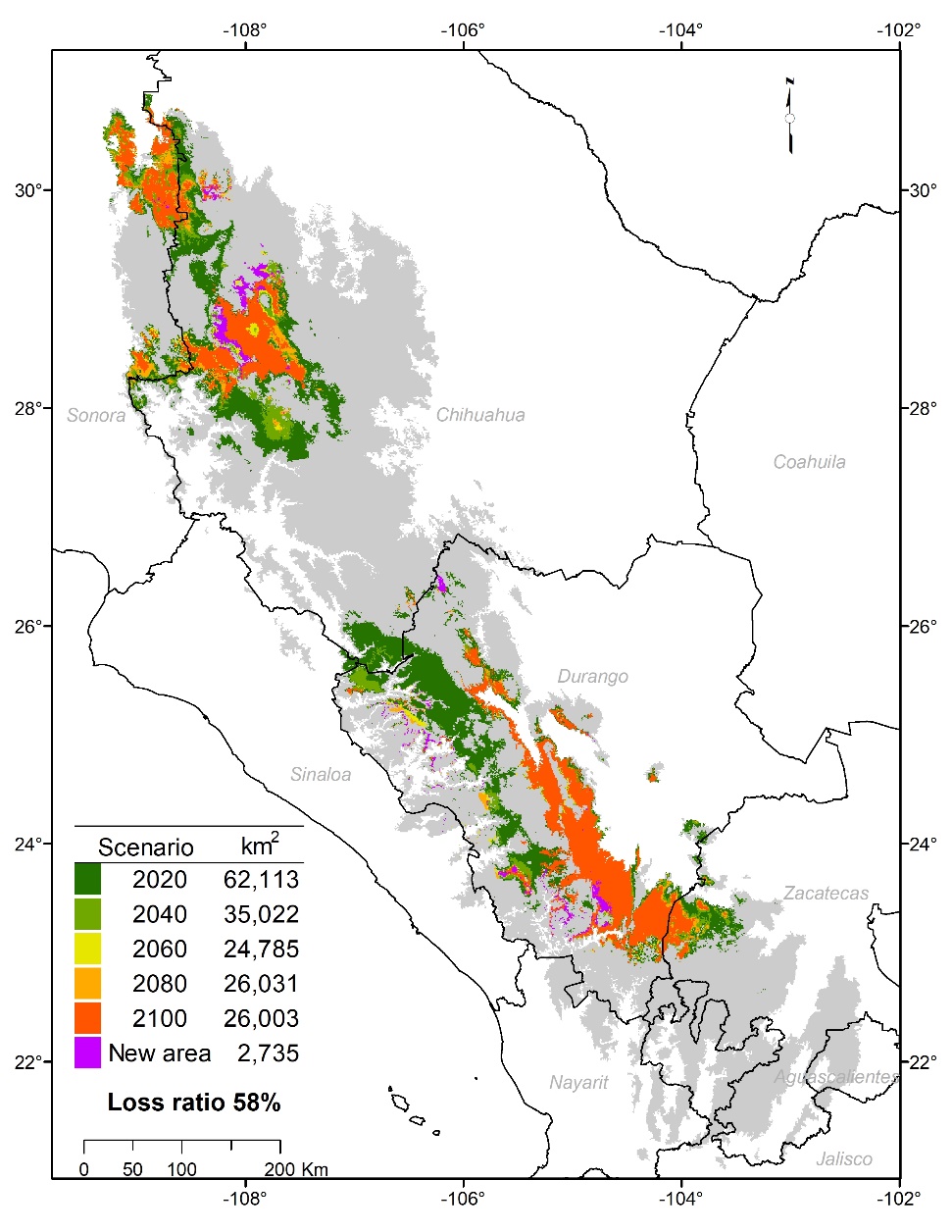 **Figure S1.5**. *Pinus chihuahuana* current and future distribution models. | 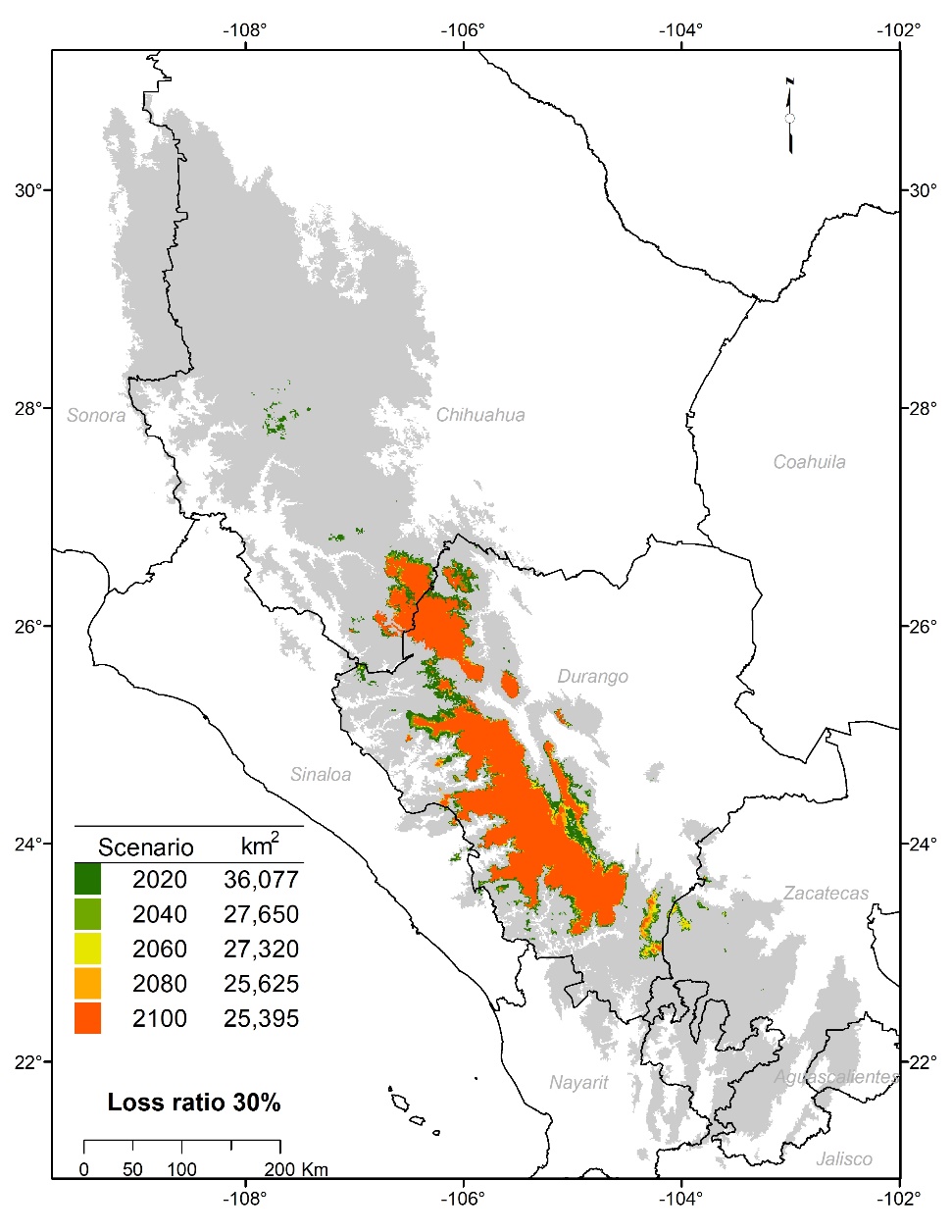 **Figure S1.6**. *Pinus cooperi* current and future distribution models. |
| 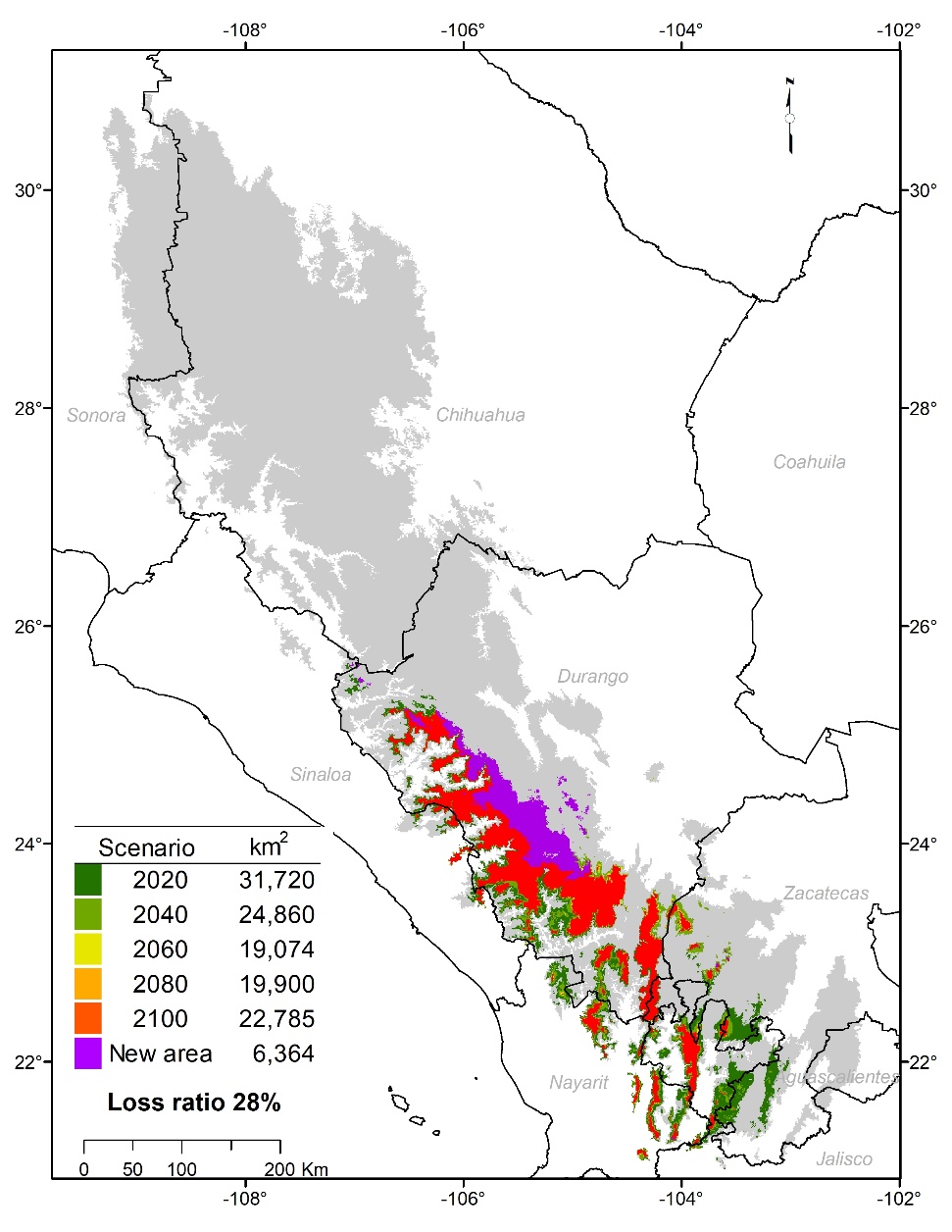 **Figure S1.7**. *Pinus devoniana* current and future distribution models. | 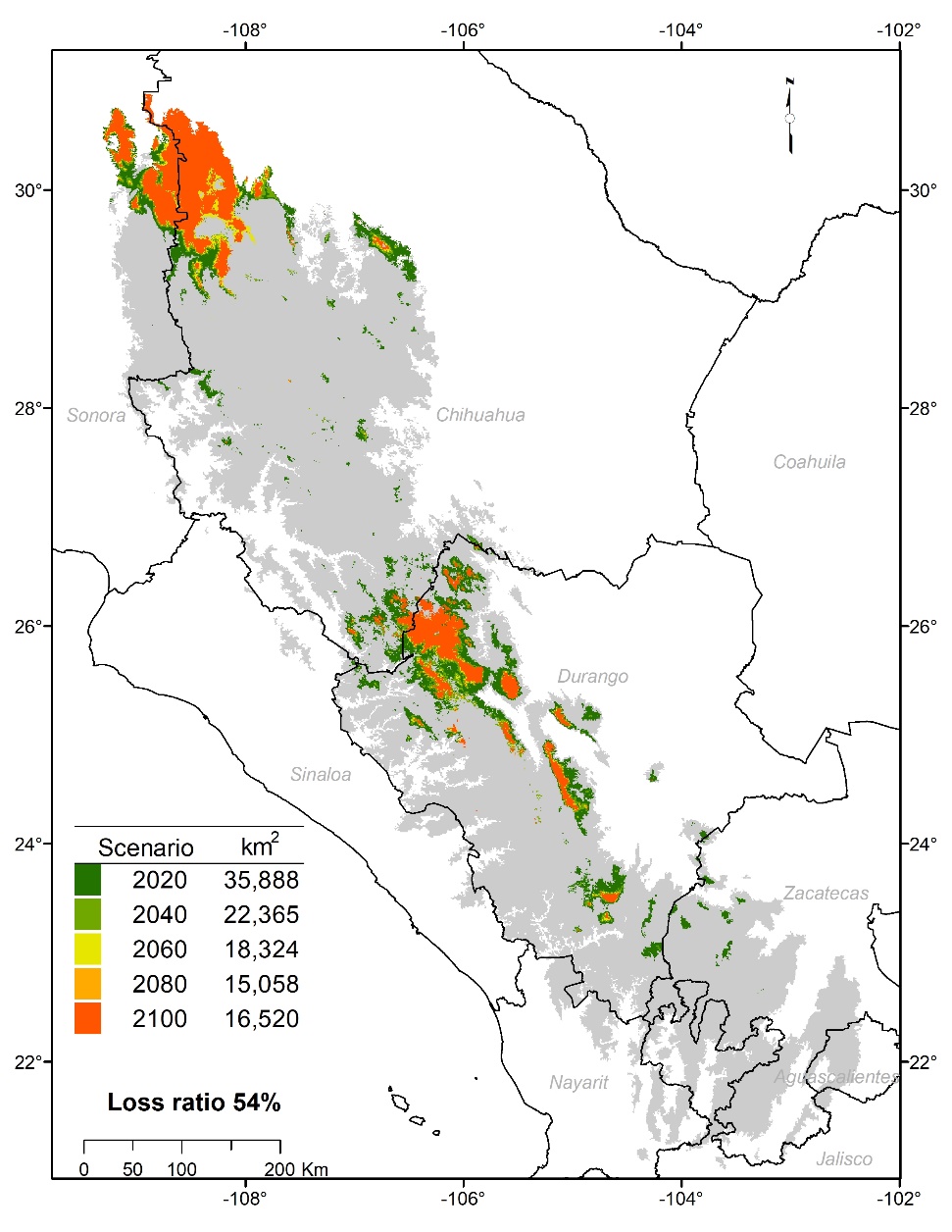 **Figure S1.8**. *Pinus discolor* current and future distribution models. |
| 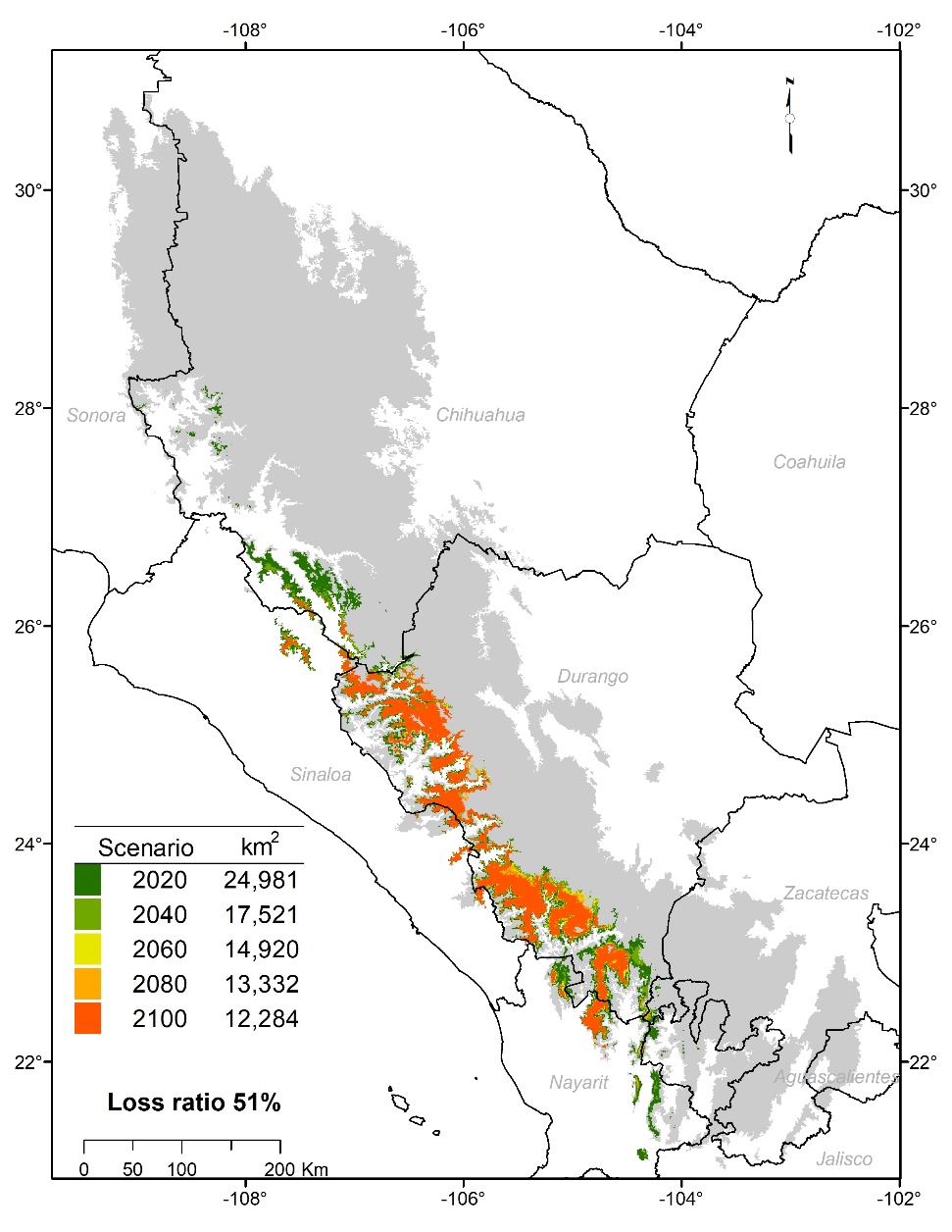 **Figure S1.9**. *Pinus douglasiana* current and future distribution models. | 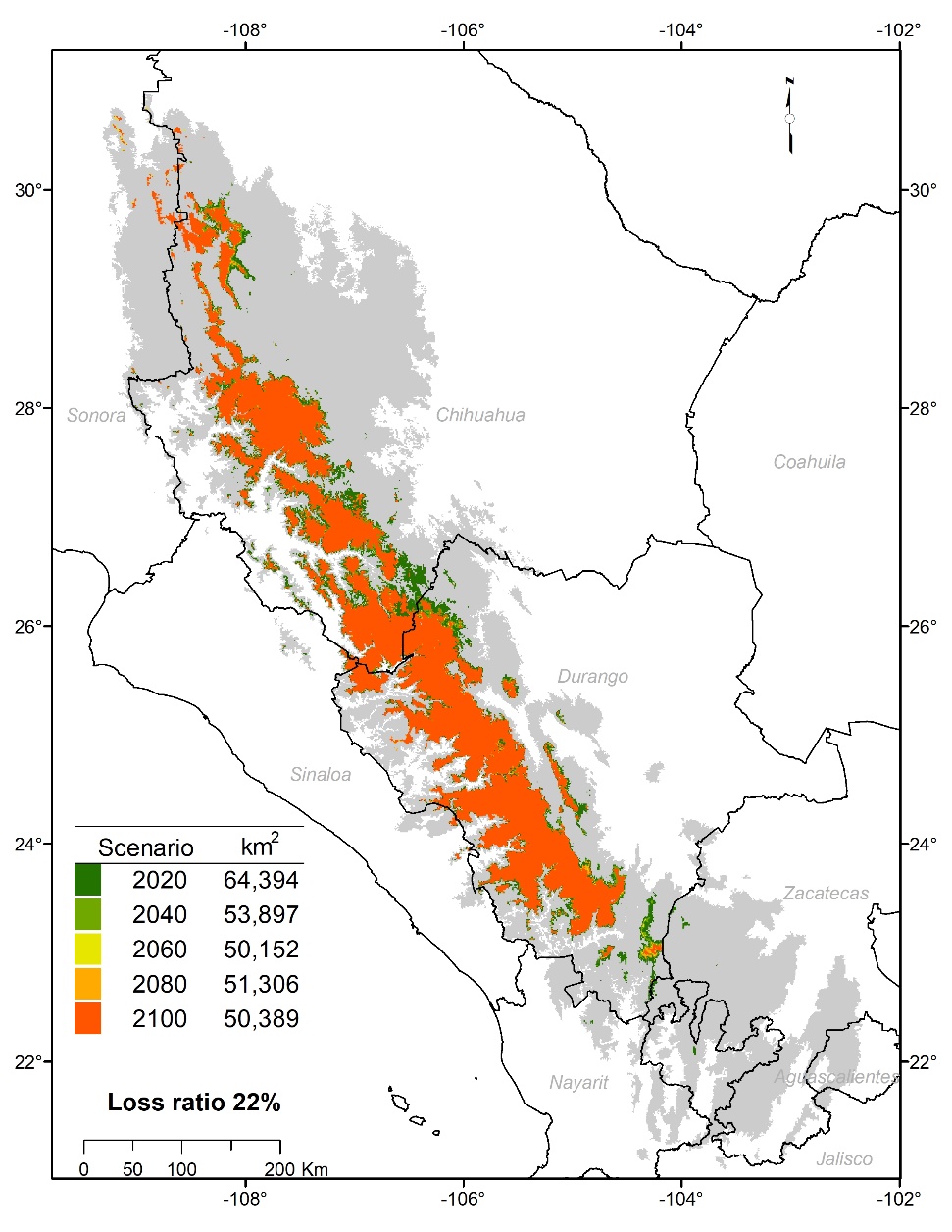 **Figure S1.10**. *Pinus durangensis* current and future distribution models. |
|  |  |
| 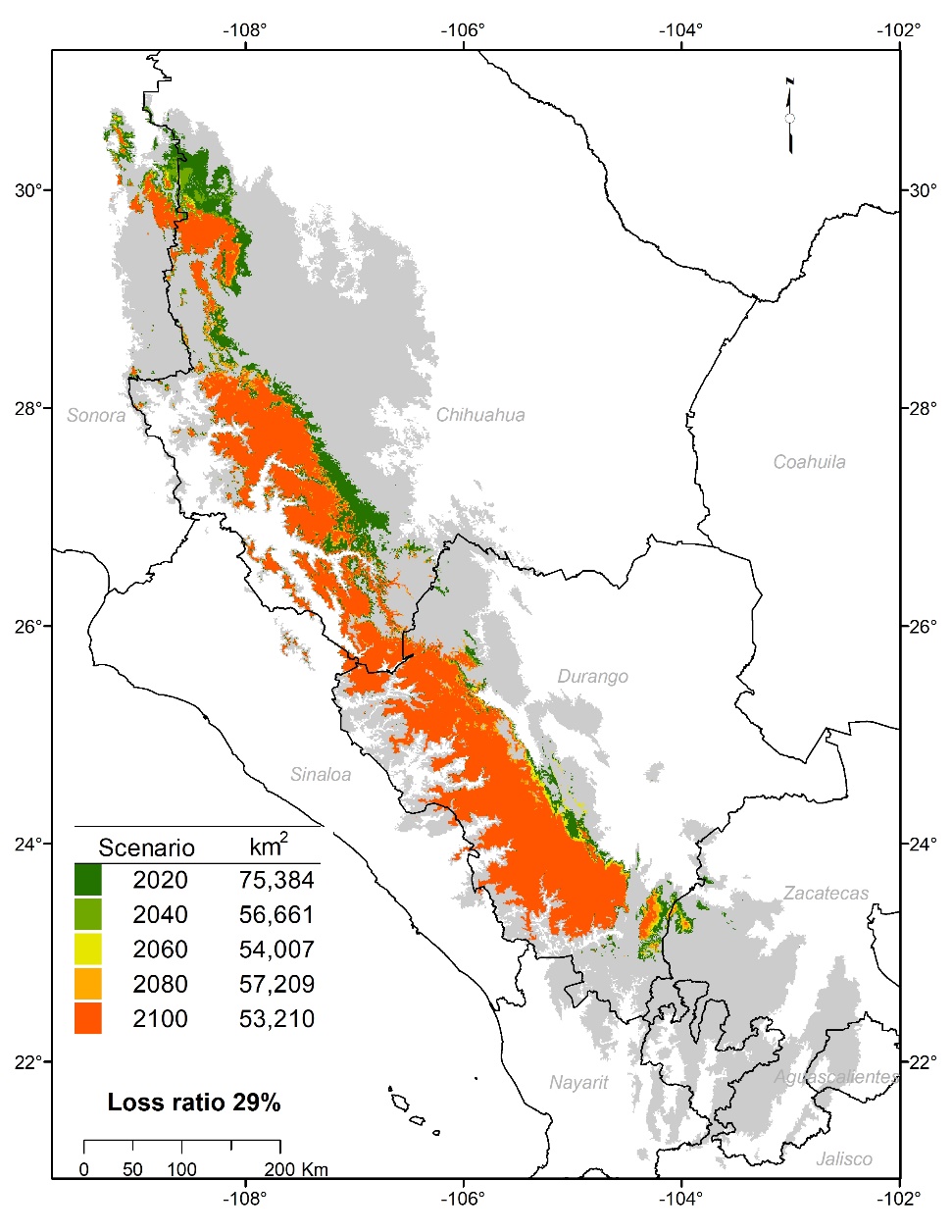 **Figure S1.11**. *Pinus engelmannii* current and future distribution models. | 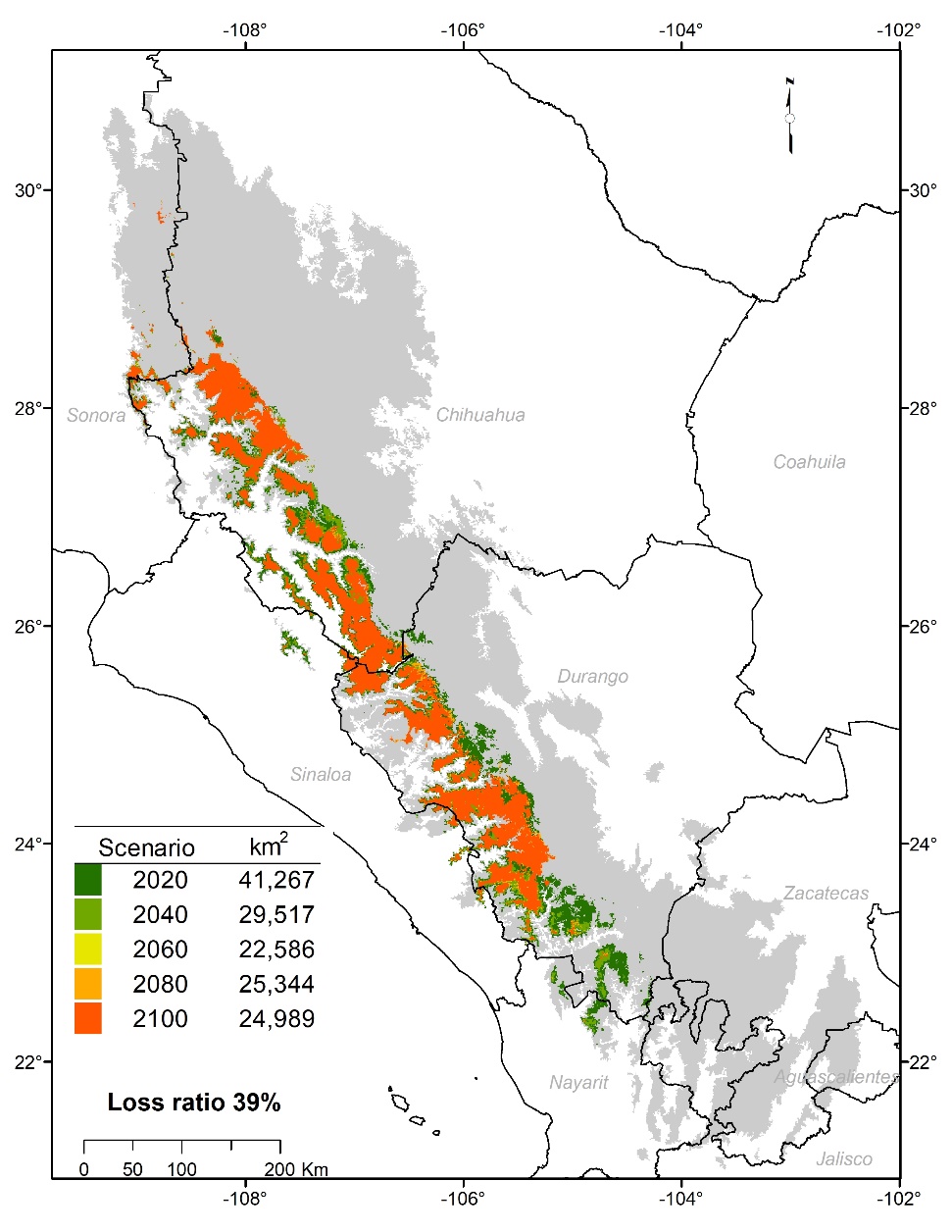 **Figure S1.12**. *Pinus herrerae* current and future distribution models. |
| 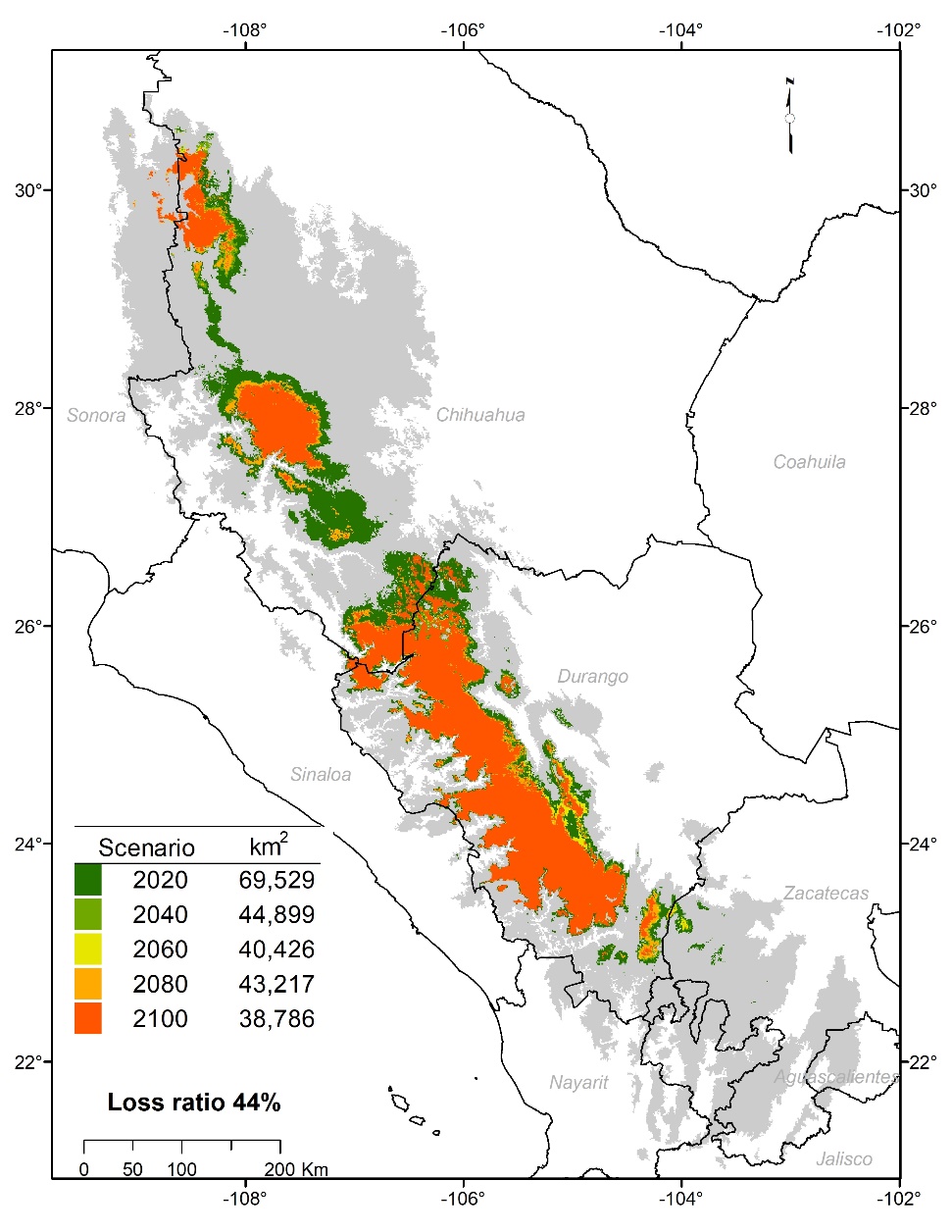 **Figure S1.13**. *Pinus leiophylla* current and future distribution models. | 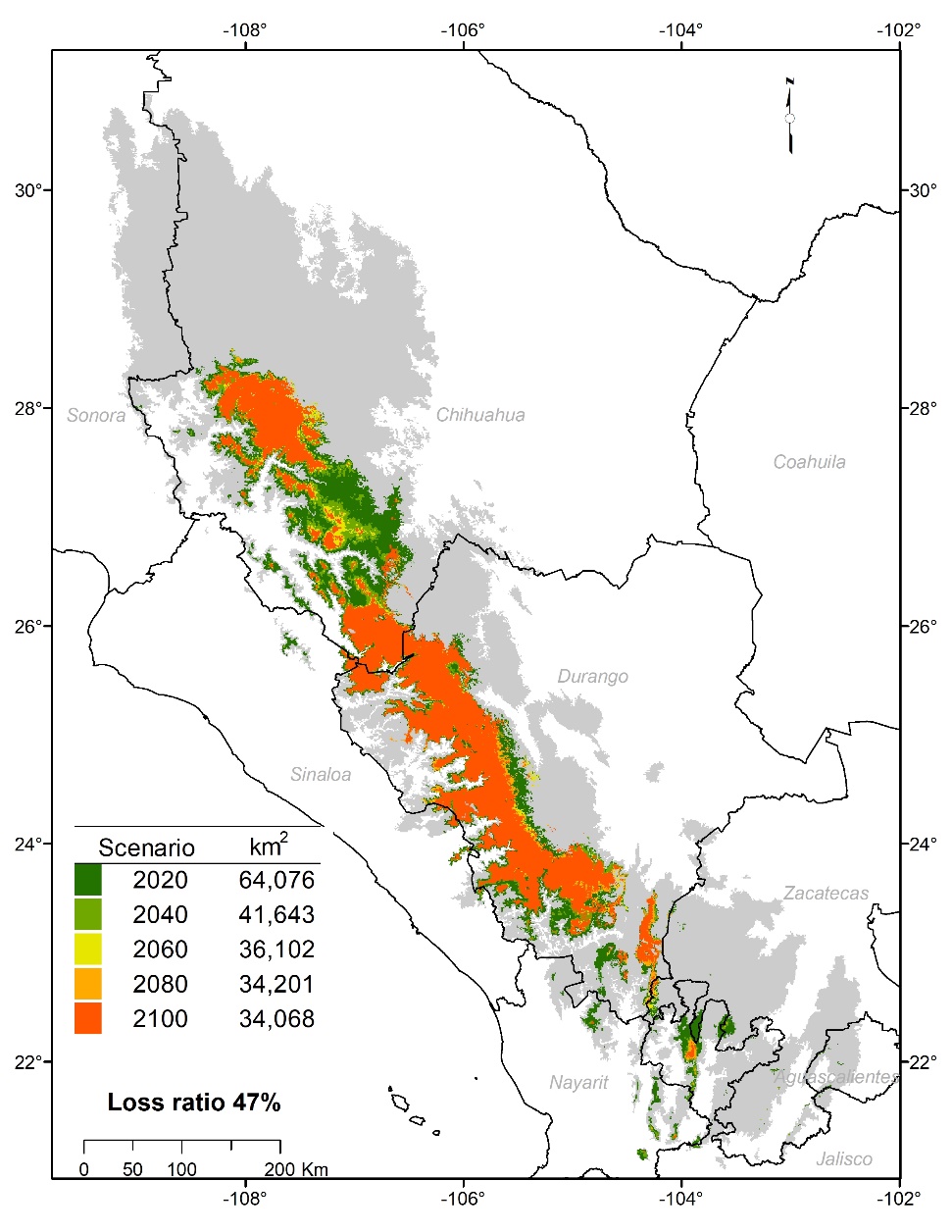 **Figure S1.14**. *Pinus lumholtzii* current and future distribution models. |
| 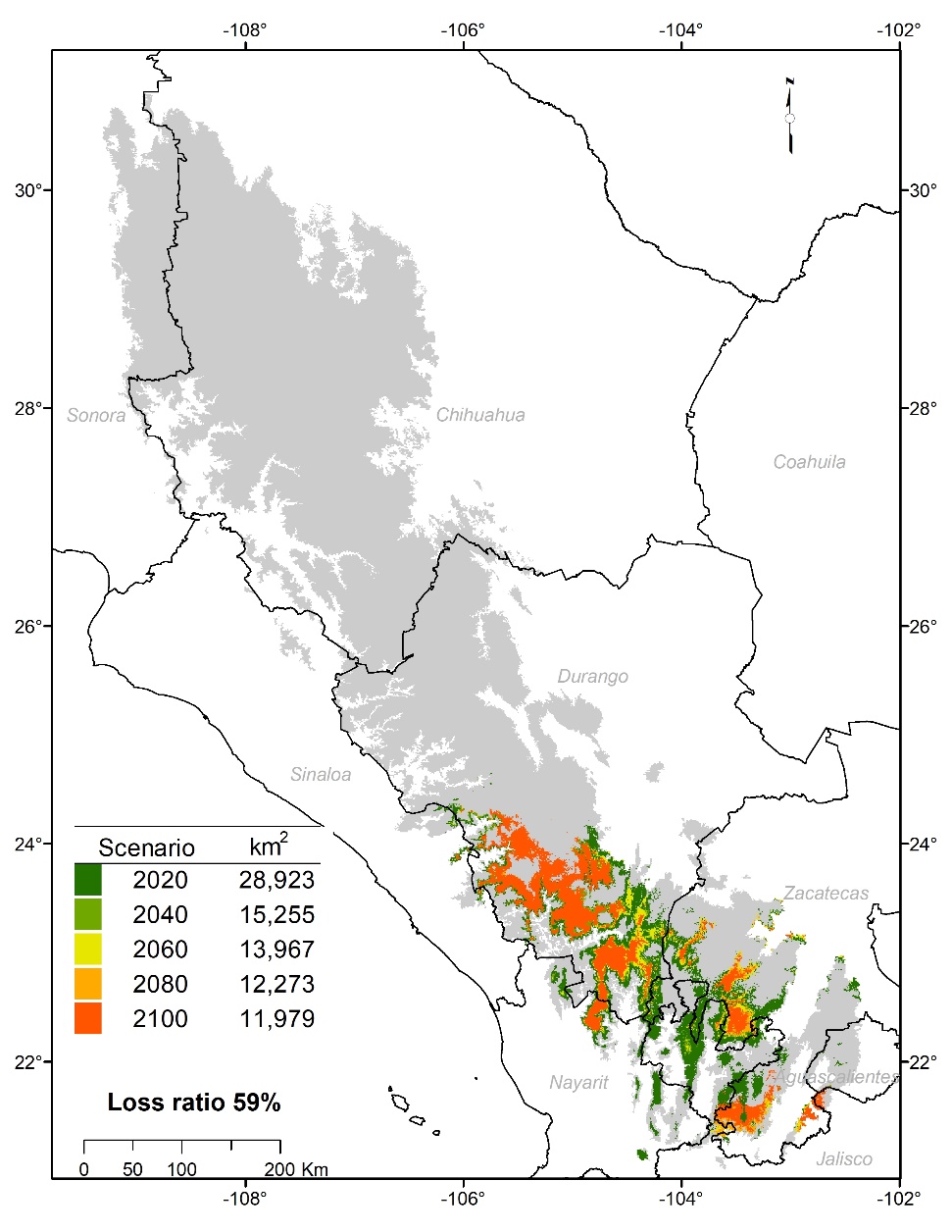 **Figure S1.15**. *Pinus luzmariae* current and future distribution models. | 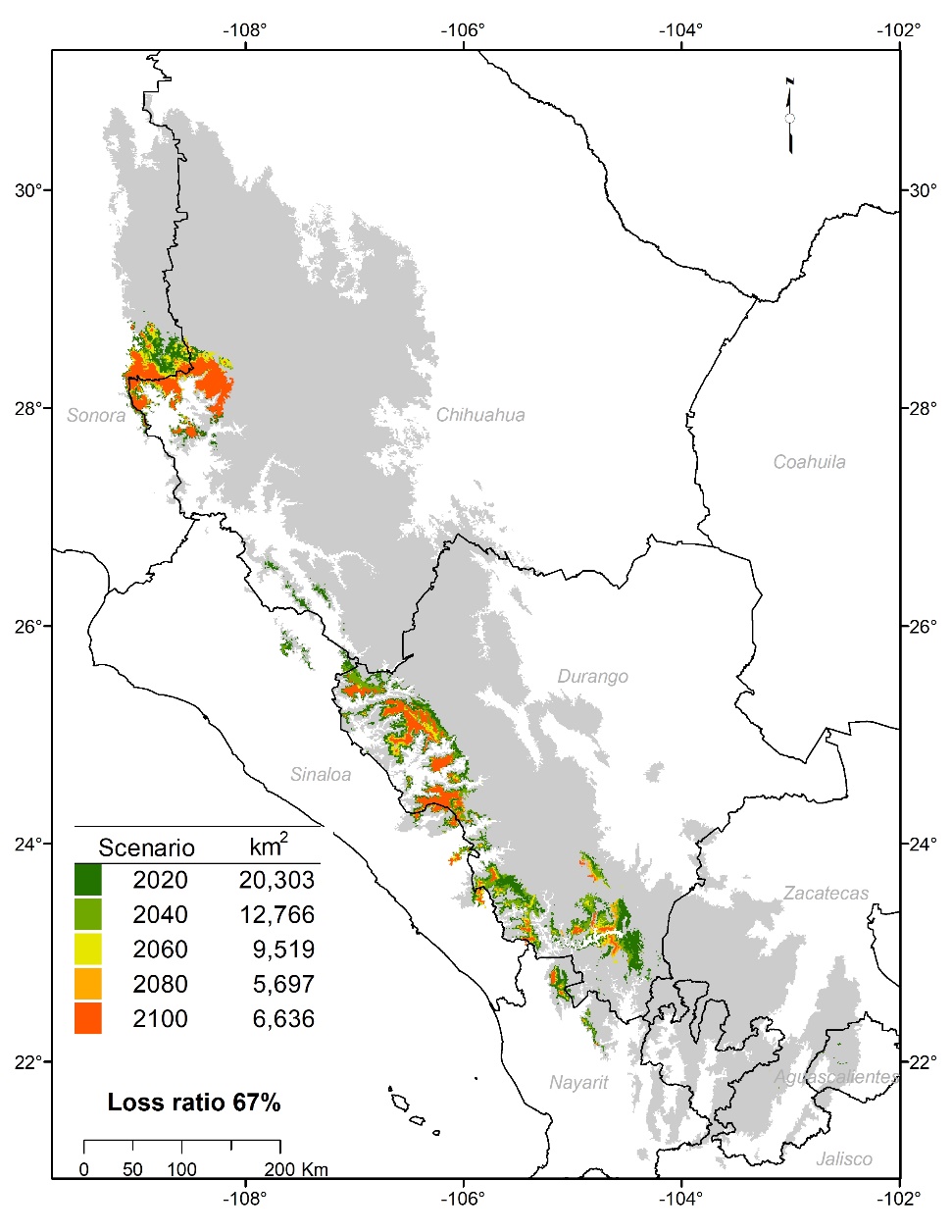 **Figure S1.16**. *Pinus maximinoi* current and future distribution models. |
| 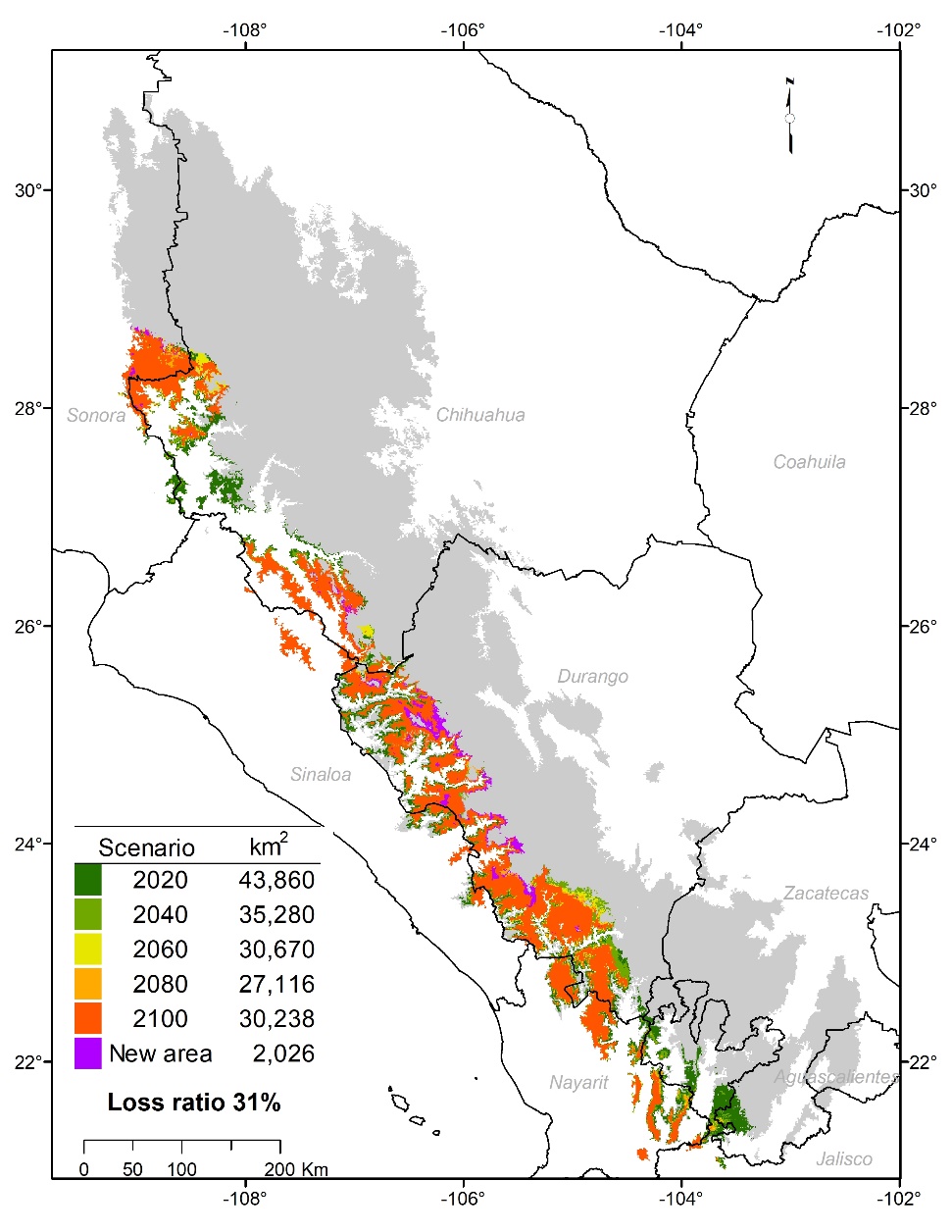 **Figure S1.17**. *Pinus oocarpa* current and future distribution models. | 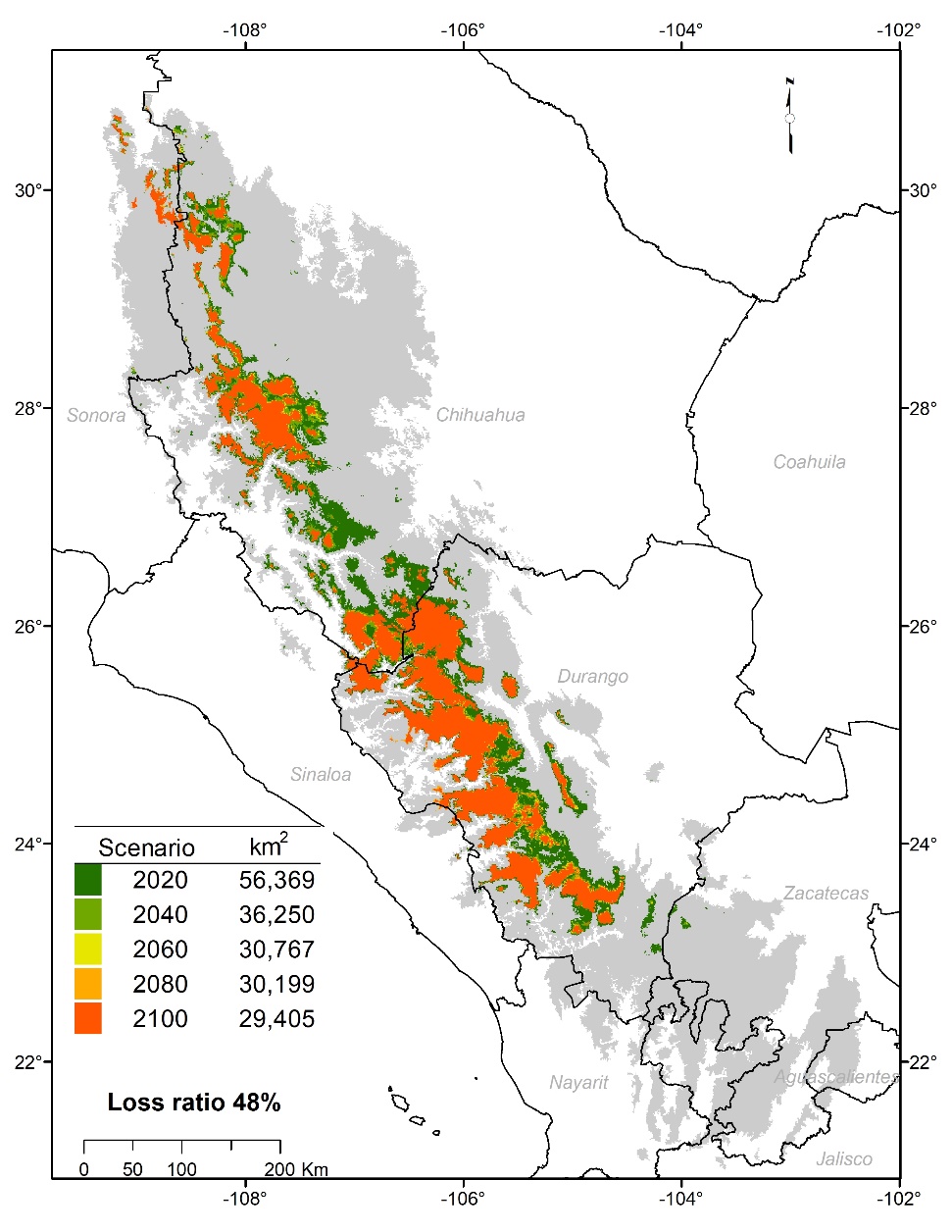 **Figure S1.18**. *Pinus strobiformis* current and future distribution models. |
| 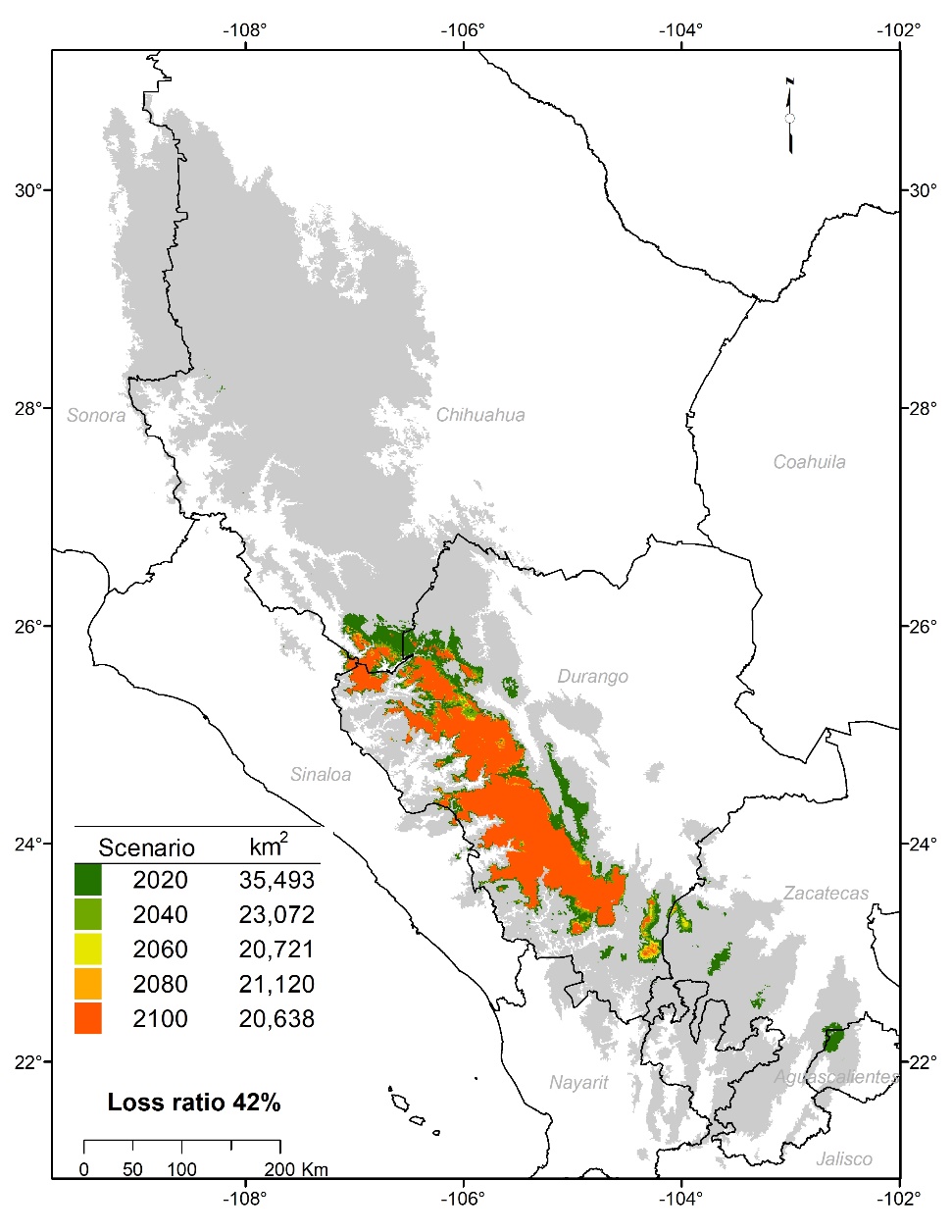 **Figure S1.19**. *Pinus teocote* current and future distribution models. | 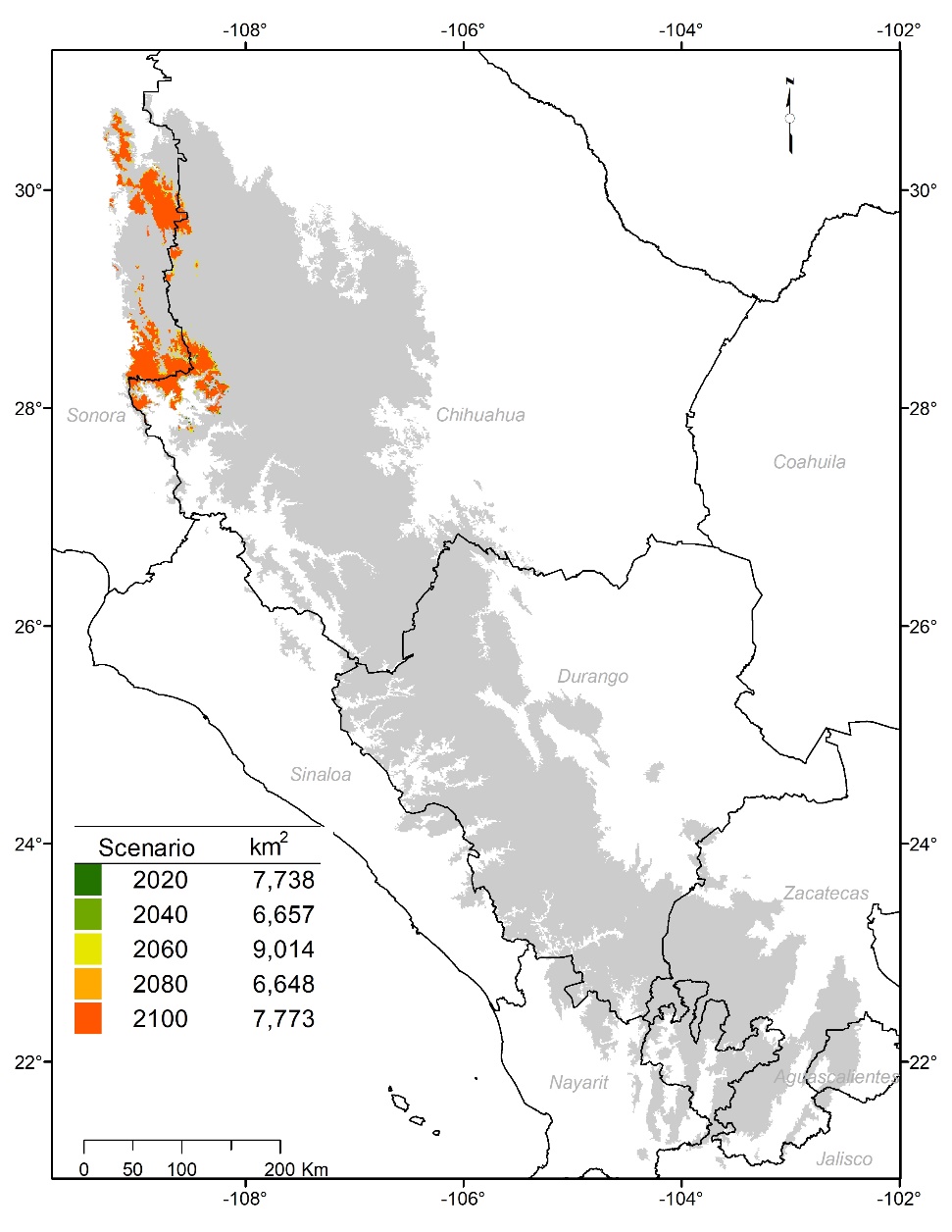 **Figure S1.20**. *Pinus yecorensis* current and future distribution models. |
| 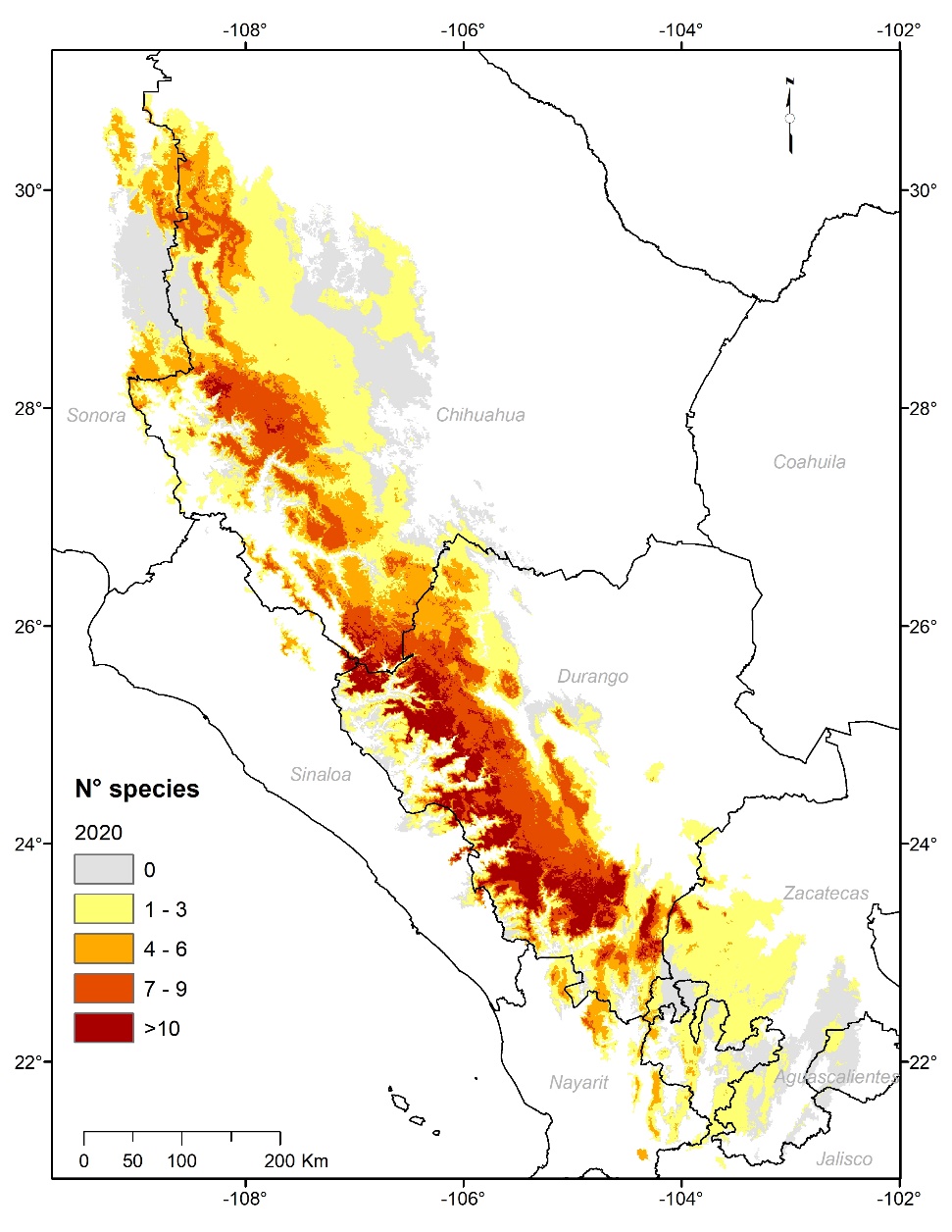 **Figure S1.21**. Potential diversity of pines in the current scenario. | 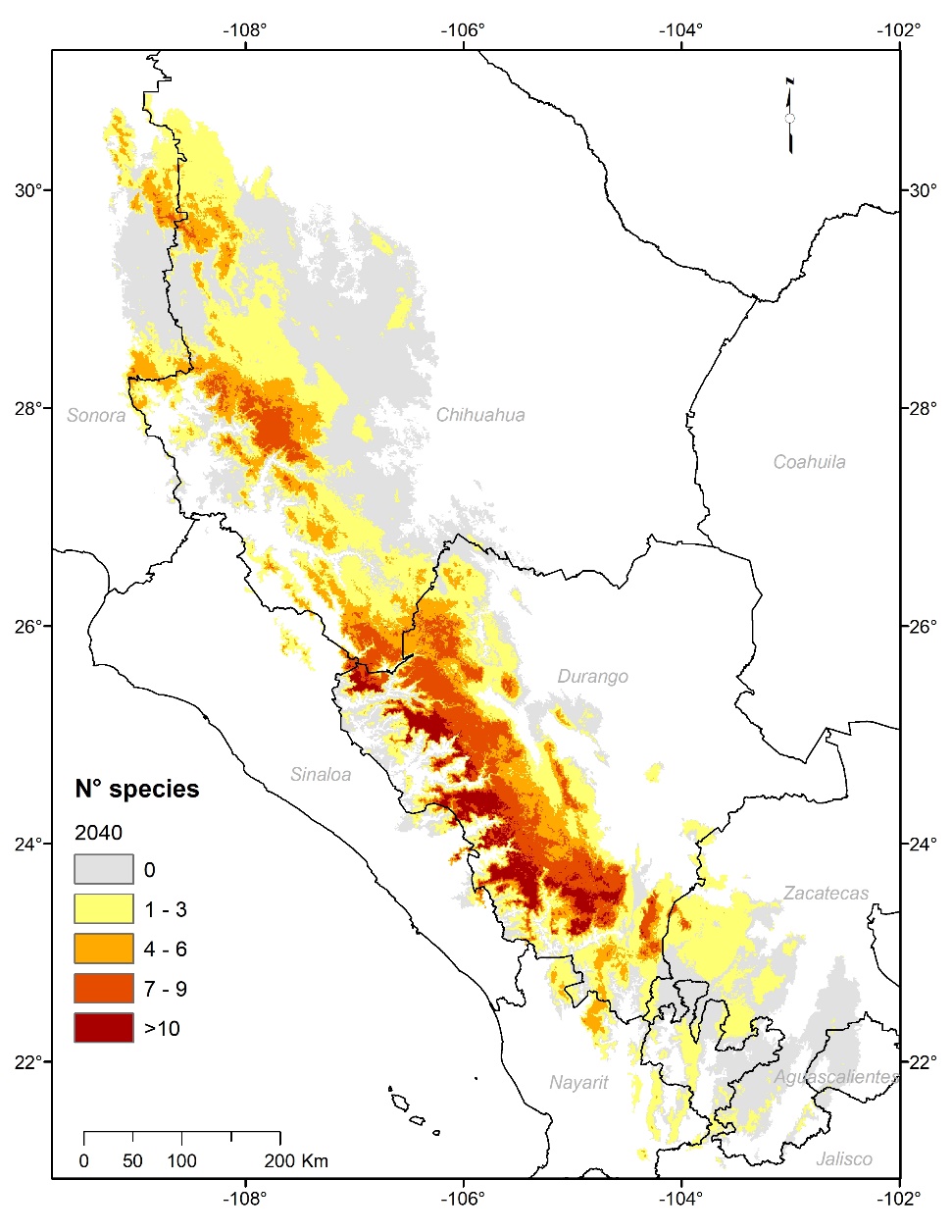 **Figure S1.22**. Potential diversity of pines in the 2040 scenario. |
| 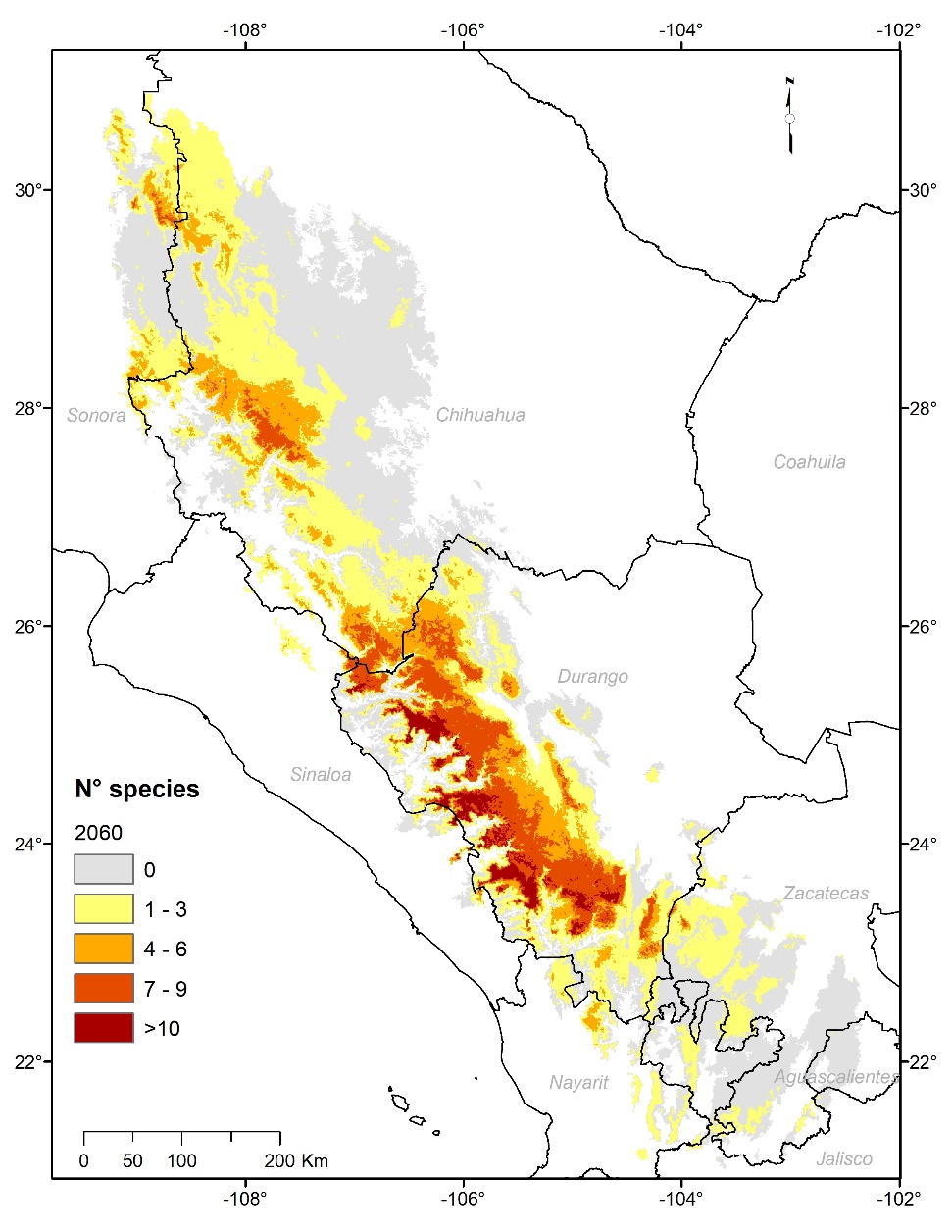 **Figure S1.23**. Potential diversity of pines in the 2060 scenario. | 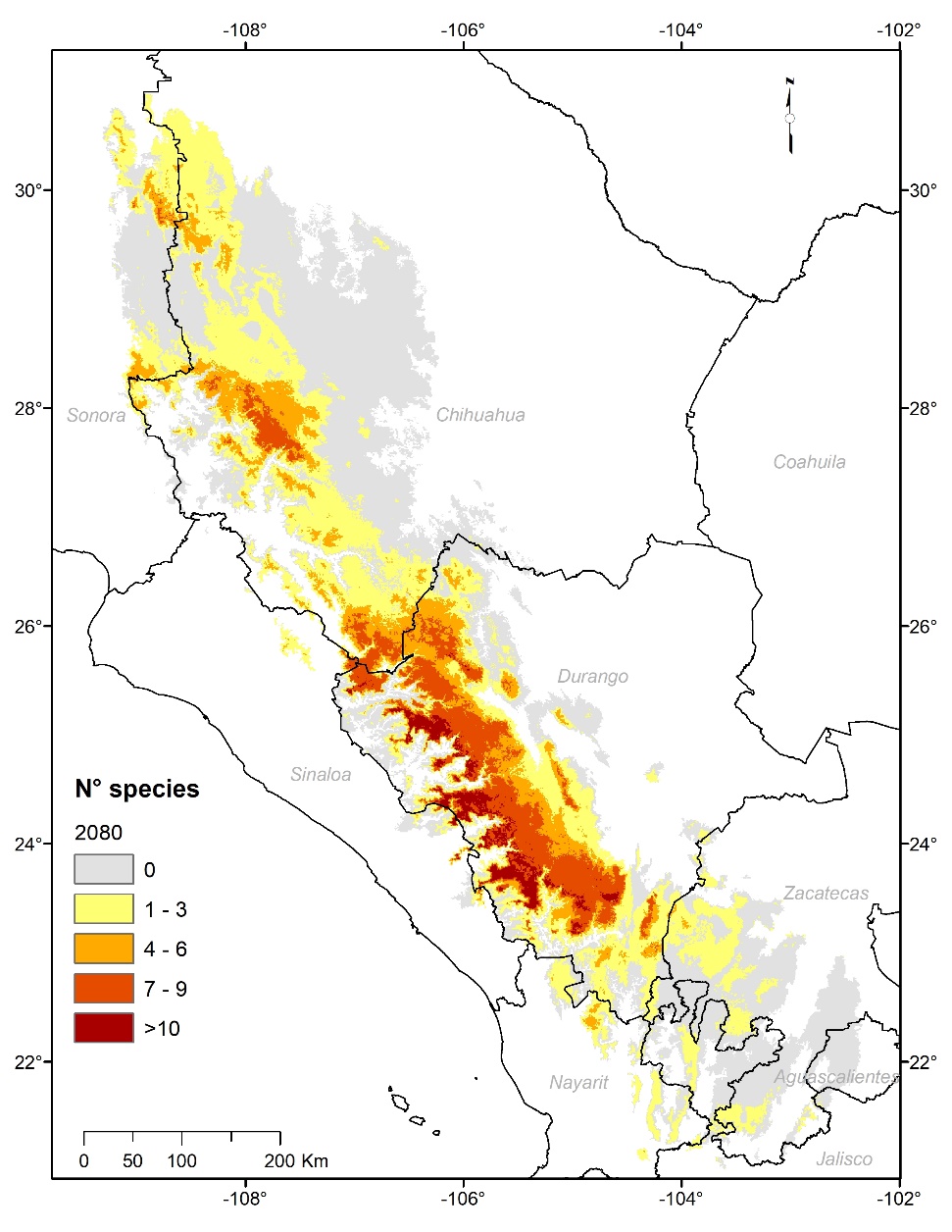 **Figure S1.24**. Potential diversity of pines in the 2080 scenario. |
| 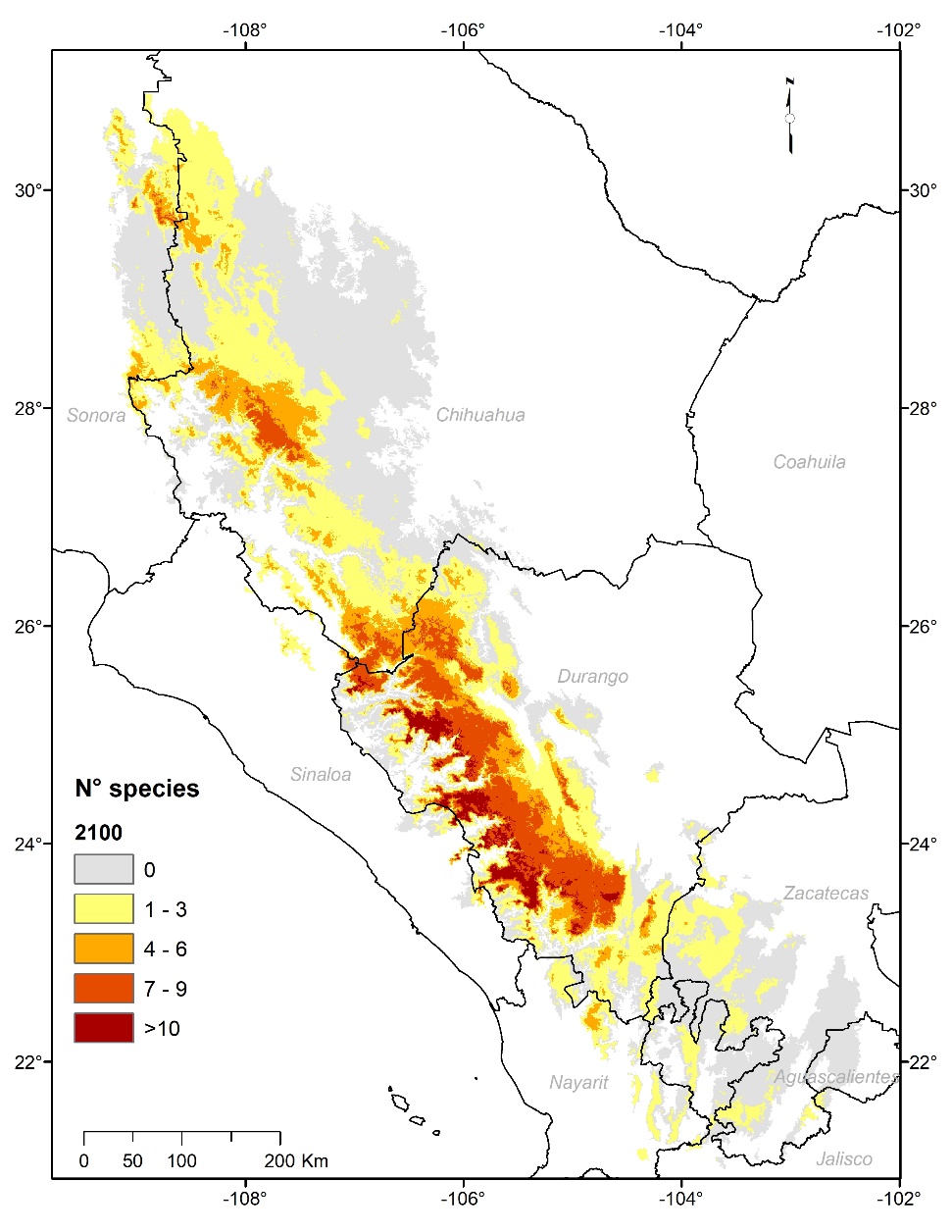 **Figure S1.25**. Potential diversity of pines in the 2100 scenario. |  |
