# Supplementary material for: Diversity Hotspots and Vulnerability of Pine Species in the Sierra Madre Occidental, Western Mexico
Source: Ecol Evol. 2025 Jul 9;15(7):e71743. doi: 10.1002/ece3.71743 (PMC12240594; doi:10.1002/ece3.71743)
Supplement: Supplementary file 1 — Appendix S1. [file ECE3-15-e71743-s001.zip › ece371743-sup-0003-Tables.docx]

**Supporting Information 2 for**

**Diversity hotspots and vulnerability of pine species in the Sierra Madre Occidental, western Mexico**

CONTENTS:

[Table S2.1. Pine species, records and ecoregion where each specie is distributed in Sierra Madre Occidental. 2](#_Toc149206633)

[Table S2.2. Excluded species that are cited in several research studies or have been reported in botanical collections. 3](#_Toc149206634)

[Table S2.3. Environmental variables at 30 arc-second resolutions from WorldClim 2.1 (Fick and Hijmans, 2017). 5](#_Toc149206635)

[Table S2.4. ODMAP (Overview, Data, Model, Assessment and Prediction) protocol of the modelling process according with Zurell *et al*. (2020). 6](#_Toc149206636)

[Table S2.5. Variables used in each specie model after removal of collinearity and correlation. 9](#_Toc149206637)

# **Table S2.1**. Pine species, records and ecoregion where each species is distributed in the Sierra Madre Occidental.

| Species | Records | Ecoregion | | |
| --- | --- | --- | --- | --- |
|  |  | M | TM | XM |
| *Pinus arizonica* | 593 | X |  |  |
| *P. brachyptera* | 21 | X |  |  |
| *P. cembroides* | 261 |  |  | X |
| *P. chihuahuana* | 326 |  | X | X |
| *P. cooperi* | 361 | X |  |  |
| *P. devoniana* | 51 | X | X |  |
| *P. discolor* | 35 | X |  | X |
| *P. douglasiana* | 67 |  | X |  |
| *P. durangensis* | 1127 | X | X |  |
| *P. engelmannii* | 682 | X | X | X |
| *P. herrerae* | 294 | X | X |  |
| *P. leiophylla* | 758 | X |  |  |
| *P. lumholtzii* | 598 | X | X |  |
| *P. luzmariae* | 35 |  | X |  |
| *P. maximinoi* | 44 |  | X |  |
| *P. oocarpa* | 60 |  | X |  |
| *P. strobiformis* | 1058 | X | X |  |
| *P. teocote* | 605 | X |  |  |
| *P. yecorensis* | 44 |  | X |  |

(M= Madrean, TM= Tropical Madrean and XM = Xerophylous Madrean region)

# Table S2.2. Excluded species or names in synonymy that have been cited in several research studies or have been reported in botanical collections.

| Species | Argument for exclusion |
| --- | --- |
| *P. ayacahuite* | Distributed from central Mexico to El Salvador. (Farjon & Styles, 1997) |
| *P. ayacahuite* var*. brachyptera* | Synonym for *P. strobiformis* (Farjon & Styles, 1997) |
| *P. caribaea* | All varieties are distributed in the Caribean region. Only the var. *hondurensis* occurs in Mexico (southern Quintana Roo) |
| *P. cembroides* var*. bicolor* | Synonym for *P. discolor* (Price *et al*., 1998) |
| *P. engelmannii* var*. blancoi* | Unrecognized variety (Perry, 1991; Farjon & Styles, 1997) |
| *P. halepensis* | Mediterranean native species used as an ornamental and for restoration in Mexico (Venegas-López, 2016) |
| *P. jeffreyi* | Species of western USA, its southern limit reaches northern Baja California, Mexico (Farjon *et al.*, 1997) |
| *P. lawsonii* | Known from the Transmexican Volcanic Belt and the Sierra Madre del Sur (Farjon *et al.*, 1997) |
| *P. leiophylla var. chihuahuana* | Synonym for P*. chihuahuana* (Almaráz-Abarca *et al.,* 2006; Gernandt & Pérez de la Rosa, 2014) |
| *P. lutea* | Name not accepted due to homonymy. Specimens identified under this name are recognized as *P. cooperi* (Farjon & Styles, 1997) |
| *P. martinezii* | Species restricted to Michoacán, Mex. (Contreras-Bailón, 2021) |
| *P. michoacana* | Synonym for *P. devoniana* (Farjon *et al.*, 1997) |
| *P. oocarpa var. microphylla* | Synonym for *P. praetermissa* (Farjon & Styles, 1997) |
| *P. pinea* | Species from the Iberian Peninsula and Mediterranean regions, planted in Mexico as an ornamental (Abad-Viñas *et al.,* 2016) |
| *P. ponderosa* | Species distributed in Canada and the United States (Willard *et al*., 2021).  Mexican specimens identified under this name become *P. brachyptera*. |
| *P. ponderosa var. arizonica* | Synonym for *P. arizonica* (Farjon *et al.*, 1997) |
| *P. ponderosa var. brachyptera* | Synonym for *P. brachyptera* (Willard *et al*., 2021). |
| *P. ponderosa var. scopolorum* | Synonym for *P. scopolorum*, which is from United States. |
| *P. pringlei* | From the Transmexican Volcanic Belt and Sierra Madre del Sur (Farjon *et al.*, 1997) |
| *P. pseudostrobus* | Distributed from central Mexico to Honduras. (Farjon & Styles, 1997) |
| *P. stormiae* | Synonym for *P. arizonica* var. *stormiae*, which occurs in the Sierra Madre Oriental (Williard, 2021) |
| *P. sylvestris* | Species from Europe |
| *P. tenuifolia* | Synonym for *P. maximinoi* (Farjon *et al.*, 1997) |

# Table S2.3. Environmental variables at 30 arc-second resolutions from WorldClim 2.1 (Fick and Hijmans, 2017).

| Category | Variable | Unit |
| --- | --- | --- |
| Bioclimatic | Bio1 = annual mean temperature | °C |
|  | Bio2 = diurnal mean range (mean of monthly (max temp–min temp)) | °C |
|  | Bio3 = isothermality (BIO2/BIO7) (x 100) | °C |
|  | Bio4 = temperature seasonality | % |
|  | Bio5 = maximum temperature of the warmest  month | °C |
|  | Bio6 = minimum temperature of the coldest month | °C |
|  | Bio7 = annual temperature range (Bio5-Bio6) | °C |
|  | Bio8 = mean temperature of the wettest quarter | °C |
|  | Bio9 = mean temperature of the driest quarter | °C |
|  | Bio10 = mean temperature of the warmest quarter | °C |
|  | Bio11 = mean temperature of the coldest quarter | °C |
|  | Bio12 = annual precipitation | mm |
|  | Bio13 = precipitation of the wettest month | mm |
|  | Bio14 = precipitation of the driest month | mm |
|  | Bio15 = precipitation seasonality (coefficient of variation) | % |
|  | Bio16 = precipitation of the wettest quarter | mm |
|  | Bio17 = precipitation of the driest quarter | mm |
|  | Bio18 = precipitation of the warmest quarter | mm |
|  | Bio19 = precipitation of the coldest quarter | mm |
|  | Smax = maximum solar radiation | KJ m^2^ |
|  | Smean = solar radiation mean | KJ m^2^ |
|  | Smin = minimum solar radiation | KJ m^2^ |
|  | Srang = solar radiation range | KJ m^2^ |
|  | Sstd = solar radiation standard deviation | KJ m^2^ |
|  | Vmax = max water vapor pressure | kPa |
|  | Vmean = water vapor pressure mean | kPa |
|  | Vmin = minimum water vapor pressure | kPa |
|  | Vrang = water vapor pressure range | kPa |
|  | Vstd = water vapor pressure standard deviation | kPa |
| Topographic | Aspect | degrees |
|  | Slope | degrees |
|  | Altitude | m asl |

# **Table S2.4.** ODMAP (Overview, Data, Model, Assessment and Prediction) protocol of the modelling process according with Zurell *et al*. (2020).

| **Section** | **Element** |
| --- | --- |
| ***Overview*** | |
| Authorship | Redacted |
| Model objective | Predict current and future distribution of target species |
| Taxon | 19 pine species |
| Location | Sierra Madre Occidental, México. |
| Scale of analysis | Spatial extent: Latitude 21.01-30.89 N, Longitude 102.33-109.31 W.  Spatial resolution: 30 arc sec (~1 km^2^)  Temporal resolution and extent: Data collections since 1980  Type of boundary: Natural |
| Biodiversity data overview | Observation type: curated herbarium records, permanent forest monitoring sites and citizen science observations. |
| Type of predictors | Bioclimatic and topographic |
| Conceptual model/hypotheses | Each species is distributed under particular climatic and topographic conditions, and these will modify their future distribution according to the most significant environmental variables shifts for each taxon. |
| Assumptions | We assume species are in equilibrium with their environment and key explanatory variables were incorporated in the model |
| SDM algorithms | Maxent |
| Model workflow | Highly correlated variables were detected and eliminated using the variance inflation factor (VIF). A Spearman correlation analysis was applied to the remaining variables (ρ > 0.70) to identify the variables to be used in each model building. Models were run with 10 cross validation replicates and 500 interations. Maximum trainig sensitiviy plus specificity was used as threshold setting. and the output was of the logistic type. |
| Software, codes and data | RStudio (‘vifcor’ function of the ‘usdm’ package)  Maxent 3.4.4 |
| ***Data*** | |
| Biodiversity data | Taxon names: *Pinus arizonica*, *P. brachyptera*, *P. cembroides*, *P. chihuahuana P. cooperi, P. devoniana, P. discolor, P. durangensis, P. engelmannii, P. herrerae, P. leiophylla, P. lumholtzii, P. luzmariae, P. maximinoi, P. oocarpa, P. strobiformis, P. teocote, P. yecorensis* var. *yecorensis*  Taxonomic reference system: Gernandt & Pérez de la Rosa (2014).  Ecological level: species  Sampling design: Herbarium records, permanent forest monitoring sites and citizen science observations with separations of at least 1 km radius.  Region mask: Madrean regions of the Sierra Madre Occidental according González-Elizondo *et al*., 2013.  Sample size per taxon: see Table S1  Data cleaning: records with incomplete or null coordinates, insufficient curatorial degree and proximity of another data less than 1km radius were eliminated.  Absence data: NA  Background data: All records are from template region.  Errors and biases: It is possible that some taxa names include hybrids or undescribed species or varieties, e.g., in *P. durangensis.* These records here are considered *sensu lato*. |
| Data partitioning | Selection of training data: 10 folds  Validation data: cross validation  Test data: NA |
| Predictors variables | Predictor variables: 32 (see Table S.2)  Data sources: layers were downloaded from Worldclim 2.1 (https://www.worldclim.org/data/worldclim21.html)  Spatial resolution: 30 arc-second  Map projection: GCS WGS84  Data processing and scaling: the mean, minimum, maximum, range and standard deviation for solar radiation and water vapor pressure were generated using the 'cell statistic' tool of Arcmap 10.8 from the monthly layers downloaded from Worldclim.  Dimension reduction: NA |
| Transfer data for projection | Data sources: CMIP6 future climate from Worldclim (https://www.worldclim.org/data/cmip6/cmip6_clim30s.html)  Spatial resolution: 30 arc-second  Temporal extent: 2040, 2060, 2080 and 2100  Models and scenarios: GISS E2-1-G in the 245 scenario  Data processing: layers were transformed to ASCII file and named same like in present layers. Same solar radiation and vapor pressure water variables were used in current and future models. |
| ***Model*** | |
| Multicollinearity | Removed using the *vifcor* function of the *usdm* package in RStudio (Naimi,2017). For the remaining variables, a Spearman correlation (rho > 0.70) was applied to identify those to be used in the generation of the distribution models. |
| Model settings | Otuput format: logistic, output file type: bil, do jackknife to measure variable importance, 500 iterations, 10 replicates, cross validated replicate type, *Extrapolate* and *Do clamping* boxes were deactivated. |
| Model estimates | Coefficients: mean  Variable importance: calculated with jackknife analyses |
| Threshold selection | Maximum training sensitivity plus specificity |
| ***Assessment*** | |
| Performance statistics | Performance on test data: AUC values and Partial ROC test were used as model predictive performance on validation data. |
| Plausibility check | Response plots: plots for the most important variables were verified.  Expert judgement: maps of modelled predictions were verified by a specialist taxonomist. |
| ***Prediction*** | |
| Prediction output | Prediction unit: suitability cells  Post-processing steps: the BIL files obtained from Maxent were reclassified in Arcmap into 2 classes: 0 (from zero to the logistic value of the threshold) and 1 (up to 0.9999). |
| Uncertainty quantification | NA |

# **Table S2.5**. Variables used in each species model after removal of collinearity and non-correlation.

| Species | Bioclimatic (Bio...) | | | | | | | | | | | | | | | | | | |
| --- | --- | --- | --- | --- | --- | --- | --- | --- | --- | --- | --- | --- | --- | --- | --- | --- | --- | --- | --- |
|  | 1 | 2 | 3 | 4 | 5 | 6 | 7 | 8 | 9 | 10 | 11 | 12 | 13 | 14 | 15 | 16 | 17 | 18 | 19 |
| *Pinus arizonica* | |  | X |  |  | X | X | X | X |  | X |  |  |  | X |  | X |  |  |
| *P. brachyptera* | X | X | X |  |  | X |  |  | X | X | X | X |  | X |  |  | X |  |  |
| *P. cembroides* | X |  | X |  |  | X | X | X | X |  |  |  |  | X |  | X |  | X | X |
| *P. chihuahuana* |  |  |  |  |  | X | X |  |  |  |  |  |  | X | X |  |  | X | X |
| *P. cooperi* |  | X | X | X |  | X | X | X | X |  |  |  |  |  | X |  | X | X |  |
| *P. devoniana* |  |  |  | X |  | X |  |  | X |  |  |  | X | X | X |  |  | X |  |
| *P. discolor* | X | X |  |  |  | X | X |  | X |  | X |  |  | X | X |  |  | X |  |
| *P. douglasiana* |  | X | X | X | X |  | X |  | X |  |  |  |  | X | X |  | X | X | X |
| *P. durangensis* |  |  |  |  |  | X |  | X | X |  |  |  |  |  | X |  | X | X | X |
| *P. engelmannii* |  |  | X |  |  |  |  | X | X |  | X |  |  | X | X |  | X |  | X |
| *P. herrerae* |  | X | X |  |  | X | X | X | X |  |  |  | X | X |  |  | X | X | X |
| *P. leiophylla* | X |  | X |  |  | X |  | X | X |  |  |  |  | X | X |  | X | X | X |
| *P. lumholtzii* |  |  | X |  |  |  |  | X | X |  | X |  | X | X | X | X | X | X | X |
| *P. luzmariae* |  |  | X |  |  | X |  | X | X |  |  |  |  | X | X |  |  | X |  |
| *P. maximinoi* |  | X | X |  | X |  |  |  | X |  |  |  |  | X | X |  | X | X | X |
| *P. oocarpa* |  | X | X |  |  |  | X | X | X |  |  |  | X | X | X | X | X |  | X |
| *P. strobiformis* |  |  | X |  |  | X | X | X | X |  |  |  |  |  | X |  | X | X | X |
| *P. teocote* |  | X |  |  |  | X |  | X | X |  |  |  |  | X | X |  |  | X |  |
| *P. yecorensis* |  | X |  | X |  |  | X |  |  |  |  | X |  | X |  |  | X | X |  |

***(Cont.)***

| Species | Solar radiation | | | | | Water vapor preasure | | | | | Topographic | | |
| --- | --- | --- | --- | --- | --- | --- | --- | --- | --- | --- | --- | --- | --- |
|  | max | mean | min | range | std | max | mean | min | range | std | aspect | slope | elev |
| *Pinus arizonica* | | X | X |  |  |  | X |  |  |  |  |  | X |
| *P. brachyptera* |  |  | X |  |  |  | X |  |  |  |  |  | X |
| *P. cembroides* |  | X | X |  |  |  | X |  |  | X |  |  | X |
| *P. chihuahuana* |  | X | X |  |  |  | X |  |  | X |  |  |  |
| *P. cooperi* | X |  | X |  | X |  | X |  | X |  |  |  | X |
| *P. devoniana* |  | X |  |  | X |  |  |  |  |  |  |  |  |
| *P. discolor* |  | X | X |  |  |  | X |  | X |  |  |  |  |
| *P. douglasiana* | X |  | X |  | X |  | X |  |  | X |  |  |  |
| *P. durangensis* |  |  |  |  |  |  |  |  |  |  |  |  | X |
| *P. engelmannii* |  | X | X |  |  | X | X |  |  |  |  |  | X |
| *P. herrerae* |  | X | X |  |  |  |  |  | X |  |  |  | X |
| *P. leiophylla* |  | X | X |  |  | X |  |  |  | X |  |  | X |
| *P. lumholtzii* | X | X | X |  |  |  | X |  |  | X |  |  | X |
| *P. luzmariae* | X | X |  |  | X |  |  |  | X |  |  |  |  |
| *P. maximinoi* |  | X | X |  |  |  | X |  | X |  |  |  |  |
| *P. oocarpa* | X |  | X |  |  |  | X |  | X |  |  |  |  |
| *P. strobiformis* |  | X | X |  |  |  |  |  |  |  |  |  | X |
| *P. teocote* | X | X | X |  | X |  |  |  |  | X |  |  | X |
| *P. yecorensis* |  | X | X |  |  |  | X |  | X |  |  |  |  |
